# A programmable Cas9-serine recombinase fusion protein that operates on DNA sequences in mammalian cells

**DOI:** 10.1093/nar/gkw707

**Published:** 2016-08-11

**Authors:** Brian Chaikind, Jeffrey L. Bessen, David B. Thompson, Johnny H. Hu, David R. Liu

**Affiliations:** 1 Department of Chemistry & Chemical Biology, Harvard University, Cambridge, MA 02138, USA; 2 Howard Hughes Medical institute, Harvard University, Cambridge, MA 02138 USA; 3 Wyss Institute for Biologically Inspired Engineering, Harvard University, Cambridge, MA 02138, USA

## Abstract

We describe the development of ‘recCas9’, an RNA-programmed small serine recombinase that functions in mammalian cells. We fused a catalytically inactive dCas9 to the catalytic domain of Gin recombinase using an optimized fusion architecture. The resulting recCas9 system recombines DNA sites containing a minimal recombinase core site flanked by guide RNA-specified sequences. We show that these recombinases can operate on DNA sites in mammalian cells identical to genomic loci naturally found in the human genome in a manner that is dependent on the guide RNA sequences. DNA sequencing reveals that recCas9 catalyzes guide RNA-dependent recombination in human cells with an efficiency as high as 32% on plasmid substrates. Finally, we demonstrate that recCas9 expressed in human cells can catalyze *in situ* deletion between two genomic sites. Because recCas9 directly catalyzes recombination, it generates virtually no detectable indels or other stochastic DNA modification products. This work represents a step toward programmable, scarless genome editing in unmodified cells that is independent of endogenous cellular machinery or cell state. Current and future generations of recCas9 may facilitate targeted agricultural breeding, or the study and treatment of human genetic diseases.

## INTRODUCTION

Efficient, programmable and site-specific homologous recombination remains a longstanding goal of genetics and genome editing (1). An enzyme that catalyzes recombination at sites specified by the researcher would be a valuable tool for studying the phenotypic effects of genetic alterations, enabling gene integration or gene deletion-based therapeutic strategies or exchanging genes in a locus-specific manner during agricultural breeding. Early attempts at directing recombination to loci of interest relied on the transfection of donor DNA with long flanking sequences that are homologous to a target locus (2,3). This strategy was hampered by very low efficiency and thus, the need for a stringent selection to identify integrants. More recent efforts have exploited the ability of double-stranded DNA breaks (DSBs) to induce homology-directed repair (HDR). Homing endonucleases (4) and later programmable endonucleases such as zinc finger nucleases (5), TALE nucleases (6,7), Cas9 (8,9) and fCas9 (10,11) have been used to introduce targeted DSBs and induce HDR in the presence of donor DNA. In most post-mitotic cells, however, DSB-induced HDR is strongly down regulated and generally inefficient (12). Moreover, repair of DSBs by error-prone repair pathways such as non-homologous end-joining (NHEJ) or single-strand annealing (SSA) causes random insertions or deletions (indels) of nucleotides at the DSB site (12–14) at a higher frequency than HDR. The efficiency of HDR can be increased if cells are subjected to conditions forcing cell-cycle synchronization or if the enzymes involved in NHEJ are inhibited (15–17). However, such conditions can be highly perturbative to cells, limiting potential applications.

As a complementary approach, site-specific recombinases directly catalyze the cleavage, strand exchange and religation of two double-stranded DNA sequences. Recombination can result in the insertion, deletion or inversion of sequences of interest, depending on the relative orientation of the substrate sequences. In contrast with DNA nucleases, direct catalysis by recombinases typically does not provoke error-prone DNA repair processes that result in indel formation, is not dependent on endogenous cellular DNA repair machinery, and therefore leads primarily to a single defined product. Recombinase-mediated genome modification therefore can create more precise and predictable genomic alterations than nuclease-based genome editing, and has the potential to be more efficient in non-dividing cells.

Tyrosine and serine recombinases such as Cre, Flp and ΦC31 integrase have been widely used to catalyze the recombination of exogenous DNA into model organisms (18,19). However, the use of these enzymes has been limited by their intrinsic, non-programmable DNA sequence specificity. Most small serine recombinases, for example, recognize a specific pseudo-palindromic core DNA sequence of approximately 20 base pairs (20). Recombination using these enzymes at endogenous DNA sequences only occurs at ‘pseudo-sites’ that resemble the recombinase's natural DNA recognition sequence or at genomic sequences for which the recombinase has been experimentally evolved (19,21–26).

To increase the number of sites amenable for targeted recombination in cells, researchers have fused hyperactive variants of small serine recombinases to zinc finger and TALE DNA-binding proteins (27–31). Because the catalytic domain and DNA-binding domain are partially modular in some recombinases, replacement of the natural DNA-binding domains with zinc-finger or TALE repeat arrays can partially retarget these enzymes to specified DNA sequences. Although the guide RNA-programmed Cas9 nuclease has quickly grown in popularity due to its relatively unrestricted DNA binding requirements and its ease of use, a guide RNA-programmed recombinase has not been reported.

Here, we describe the development of recCas9, a guide RNA-programmed small serine recombinase based on the fusion of an engineered Gin recombinase catalytic domain with a catalytically inactive Cas9. The recCas9 enzyme operates on a minimal pseudo-core recombinase site (NNNNAAASSWWSSTTTNNNN) flanked by two guide RNA-specified DNA sequences. Recombination mediated by recCas9 is dependent on both guide RNAs, resulting in orthogonality among different guide RNA:recCas9 complexes, and functions efficiently in cultured human cells on DNA sequences matching those found in the human genome. The recCas9 enzyme can also operate directly on the genome of cultured human cells, catalyzing a deletion between two recCas9 psuedosites located approximately 14 kb apart. This work represents a key step toward engineered enzymes that directly and cleanly catalyze gene insertion, deletion, inversion or chromosomal translocation with user-defined, single base-pair resolution in unmodified genomes.

## MATERIALS AND METHODS

### Oligonucleotides and enzymes

All oligonucleotides were purchased from Integrated DNA Technologies (IDT, Coralville, CA, USA) and are listed in the supporting material (Supplementary Tables S1–S5). Enzymes, unless otherwise noted, were purchased from New England Biolabs (Ipswich, MA, USA). Plasmid Safe ATP-dependent DNAse was purchased from Epicentre (Madison, WI, USA). All assembled vectors were transformed into One Shot Mach1-T1 phage-resistant chemically competent cells (Fisher Scientific, Waltham, MA, USA). Unless otherwise noted, all PCR reactions were performed with Q5 Hot Start High-Fidelity 2X Master Mix. Phusion polymerse was used for circular polymerase extension cloning (CPEC) assemblies.

### Plasmids

Unless otherwise stated, DNA fragments were isolated from agarose gels using the QIAquick Gel Extraction Kit (Qiagen, Valencia, CA, USA) and further purified using the DNA Clean & Concentrator-5 (Zymo Research, Irvine, CA, USA) or Qiaquick PCR purification kit (Qiagen, Valencia, CA, USA). Polymerase chain reaction (PCR) fragments not requiring gel purification were isolated using one of the kits listed above.

The pCALNL-GFP subcloning vector, pCALNL-EGFP-Esp3I, was used to clone all recCas9 reporter plasmids and was based on the previously described pCALNL-GFP vector (32). To create pCALNL-EGFP-Esp3I, pCALNL-GFP vectors were digested with XhoI and MluI and gel purified to remove the loxP sites, the kanamycin resistance marker and the poly-A terminator. Annealed oligonucleotides formed an EspI-Insert, that contained inverted Esp3I sites as well as XhoI and MluI compatible overhangs; this insert was ligated into the XhoI and MluI digested plasmid and transformed.

pCALNL-EGFP recCas9 reporter plasmids were created by Golden Gate assembly with annealed oligonucleotides and PCR products containing compatible Esp3I overhangs. Golden Gate reactions were set up and performed as described previously with Esp3I (ThermoFisher Scientific, Waltham, MA, USA) (33). The supporting material Supplementary Figure S1 outlines the general assembly scheme; relevant primers for reporter assembly as well as sequences for all recCas9 target sites are listed in the supporting materials (Supplementary Tables S2 and S6, respectively). A representative DNA sequence containing *KanR* and a PolyA terminator flanked by two recCas9 target sites is shown in the supporting material (Supplementary Note S1).

Plasmids containing the recCas9 gene were constructed by PCR amplification of a gBlock encoding an evolved, hyperactivated Gin variant (Ginβ) (34) with the oligonucleotides 1GGS-rev-BamHI or 2GGS-rev-BamHI and Gin-for-NotI. PCR fragments were digested with BamHI and NotI, purified and ligated into a previously described expression vector (Addgene plasmid 43861) (35) to produce subcloning vectors pGin-1GGS and pGIN-2GGS. Oligonucleotides 1GGS-link-for-BamHI, 5GGS-link-for-BamHI or 8GGS-link-for-BamHI were used with Cas9-rev-FLAG-NLS-AgeI to construct PCR fragments encoding Cas9-FLAG-NLS with a 1, 5 or 8 GGS linker. For DNA sequences encoding the amino acid linkers see the supporting material (Supplementary Table S7). PCR fragments and subcloning plasmids were digested with BamHI and AgeI and ligated to create plasmids pGinβ-2xGGS-dCas9-FLAG-NLS, pGinβ-5xGGS-dCas9-FLAG-NLS and pGinβ-8xGGS-dCas9-FLAG-NLS. For the DNA and amino acid sequence of the pGinβ-8xGGS-dCas9-FLAG-NLS (i.e. recCas9), see the supporting material (Supplementary Note S2).

For plasmid sequencing experiments, the AmpR gene in pGinβ-8xGGS-dCas9-FLAG-NLS was replaced with SpecR by golden gate cloning with PCR fragments. Esp3I sites were introduced into the pGinβ-8xGGS-dCas9-FLAG-NLS plasmid at sites flanking the AmpR gene by PCR with Esp3I-for-plasmid and Esp3I-rev-plasmid. The primers spec-Esp3I-for and spec-Esp3I-rev were used to amplify the SpecR marker as well as introduce Esp3I sites and Esp3I generated overhangs compatible with those generated by the Esp3I-cleaved plasmid PCR product. Golden gate assembly was performed on the two fragments following the protocol used to generate the reporter plasmids (see Methods in the Supplementary Data).

The pHU6-NT1 guide RNA expression vector was based on the previously described pFYF1328 (35) altered to target a region within the bacterial luciferase gene LuxAB. Guide RNA expression vectors were created by PCR amplification of the entire vector with a universal primer R.pHU6.TSS(-1).univ and primers encoding unique guide RNA sequences (Supplementary Table S1). See the supporting material for a list of the guide RNA sequences (Supplementary Table S8). These primers were phosphorylated with T4 polynucleotide kinase. The PCR reaction products and linear guide RNA expression vectors were blunt-end ligated and transformed. Guide RNA expression vectors used in initial optimizations, off target control guide RNA sequences and those targeting Chromosome 10 locus contained AmpR. All other plasmids described in this study contained specR to facilitate sequencing experiments. Spectinomycin resistance was initially introduced into guide RNA expression vectors via CPEC essentially as described (36,37) and guide RNA plasmids were then constructed by PCR amplification of the vector, as described above. Reactions were incubated overnight at 37°C with 40 U of DpnI, purified and transformed. Fragments for CPEC were generated by PCR amplification of a guide RNA expression vector with oligonucleotides cpec-assembly-for-spec2 and cpec assembly-rev. The specR fragment was generated by PCR amplification of the SpecR gene via the oligonucleotides cpec-assembly-for-spec and cpec-assembly-rev-spec. pUC19 (ThermoFisher Scientific, Waltham, MA, USA) was similarly modified.

### Cell culture and transfection

HEK293T cells were purchased from the American Type Culture Collection (ATCC, Manassas, VA, USA). Cells were cultured in Dulbecco's modified Eagle's medium (DMEM) + GlutaMAX-I (4.5 g/l D glucose +110 mg/ml sodium pyruvate) supplemented with 10% fetal bovine serum (FBS, Life Technologies, Carlsbad, CA, USA). Cells were cultured at 37°C at 5% CO_2_ in a humidified incubator.

Plasmids used for transfections were isolated from PureYield Plasmid Miniprep System (Promega, Madison, WI, USA). The night before transfections, HEK293T cells were seeded at a density of 3 × 10^5^ cells per well in 48 well collagen-treated plates (Corning, NY, USA). Transfection reactions were prepared in 25 μl of Opti-MEM (ThermoFisher Scientific, Waltham, MA, USA). For each transfection, 45 ng of each guide RNA expression vector, 9 ng of reporter plasmid, 9 ng of piRFP670-N1 (Addgene Plasmid 45457) and 160 ng of recCas9 expression vector were mixed, combined with 0.8 μl lipofectamine 2000 in Opti-MEM (ThermoFisher Scientific, Waltham, MA, USA) and added to individual wells.

### Flow cytometry

After 60–72 h post-transfection, cells were washed with phosphate buffered saline and detached from plates by the addition of 50 μl of 0.05% trypsin-EDTA (Life Technologies, Carlsbad, CA, USA) at 37°C for 5–10 min. Cells were diluted in 250 μl culture media and run on a BD Fortessa analyzer. iRFP fluorescence was excited using a 635 nm laser and emission was collected using a 670/30 band pass filter. EGFP was excited using a 488 nM laser and emission fluorescence acquired with a 505 long pass and 530/30 band pass filters. Data were analyzed on FlowJo Software, gated for live and transfected events (expressing iRFP). Positive EGFP-expressing cells were measured as a percentage of transfected cells gated from at least 6000 live events. For optimization experiments, assay background was determined by measuring the percentage of transfected cells producing EGFP upon cotransfection with reporter plasmid and pUC, without recCas9 or guide RNA expression vectors. This background was then subtracted from percentage of EGFP-positive cells observed when the reporter plasmid was cotransfected with recCas9 and the on-target or non-target guide RNA expression vectors.

### Identification of genomic target sites

Searching for appropriate target sites was done using Bioconductor, an open-source bioinformatics package using the R statistical programming (38). The latest release (GRCh38) of the human reference genome published by the Genome Reference Consortium was used to search for sites that matched both the PAM requirement of Cas9 and the evolved gix sequence as described in the text. With the genome loaded into R, each search pattern was represented as a Biostring, a container in R that allowed for string matching and manipulation (Supplementary Note S3). Scanning both strands of DNA for the entire genome, using the stated parameters, reveals ∼450 potential targets in the human genome when searching using the GRCh38 reference assembly (Supplementary Table S9).

### DNA sequencing

Transfections of HEK293T cells were performed as above in sextuplet and incubated for 72 h. Cells were harvested and replicates were combined. Episomal DNA was extracted using a modified HIRT extraction involving alkaline lysis and spin column purification essentially as described (39,40). Briefly, after harvesting, HEK293T cells were washed in 500 μl of ice cold PBS, resuspended in 250 μl GTE Buffer (50 mM glucose, 25 mM Tris-HCl, 10 mM EDTA and pH 8.0) and lysed on ice for 5 min in lysis buffer (200 mM NaOH, 1% sodium dodecyl sulfate). Lysis was neutralized with neutralization buffer (5 M acetate, 3 M potassium, pH 6.7). Cell debris were pelleted by centrifugation at 21,130 *g* for 15 min and lysate was applied to EconoSpin columns (Epoch Life Science, Missouri City, TX, USA). Columns were washed twice with 750 μl wash buffer (Omega Bio-tek, Norcross, GA, USA) and eluted in 45 μl TE buffer, pH 8.0.

Isolated episomal DNA was digested for 2 h at 37°C with RecBCD (10 U) following the manufacturer's instructions and purified into 10 μl EB with a MinElute Reaction Cleanup Kit (Qiagen, Valencia, CA, USA). Mach1-T1 chemically competent cells were transformed with 5 μl of RecBCD-digested episomal DNA (see above) and plated on agarose plates containing 50 μg/ml carbenicillin. Individual colonies were sequenced with primer pCALNL-for-1 to determine the rate of recombination.

### Analysis of recCas9 catalyzed genomic deletions

HEK293T cells were seeded at a density of 6 × 10^5^ cells per well in 24 well collagen-treated plates and grown overnight (Corning, NY, USA). Transfections reactions were brought to a final volume of 100 μl in Opti-MEM (ThermoFisher Scientific, Waltham, MA, USA). For each transfection, 90 ng of each guide RNA expression vector, 20 ng of pmaxGFP (Lonza, Allendale, NJ, USA) and 320 ng of recCas9 expression vector were combined with 2 μl Lipofectamine 2000 in Opti-MEM (ThermoFisher Scientific, Waltham, MA, USA) and added to individual wells. After 48 h, cells were harvested and sorted for the GFP transfection control on a BD FACS AriaIIIu cell sorter. Cells were sorted on purity mode using a 100 μm nozzle and background fluorescence was determined by comparison with untransfected cells. Sorted cells were collected on ice in phosphate buffered saline (PBS), pelleted and washed twice with ice cold PBS. Genomic DNA was harvested using the E.Z.N.A. Tissue DNA Kit (Omega Bio-Tek, Norcross, GA, USA) and eluted in 100 μl EB. Genomic DNA was quantified using the Quant-iT PicoGreen dsDNA kit (ThermoFisher Scientific, Waltham, MA, USA) measured on a Tecan Infinite M1000 Pro fluorescence plate reader.

Nested PCR was carried out using Q5 Hot Start Polymerase 2x Master Mix supplemented with 3% DMSO and diluted with HyClone water, molecular biology grade (GE Life Sciences, Logan, UT, USA). Primary PCRs were carried out at 25 μl scale with 20 ng of genomic DNA as template using the primer pair FAM19A2-F1 and FAM19A2-R1 (Supplementary Table S5). The primary PCR conditions were as follows: 98°C for 1 min, 35 cycles of (98°C for 10 s, 59°C for 30 s, 72°C for 30 s), 72°C for 1 min. A 1:50 dilution of the primary PCR served as template for the secondary PCR, using primers FAM19A2-F2 and FAM19A2-R2. The secondary PCR conditions were as follows: 98°C for 1 min, 30 cycles of (98°C for 10 s, 59°C for 20 s, 72°C for 20 s), 72°C for 1 min. DNA was analyzed by electrophoresis on a 1% agarose gel in TAE alongside a 1 Kb Plus DNA ladder (ThermoFisher Scientific, Waltham, MA, USA). Material to be Sanger sequenced was purified on a Qiagen Minelute column (Valencia, CA, USA) using the manufacturer's protocol. Template DNA from 3 biological replicates was used for three independent genomic nested PCRs.

The limit of detection was calculated given that one complete set of human chromosomes weighs approximately 3.6 pg $( {3.3 \cdot {{10}^9}\;{\rm{bp}} \times 1 \cdot {{10}^{ - 21}}\frac{8}{{{\rm{bp}}}}} )$. Therefore, a PCR reaction seeded with 20 ng of genomic DNA template contains ∼5500 sets of chromosomes.

For quantification of genomic deletion, nested PCR was carried out using the above conditions in triplicate for each of the 3 biological replicates. A 2-fold dilution series of genomic DNA was used as template, beginning with the undiluted stock (for sample 1, 47.17 ng/µl; for sample 2, 75.96 ng/µl; and for sample 3, 22.83 ng/µl) to reduce potential sources of pipetting error. The lowest DNA concentration for which a deletion PCR product could be observed was assumed to contain a single deletion product per total genomic DNA. An example calculation can be found in the supporting material Note S4.

## RESULTS

### Fusing Gin recombinase to dCas9

**Figure 1.**
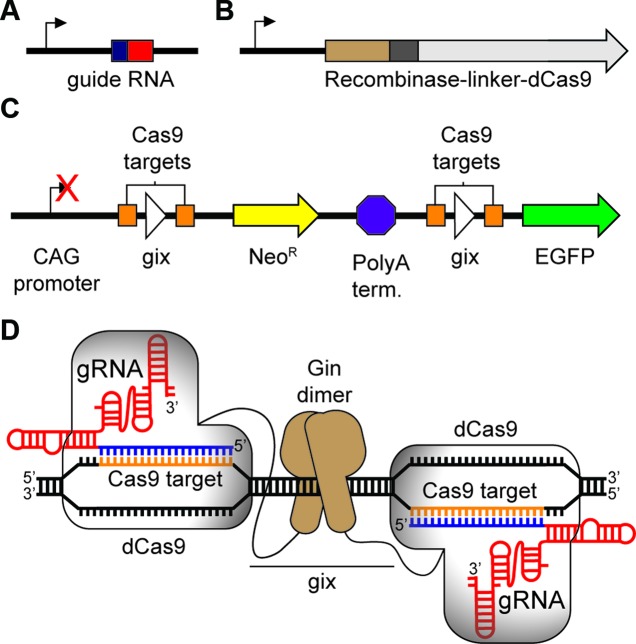
Overview of experimental setup. Cells are transfected with (**A**) guide RNA expression vector(s) under the control of hU6 promoter, (**B**) a Cas9-ginβ expression vector under the control of a CMV promoter and (**C**) a recCas9 reporter plasmid. Cotransfection of these components should result in (**D**) reassembly of guide RNA-programmed recCas9 at the target sites. This will mediate deletion of the polyA terminator, allowing transcription of *EGFP*. Guide RNA expression vectors and guide RNA sequences are abbreviated as gRNA.

Our group and others recently demonstrated that the N-terminus of dCas9 could be fused to the FokI nuclease catalytic domain, resulting in a dimeric dCas9-FokI fusion that cleaved DNA sites flanked by two guide RNA-specified sequences (10,11). We used the same fusion orientation to connect dCas9 to Ginβ, a highly active catalytic domain of dimeric Gin invertase previously evolved by Barbas *et al.* (34). Ginβ promiscuously recombines several 20-bp core ‘gix’ sequences (34) related to the native core sequence CTGTAAACCGAGGTTTTGGA (41–43). We envisioned that the guide RNAs would localize a recCas9 dimer to a gix site flanked by two guide-RNA specified sequences, enabling the Ginβ domain to catalyze DNA recombination in a guide RNA-programmed manner (Figure 1D).

To assay the resulting dCas9-Ginβ (recCas9) fusions, we constructed a reporter plasmid containing two recCas9 target sites flanking a poly-A terminator that blocks *EGFP* transcription (Figure 1A–C). Each recCas9 target site consists of a gix core pseudo-site flanked by sites matching a guide RNA protospacer sequence. Recombinase-mediated deletion removes the terminator, restoring transcription of *EGFP*. We cotransfected HEK293T cells with this reporter plasmid, a plasmid transcribing a guide RNA(s) and a plasmid producing candidate dCas9-Ginβ fusion proteins, and used the fraction of cells exhibiting EGFP fluorescence to assess the relative activity of each fusion construct.

**Figure 2.**
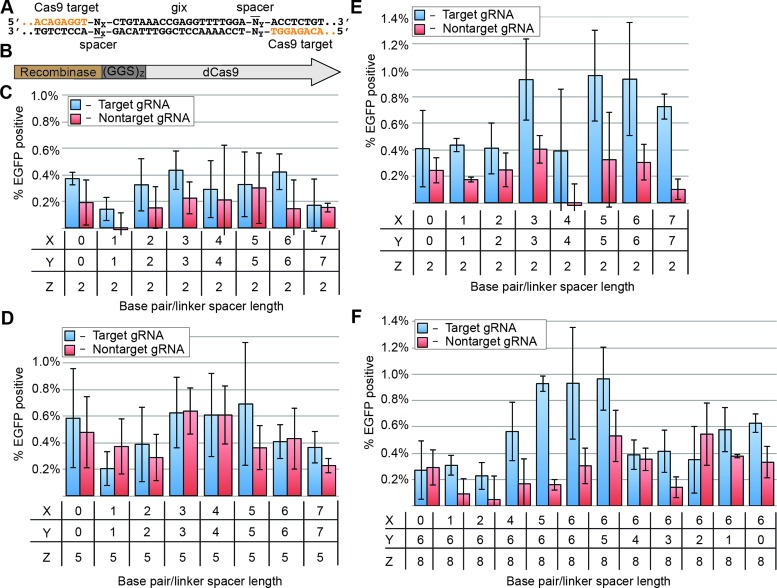
Optimization of fusion linker lengths and target site spacer variants. A single target guide RNA expression vector, pHU6-NT1 or non-target vector pHU6-BC74 was used in these experiments. See supporting material, Tables S6–S8, for sequences. (**A**) A portion of the target site is shown with guide RNA target sites in orange and a gix pseudo site in black. The 5′ and 3′ sequences on either side of the pseudo-gix sites are identical, but inverted, and are recognized by pHU6-NT1. The number of base pair spacers separating the gix pseudo-site from the 5′ and 3′ binding sites is represented by an X and Y, respectively. (**B**) Z represents the number of GGS repeats connecting Ginβ to dCas9. Fusion protein activity is assessed with X = Y for (**C**) (GGS)_2_ (**D**) (GGS)_5_ and (**E**) (GGS)_8_ linkers (i.e. recCas9) connecting the Gin catalytic domain to the dCas9 domain. (**F**) The activity of recCas9 on target sites composed of uneven base pair spacers (X≠Y) was determined; X = Y = 6 is included for comparison. All experiments are performed in triplicate and background fluorescence is subtracted from these experiments (see Materials and Methods). The percentage of EGFP-positive cells shown is of transfected cells (determined by gating for the presence of a cotransfected plasmid constitutively expressing *iRFP*) and at least 6000 live events are recorded for each experiment. Guide RNA expression vectors and guide RNA sequences are abbreviated as gRNA. Values and error bars represent the mean and standard deviation, respectively, of three independent biological replicates.

We varied parameters influencing the architecture of the recCas9 components, including the spacing between the core gix site and the guide RNA-binding site (from 0 to 7 bp), as well as linker length between the dCas9 and Ginβ moieties ((GGS)_2_, (GGS)_5_ or (GGS)_8_) (Figure 2A–F). Most fusion architectures resulted in no observable guide RNA-dependent *EGFP* expression (Figure 1C and D). However, one fusion construct containing a linker of eight GGS repeats and 3- to 6-base pair spacers resulted in approximately 1% recombination when a matched, but not mismatched, guide RNA was present (Figure 2E and F). Recombination activity was consistently higher when 5–6 base pairs separated the dCas9 binding sites from the core (Figure 2F). These results collectively reveal that specific fusion architectures between dCas9 and Ginβ can result in guide RNA-dependent recombination activity at spacer-flanked gix pseudo core sites in human cells. We refer to this 8xGGS linker fusion construct as ‘recCas9’.

### Targeting DNA sequences found in the human genome with recCas9

We hypothesized that low levels of observed activity may be caused by a suboptimal guide RNA sequence or core gix sequence, consistent with previous reports showing that the efficiency of guide RNA:Cas9 binding is sequence-dependent (44). Moreover, although our optimization was conducted with the native gix core sequence (41–43), several studies have shown that zinc finger-Gin or TALE-Gin fusions are active, and in some cases more active, on slightly altered core sites (28,30,31,34,45–47). Thus, we next sought to target sequences found within the human genome to test if unmodified human genomic sequences were capable of being targeted by recCas9 and to test if varying the guide RNA and core sequences would increase recCas9 activity.

To identify potential target sites, we used previous findings that characterized evolved Gin variants (34) as well as our above observations. Using this information, we searched the human genome for sites that contained CCN_(30–31)_-AAASSWWSSTTT-N_(30–31)_-GG, where W is A or T, S is G or C, and N is any nucleotide. The N_(30-31)_ includes the N of the NGG protospacer adjacent motif (PAM), the 20-base pair Cas9 binding site, a 5- to 6-base pair spacing between the Cas9 and gix sites, and the four outermost base pairs of the gix core site. The internal 12 base pairs of the gix core site (AAASSWWSSTTT) were previously determined to be important for Ginβ activity (34).

**Figure 3.**
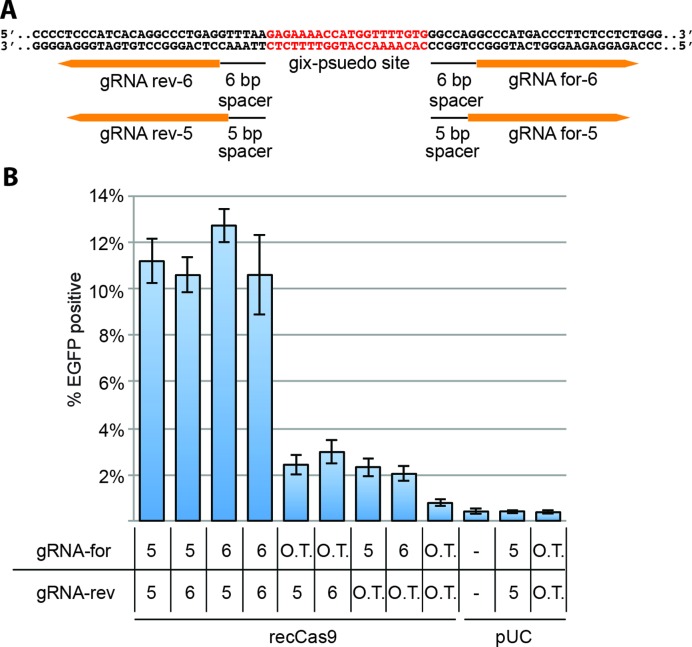
The dependence of forward and reverse guide RNAs on recCas9 activity. (**A**) A sequence, found within *PCDH15* replaces the target site tested in Figure 1. Two offset sequences can be targeted by guide RNAs on both the 5′ and 3′ side of a pseudo-gix core site. (**B**) Activity is measured by cotransfecting a recCas9 expression vector and reporter plasmid with all four guide RNA expression vector pairs as well as individual guide RNA vectors with off-target (O.T.) guide RNA vectors. The off-target forward and reverse guide RNA sequences target *CLTA* and *VEGF*, respectively (10). Control experiments transfected with the reporter plasmid but without target guide RNA vectors are also shown. The results of reporter plasmid cotransfected with different guide RNA expression vectors, but without recCas9 expression vectors, are also shown. The percentage of EGFP-positive cells is of only those transfected cells (by gating for the presence of a cotransfected plasmid constitutively expressing *iRFP*) and at least 6000 live events are recorded for each experiment. Guide RNA expression vectors and guide RNA sequences are abbreviated as gRNA. Values and error bars represent the mean and standard deviation, respectively, of three independent biological replicates and background fluorescence is not subtracted from these experiments.

Our search revealed ∼450 such loci in the human genome (Supplementary Table S9). We created a reporter construct that contains the sequence identical to one of these genomic loci, found in *PCDH15*, and constructed guide RNA expression vectors to direct recCas9 to this sequence (Figure 3A). These vectors encoded two pairs of guide RNAs, each of which contain spacer sequences that match the 5′ and 3′ regions flanking the *PCDH15* psuedo gix sites. Co-transfection of the reporter plasmid, combinations of these flanking guide RNA expression vectors, and the recCas9 expression vector resulted in *EGFP* expression in 11–13% of transfected cells (Figure 3B), representing a >10-fold improvement in activity over the results shown in Figure 2. These findings demonstrate that a more judicious choice of recCas9 target sequences can result in substantially improved recombination efficiency at DNA sequences matching those found in the human genome.

Next we sought to determine if both guide RNA sequences were required to cause recCas9-mediated deletion. We co-transfected into HEK293T cells just one of the guide RNA vectors targeting the 5′ or 3′ flanking sequences of the *PCDH15* psuedo-gix core site, the *PCDH15* reporter plasmid and a recCas9 expression vector. These co-transfections resulted in 2.5–3% EGFP expression (Figure 3B). We speculate that the low levels of activity observed upon expression of just one of the targeting guide RNAs and recCas9 may be caused by the propensity of hyperactivated gix monomers to form dimers (48); transient dimerization may occasionally allow a single protospacer sequence to localize the dimer to a target site. No activity was detected above background when using off-target guide RNA vectors or when the recCas9 vector was replaced by pUC (Figure 3B).

These findings demonstrate that recCas9 activity can be increased substantially over the modest activity observed in our initial experiments by choosing different target sites and matching guide RNA sequences. We observed a >10-fold increase in activity on the *PCDH15* site compared to the original target sequences (compare Figure 3B with Figure 2F). Further, maximal recombination activity is dependent on the presence of both guide RNAs and recCas9.

### Orthogonality of recCas9

Next, we sought to test if recCas9 could target, in an orthogonal manner, multiple separate loci matching sequences found in the human genome. We selected a subset of the recCas9 target sites in the human genome based on their potential use as a safe-harbor loci for genomic integration, or in one case, based on their location within a gene implicated in genetic disease.

To identify these sites, we searched ENSEMBL (release 81) to identify which predicted recCas9 target sites fall within annotated genes (49). One such site fell within an intronic region of *FGF14*. Mutations within *FGF14* are believed to cause spinocerebellar ataxia 27 (SCA 27) (50–54). Finally, we manually interrogated a fraction of the predicted recCas9 target sites that did not fall within genes to determine if some sequences fell within safe harbor loci. Using annotations in ENSEMBL (49), we identified genomic targets that matched most of the five criteria for safe harbor loci described by Sadelain *et al.* (49,55). We constructed five reporters and corresponding guide RNA vector pairs containing sequences identical to those in the genome. To evaluate the orthogonality of recCas9 when programmed with different guide RNAs, we tested all combinations of the five guide RNA pairs with the five reporters.

**Figure 4.**
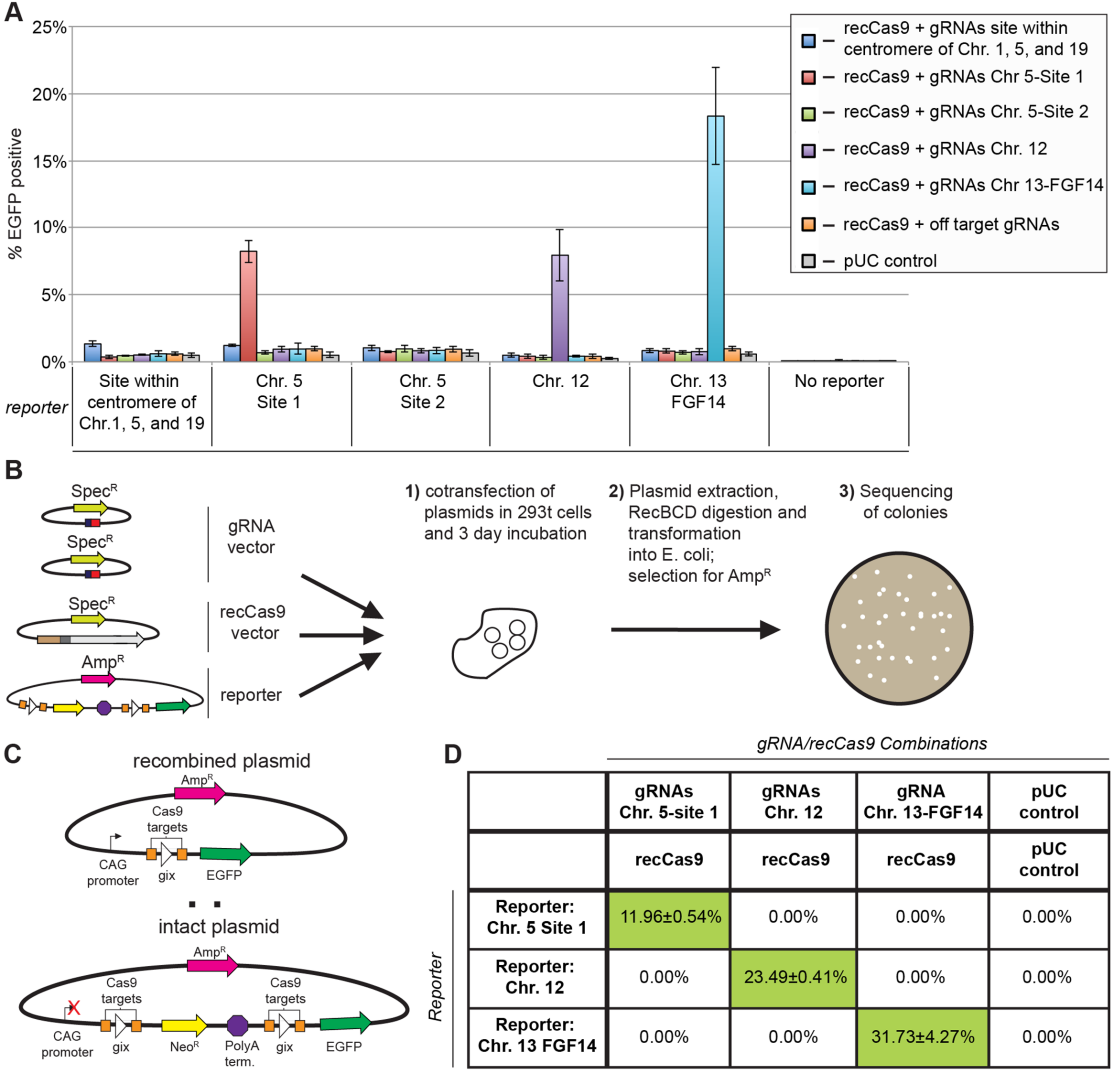
RecCas9 can target multiple sequences identical to those in the human genome. (**A**) The target sites shown in Figure 1 are replaced by sequences found within the human genome. See Table S6 from the Supplementary Data for sequences. A recCas9 expression vector was cotransformed with all combinations of guide RNA vectors pairs and reporter plasmids. Off-target guide RNA vectors were also cotransformed with the recCas9 expression vector and reporter plasmids and contain guide RNA sequences targeting CLTA and VEGF (10). The percentage of EGFP-positive cells reflects that of transfected (iRFP-positive) cells. At least 6000 live events are recorded for each experiment. Values and error bars represent the mean and standard deviation, respectively, of at least three independent biological replicates. (**B**) Transfection experiments were performed again, replacing the resistance marker in the recCas9 expression vector and pUC with SpecR. After cotransfection and incubation, episomal DNA was extracted, transformed into *E. coli* and selected for carbenicillin resistance. Colonies were then sequenced to determine (**C**) the ratio of recombined to fully intact plasmids. (**D**) Sequencing data from episomal extractions isolated from transfected cells. Columns and rows represent the transfection conditions. Each cell shows the percent of recombined plasmid, determined by dividing the number of recombined plasmids by the total number of sequenced plasmids for each condition. The values shown reflect the mean and standard deviation of two independent biological replicates. The average difference between the mean and each replicate is shown as the error. Guide RNA expression vectors and guide RNA sequences are abbreviated as gRNA.

Cotransfection of reporter, guide RNA plasmids and recCas9 expression vectors revealed that three of the five reporters tested resulted in substantial levels of EGFP-positive cells consistent with recCas9-mediated recombination. This *EGFP* expression was strictly dependent upon cotransfection with a recCas9 expression vector and guide RNA plasmids matching the target site sequences on the reporter construct (Figure 4A). The same guide RNA pairs that caused recombination when cotransfected with cognate reporter plasmids and a recCas9 vector were unable to mediate recombination when cotransfected with non-cognate reporter plasmids (Figure 4A). These results demonstrate that recCas9 activity is orthogonal and will only catalyze recombination at a gix pseudo core sites when programmed with a pair of guide RNAs matching the flanking sequences. We observed no recombinase activity above the background level of the assay when reporter plasmids were transfected without vectors expressing recCas9 and guide RNAs.

### Characterization of recCas9 products

We characterized the products of recCas9-mediated recombination of the reporter plasmids to confirm that *EGFP* expression was a result of recCas9-mediated removal of the poly-A terminator sequence. We sequenced reporter plasmids for chromosome 5-site 1, chromosome 12 and chromosome 13 (*FGF14* locus) after cotransfection with recCas9 expression vectors and with plasmids producing cognate or non-cognate guide RNA pairs. After incubation for 72 h, episomal DNA was extracted (see Methods in Supplementary Data) and transformed into *E. coli* to isolate reporter plasmids. Single colonies containing reporter plasmids were sequenced (Figure 4B).

Individual colonies were expected to contain either an unmodified or a recombined reporter plasmid (Figure 4C). For each biological replicate, we sequenced an average of 97 colonies transformed with reporter plasmid isolated from each transfection condition. Recombined plasmids were only observed if reporter plasmids were previously cotransfected with cognate guide RNA plasmids and recCas9 expression vectors (Figure 4D). In two separate experiments, the percent of recombined plasmid ranged from 12% for site 1 in chromosome 5 to an average of 32% for the *FGF14* locus in chromosome 13. The sequencing data therefore were consistent with our earlier flow cytometry analysis in Figure 4A. The absolute levels of recombined plasmid were somewhat higher than the percent of EGFP-positive cells (Figure 4). This difference likely arises because the flow cytometry assay does not report on multiple recombination events that can occur when multiple copies of the reporter plasmid are present in a single cell; even a single recombination event may result in EGFP fluorescence. As a result, the percentage of EGFP-positive cells may correspond to a lower limit on the actual percentage of recombined reporter plasmids. Alternatively, the difference may reflect the negative correlation between plasmid size and transformation efficiency (56); the recombined plasmid is approximately 5700 base pairs and may transform slightly better than the intact plasmid, which is approximately 6900 base pairs.

Since zinc finger-recombinases have been reported to cause mutations at recombinase core-site junctions (34), we tested if such mutagenesis occurs from recCas9 treatment. In our reporter construct, recCas9 should delete *kanR* and the poly-A terminator by first cleaving the central dinucleotide of both gix core sites and then religating the two cores to each other (Figure 4C). Thus, the recombination product should be a single recombination site consisting of the first half of the ‘left’ target site and the second half of the ‘right’ target site. Erroneous or incomplete reactions could result in other products. Strikingly, all of the 134 recombined sequences examined contained the expected recombination products. Further, a total of 2317 sequencing reads from two separate sets of transfection experiments revealed only three sequencing reads containing potential deletion products at otherwise non-recombined plasmids.

One of these deletion-containing reads was observed in a chromosome 12 reporter plasmid that was transfected with the pUC control and lacked both recCas9 target sites as well as the polyA terminator. We attribute this product to DNA damage that occurred during the transfection, isolation or subsequent manipulation. Because recCas9 may only localize to sequences when cotransfected with reporter and cognate guide RNA expression vectors, a more relevant metric may be to measure the total number of deletion products observed when reporter plasmids are cotransfected with cognate guide RNA vectors and recCas9 expression vectors. A single indel was observed out of a total of 185 plasmids sequenced from cotransfections with the chromosome 5-site 1 reporter and cognate guide RNA. Similarly, one indel was observed out of 204 plasmids from the chromosome 12 reporter following transfection with cognate guide RNA and recCas9 expression vectors. Notably, out of 202 sequencing reads, no indels were observed from the chromosome 13 reporter following cognate guide RNA and recCas9 cotransfection, despite resulting in the highest observed levels of recombination. These observations collectively suggest that recCas9 mediates predominantly error-free recombination.

Taken together, these results establish that recCas9 can target multiple sites found within the human genome with minimal cross-reactivity or byproduct formation. Substrates undergo efficient recombination only in the presence of cognate guide RNA sequences and recCas9, give clean recombination products in human cells, and generally do not result in mutations at the core-site junctions or products such as indels that arise from cellular DNA repair.

### RecCas9-mediated genomic deletion

Finally, we investigated whether recCas9 is capable of operating directly on the genomic DNA of cultured human cells. First, we attempted to use recCas9 to genomically integrate a plasmid containing a geneticin resistance marker and a sequence matching the chromosome 13-FGF14, chromosome 12 or chromosome 5-site 1 tested in Figure 4. However, we did not observe an increase in antibiotic resistance indicative of integration in HEK293 cells. Reasoning that deletion should be more favorable than integration, we used the list of potential recCas9 recognition sites in the human genome (Supplementary Table S9) to identify pairs of sites that, if targeted by recCas9, would yield chromosomal deletion events detectable by PCR. We designed guide RNA expression vectors that would direct recCas9 to those recCas9 sites closest to the chromosome 5-site 1 or chromosome 13 (*FGF14 locus*), sites which were both shown to be recombined in our transient transfection assays (Figure 4). The new target sites ranged from ∼3 to 23 Mbps upstream and 7 to 10 Mbps downstream of chromosome 5-site 1, and 12 to 44 Mbps upstream of the chromosome 13-FGF14 site. We cotransfected the recCas9 expression vector with each of these new guide RNA pairs and the validated guide RNA pairs used for chromosome 5-site 1 or chromosome 13-FGF14, but were unable to observe evidence of chromosomal deletions by genomic PCR.

**Figure 5.**
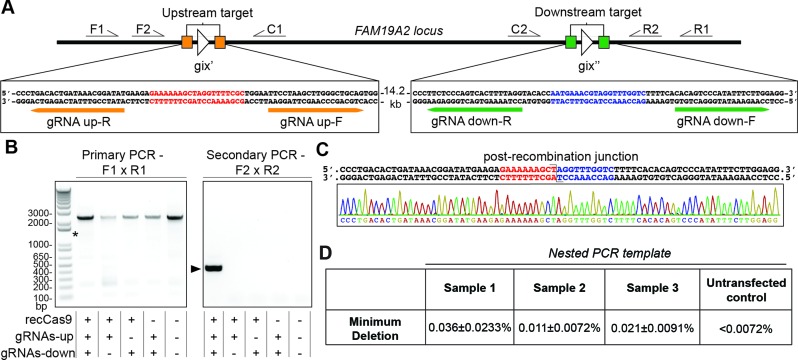
recCas9 mediates guide RNA- and recCas9-dependent deletion of genomic DNA in cultured human cells. (**A**) Schematic showing predicted recCas9 target sites located within an intronic region of the *FAM19A2* locus of chromosome 12 and the positions of primers used for nested PCR. (**B**) Representative results of nested genomic PCR of template from cells transfected with the indicated expression vectors (n = 3 biological replicates; NTC = no template control). The asterisk indicates the position of the 1.3-kb predicted primary PCR deletion product. Arrow indicates the predicted 415-bp deletion product after the secondary PCR. Both panes are from the same agarose gel, but were cut to remove blank lanes. (**C**) Sanger sequencing of PCR products resulting from nested genomic PCR of cells transfected with all four gRNA expression vectors and the recCas9 expression vector matches the predicted post-recombination product. (**D**) Estimated lower limit of deletion efficiency of *FAM19A2* locus determined by limiting-dilution nested PCR. The values shown reflect the mean and standard deviation of three technical replicates.

We reasoned that genomic deletion might be more efficient if the recCas9 target sites were closer to each other on the genome. We identified two recCas9 sites separated by 14.2 kb within an intronic region of *FAM19A2*; these sites also contained identical dinucleotide cores which should facilitate deletion. *FAM19A2* is one of five closely related TAFA-family genes encoding small, secreted proteins that are thought to have a regulatory role in immune and nerve cells (57). Small nucleotide polymorphisms located in intronic sequences of *FAM19A2* have been associated with elevated risk for systemic lupus erythematosus (SLE) and chronic obstructive pulmonary disease (COPD) in genome-wide association studies (57); deletion of the intronic regions of this gene might therefore provide insights into the causes of these diseases. We cloned four guide RNA sequences in expression vectors designed to mediate recCas9 deletion between these two *FAM19A2* sites. Vectors expressing these guide RNAs were cotransfected with the recCas9 expression vector (Figure 5A). RecCas9-mediated recombination between the two sites should result in deletion of the 14.2-kb intervening region. Indeed, we detected this deletion event by nested PCR using gene-specific primers that flank the two *FAM19A2* recCas9 targets. We observed the expected PCR product that is consistent with recCas9-mediated deletion only in genomic DNA isolated from cells cotransfected with the recCas9 and all four guide RNA expression vectors (Figure 5B). We could not detect the deletion PCR product in the genomic DNA of cells transfected without either the upstream or downstream pair of guide RNA expression vectors alone, without the recCas9 expression plasmid or for the genomic DNA of untransfected control cells (Figure 5B). Our estimated limit of detection for these nested PCR products is ∼1 deletion event per 5500 chromosomal copies. We isolated and sequenced the 415-bp PCR product corresponding to the predicted genomic deletion. Sequencing confirmed that the PCR product matched the predicted junction expected from the recombinase-mediated genomic deletion and did not contain any insertions or deletions suggestive of NHEJ (Figure 5C).

We estimated a lower limit on the minimum genomic deletion efficiency using nested PCR on the serial dilutions of genomic template (see Supplementary Note S4 or (58) for greater detail). A given amount of genomic DNA that yields the recCas9-specific nested PCR product must contain at least one edited chromosome. To establish a lower limit on this recCas9-mediated genomic deletion event, we therefore performed nested PCR on serial dilutions of genomic DNA (isolated from cells transfected with recCas9 and the four FAM19A2 guide RNA expression vectors) to determine the lowest concentration of genomic template DNA that results in a detectable deletion product. These experiments reveal a lower limit of deletion efficiency of 0.023 ± 0.017% (average of three biological replicates) (Figure 5D), suggesting that recCas9-mediated genomic deletion proceeds with at least this efficiency. Nested PCR of the genomic DNA of untransfected cells resulted in no product, with an estimated limit of detection of <0.0072% recombination. Together, these results indicate that recCas9 can mediate a targeted, seamless deletion of a native locus present within genomic DNA of cultured human cells.

## DISCUSSION

We have demonstrated that the optimized fusion of a catalytically inactive Cas9 to the hyperactive catalytic domain of a small serine invertase results in an RNA-programmed recombinase (recCas9). RecCas9 activity is dependent on the presence of both guide RNA sequences targeting sites that flank an internal pseudo gix core site. Importantly, this fusion can be directed to a variety of endogenous human genomic sequences, resulting in seamless recombination events that rarely contain indels or other mutations at recombinase junctions. Current or future generations of recCas9 may prove useful as tools to cleanly delete or integrate DNA for the study or treatment of genetic diseases, or to mediate the precise exchange of genetic material during agricultural breeding.

It is theoretically possible that recCas9 cleavage and cellular repair processes such as NHEJ may form perfect junctions that result in the expected deletion product. However, this outcome is unlikely, given that numerous studies have observed that cleavage of episomal DNA in human cells results in observable levels of error-prone repair around the cleavage site (59–61). Deletions, caused by NHEJ-mediated repair of complementary 5′ DSBs, have been reported to be as high as 66% of all repair products in a lymphoblastoid cell line (61). Even under the most stringent *in vitro* conditions, in which episomal DNA is subjected to a brief exposure of an endonuclease, 4–10% of plasmids show indel formation upon DSB repair (59). In contrast, our reporter plasmids are continuously exposed to the recCas9:guide RNA complex for 72 h. We would therefore expect that any recCas9 cleavage and cellular DSB repair would occur continuously over the course of the experiment, driving the formation of indels that are no longer recognized by the recCas9 enzyme. High levels of indel formation have been observed when episomal DNA is continuously exposed to homing endonuclease for 6–21 days (60). We examined 1947 independent plasmid sequencing reads exposed to recCas9 for 72 h and observed only two reads containing a deletion product at the target site junction. An additional single read containing a large deletion product was most likely an artifact of the episomal isolation process. The overwhelming majority of examined sequences contained either intact reporter plasmid or the expected recombination product, providing strong support that the recCas9 functions as a guide RNA-dependent recombinase.

This work represents the first step toward seamless, RNA-programmed enzymatic recombination of genomic DNA. RecCas9-catalyzed genomic integration has the potential to overcome one of the major limitations imposed by strategies that integrate DNA by HDR: in mammalian cells, DSBs are typically repaired by error-prone processes more frequently than by HDR. Although recombinase-mediated integration is a less favorable process than recombinase-mediated deletion, strategies such as recombinase-mediated cassette exchange (RMCE) have been implemented to favor genomic integration (18,62). Current RMCE strategies require that target sites for recombinases must be integrated into the model organism prior to integration of exogenous DNA. Our strategy, in principle, overcomes this limitation by demonstrating the recCas9 system is capable of targeting sequences found endogenously within the human genome.

The findings reported here provide a foundation toward RMCE on native genomic loci that would require two complete recCas9 target sites to be proximal to each other. The estimated 450 human genomic sites identified *in silico* for recCas9 might be expanded substantially by replacing the Gin recombinase catalytic domain with other natural or manmade small serine recombinases that recognize different core sequences; many of these related enzymes have also been directed to novel sites via fusion to zinc finger proteins (19,63). Moreover, recent work altering Cas9 PAM binding specificity and the recent discovery of numerous Cas9 orthologs raise the possibility of further expanding the number of potential recCas9 sites (64–67). Extending the approach developed here may eventually lead to tools capable of specific, seamless integration of exogenous DNA into the human genome.

Deletion of the *FAM19A2* intronic sequence in human cells demonstrates that recCas9 is capable of precisely modifying genomic DNA. While we carried out extensive optimization of the chimeric recCas9 to improve its activity (Figure 2), we imagine that further improvements, e.g. by evolving the chimeric fusion or using a recombinase domain with a broader sequence tolerance, may increase the activity and substrate scope of recCas9-mediated genomic modification.

Additionally, further characterization of recCas9 sequence requirements and tolerances may allow a more judicious choice of target site(s) and ultimately expand the utility of this enzyme. Such characterization may also help explain why recCas9 was not active on two of the five genomic sequences tested in our plasmid-based assays (Figure 4). The inability of recCas9 to function on these and other recCas9 sites may be caused by important, but unknown, sequence preferences of ginβ for gix pseudo-sites. Alternatively, sub-optimal guide RNA sequences may also affect recCas9 activity at particular sites.

Programmable, recombination-based gene deletion offers advantages over current nuclease-based approaches for generating therapeutic gene knockouts. Unlike mutations induced by programmable nucleases such as ZFNs, TALENs or Cas9, recCas9 deletion is not dependent on error-prone forms of DSB repair and should not be prone to undesired chromosomal rearrangements such as translocations. Non-programmable recombinase-mediated deletions have already proven effective at removing latent HIV provirus from infected hematopoetic stem cells (23), or unwanted vector backbone resulting from *ex vivo* gene therapy (68). Furthermore, the requirement of four separate recCas9 guide RNA-programmed binding events as well as a matching dinucleotide core in the recombination substrates may reduce the likelihood of off-target modifications commonly observed in nuclease-mediated mutagenesis. Such therapeutic applications of recCas9-mediated deletions may be possible following future studies to expand the activity and substrate scope of recCas9.

Finally, recCas9 may prove especially useful for precision agricultural breeding applications. Breeding improved crops, for example, traditionally involves crossing plant varieties that may undergo genetic recombination during meiosis. Isolating desired progeny that contain novel allelic combinations while preserving known favorable traits can be difficult in traditional, random breeding experiments (69). Moreover, the existence of recombination ‘hot spots’ or ‘cold spots’ biases the chromosomal regions that participate in crossover events and thus, the amount of diversity that can be combined into progeny using unassisted breeding (69). Plant biotechnology has embraced recombinases as well as programmable nucleases such as Cas9 to enable targeted genome editing (e.g. (70)); the capabilities of recCas9 may further contribute to breeding efforts using elite cultivars by allowing researchers to manipulate the location of crossover events during meiosis. RecCas9 or future variants, in principle, could enhance or decrease the rate of recombination at specified loci, without introducing foreign DNA into the plant genome, by catalyzing favorable translocation events or removing specific mutational ‘hot spots’ that result in unfavorable crossover events.

## Supplementary Material

SUPPLEMENTARY DATA
