# Supplementary material for: A programmable Cas9-serine recombinase fusion protein that operates on DNA sequences in mammalian cells
Source: Nucleic Acids Res. 2016 Aug 11;44(20):9758–70. doi: 10.1093/nar/gkw707 (PMC5175349; doi:10.1093/nar/gkw707)
Supplement: SUPPLEMENTARY DATA [file supp_44_20_9758__index.html]

A programmable Cas9-serine recombinase fusion protein that operates on DNA sequences in mammalian cells — SUPPLEMENTARY DATA 

# A programmable Cas9-serine recombinase fusion protein that operates on DNA sequences in mammalian cells

## SUPPLEMENTARY DATA

- SUPPLEMENTARY DATA
