# Supplementary material for: A programmable Cas9-serine recombinase fusion protein that operates on DNA sequences in mammalian cells
Source: Nucleic Acids Res. 2016 Aug 11;44(20):9758–70. doi: 10.1093/nar/gkw707 (PMC5175349; doi:10.1093/nar/gkw707)
Supplement: SUPPLEMENTARY DATA [file supp_gkw707_nar-01770-f-2016-File007.pdf]

A programmable Cas9-serine recombinase fusion protein that operates on DNA sequences in mammalian cells

Brian Chaikind, Jeff L. Bessen, David B. Thompson, Johnny H. Hu, and David R. Liu

**Figure S1**	Construction of reporter plasmids
**Table S1**	Oligonucleotides for gRNA construction
**Table S2**	Oligonucleotides and gBLOCKs for reporter construction
**Table S3**	Oligonucleotides for recCas9 construction
**Table S4**	Custom sequencing oligonucleotides
**Table S5**	Genomic PCR primers
**Table S6**	List of target site sequences used in reporter assays
**Table S7**	DNA sequences encoding GGS linkers
**Table S8**	List of gRNA sequences
**Table S9**	recCas9 genomic targets identified *in silico*
**Methods for supporting material**	Methods for reporter plasmid construction, episomal isolation, and DNA sequencing
**Note S1**	DNA sequence of target sites flanking a KanR and PolyA stop sequences
**Note S2**	DNA and amino acid sequence of Ginβ-$(GGS)_8$-dCas9-GGS-FLAG-GGS-NLS
**Note S3**	Source code for recCas9 genomic target identification
**Note S4**	Limiting dilution quantification of recCas9 genomic deletion

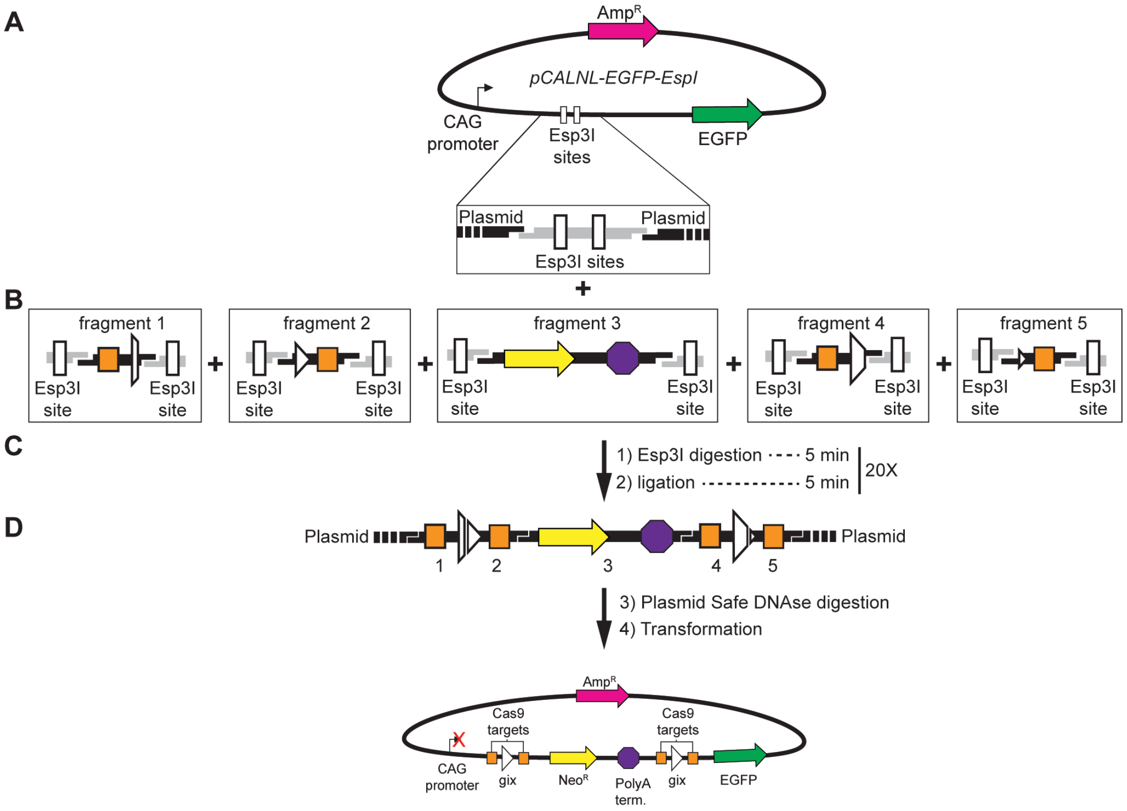

Figure S1: Reporter plasmid construction. Golden Gate assembly was used to construct the reporter plasmids described in this work. All assemblies started with a common plasmid, pCALNL-EGFP-Esp3I, that was derived from pCALNL-EGFP and contained to Esp3I restriction sites. The fragments shown are flanked by Esp3I sites. Esp3I digestion creates a series of compatible, unique 4-base pair 5' overhangs so that assembly occurs in the order shown. To assemble the target sites, Esp3I (ThermoFisher Scientific, Waltham, MA) and five fragments were added to a single reaction tube; to allow for iterative cycles of Esp3I digestion and T7 ligation. Reactions were then digested with Plasmid-Safe-ATP-dependent DNAse (Epicentre, Madison, WI) to reduce background. Colonies were analyzed by colony PCR to identify PCR products that matched the expected full length 5 part assembly product; plasmid from these colonies was then sent for sanger sequencing. For the genomic reporters shown in Figure 4, fragments 1 and 2 as well as fragments 4 and 5 were combined into two gBlocks (IDT, Coralville, IA) fragments encoding the entire target site (not shown in the figure). Assembly was then completed as described above. Details for construction can be found in the methods for the supporting material. Oligonucleotides and gBLOCKS for creation of fragments can be found in Table S3.

Table S1. Oligonucleotides for gRNA construction.

Oligonucleotide Name	Sequence
R.pHU6.TSS(-1).univ	GGTGTTTCGTCCTTTCCACAAG
F.non-target	GCACACTAGTTAGGGATAACAGTT TTAGAGCTAGAAATAGC
F.Chr10-1	GCCCATGACCCTTCTCCTCTGTTTT AGAGCTAGAAATAGC
F.Chr10-1-rev	GCTCAGGGCCTGTGATGGGAGGT TTTAGAGCTAGAAATAGC
F.Chr10-2	GGCCCATGACCCTTCTCCTCGTTT TAGAGCTAGAAATAGC
F.Chr10-2rev	GCCTCAGGGCCTGTGATGGGAGTT TTAGAGCTAGAAATAGC
F.Centromere_Chr_1_5_19-gRNA-for	GACTTGAAACACTCTTTTTCGTTTT AGAGCTAGAAATAGC
F.Centromere_Chr_1_5_19-gRNA-rev	GAGTTGAAGACACACAACACAGTT TTAGAGCTAGAAATAGC
F.Ch5_155183064-gRNA-for	GGAACTCATGTGATTAACTGGTTTT AGAGCTAGAAATAGC
F.Ch5_155183064-gRNA-rev-1	GTCTACCTCTCATGAGCCGGTGTT TTAGAGCTAGAAATAGC
F.Ch5_169395198-gRNA-for	GTTTCCCGCAGGATGTGGGATGTT TTAGAGCTAGAAATAGC
F.Ch5_169395198-gRNA-rev	GCCTGGGGATTTATGTTCTTAGTTT TAGAGCTAGAAATAGC
F.Ch12_62418577-gRNA-for	GAAATAGCACAATGAATGGAAGTT TTAGAGCTAGAAATAGC
F.Ch12_62418577-gRNA-rev	GACTTTTTGGGGGAGAGGGAGGTT TTAGAGCTAGAAATAGC
F.Ch13_102010574-gRNA-for	GGAGACTTAAGTCCAAAACCGTTT TAGAGCTAGAAATAGC
F.Ch13_102010574-gRNA-rev	GTCAGCTATGATCACTTCCCTGTTT TAGAGCTAGAAATAGC

Table S2. Oligonucleotides and gBlocks for reporter construction.

Construct Name	Sequence
1-0bp-for	TCGTCTCGGCGTCCCCAATTTTCCCAAACAGAGGTCTGTAAACCGAGGTGAGACGG
1-0bp-rev	CCGTCTCACCTCGGTTTACAGACCTCTGTTTGGGAAAATTGGGGACGCCGAGACGA
1-1bp-for	TCGTCTCGGCGTCCCCAATTTTCCCAAACAGAGGTtCTGTAAACCGAGGTGAGACGG
1-1bp-rev	CCGTCTCACCTCGGTTTACAGaACCTCTGTTTGGGAAAATTGGGGACGCCGAGACGA
1-2bp-for	TCGTCTCGGCGTCCCCAATTTTCCCAAACAGAGGTatCTGTAAACCGAGGTGAGACGG
1-2bp-rev	CCGTCTCACCTCGGTTTACAGatACCTCTGTTTGGGAAAATTGGGGACGCCGAGACGA
1-3bp-for	TCGTCTCGGCGTCCCCAATTTTCCCAAACAGAGGTaatCTGTAAACCGAGGTGAGACGG
1-3bp-rev	CCGTCTCACCTCGGTTTACAGattACCTCTGTTTGGGAAAATTGGGGACGCCGAGACGA
1-4bp-for	TCGTCTCGGCGTCCCCAATTTTCCCAAACAGAGGTaaatCTGTAAACCGAGGTGAGACGG
1-4bp-rev	CCGTCTCACCTCGGTTTACAGatttACCTCTGTTTGGGAAAATTGGGGACGCCGAGACGA
1-5bp-for	TCGTCTCGGCGTCCCCAATTTTCCCAAACAGAGGTgaaatCTGTAAACCGAGGTGAGACGG
1-5bp-rev	CCGTCTCACCTCGGTTTACAGatttcACCTCTGTTTGGGAAAATTGGGGACGCCGAGACGA
1-6bp-for	TCGTCTCGGCGTCCCCAATTTTCCCAAACAGAGGTcgaaatCTGTAAACCGAGGTGAGACGG
1-6bp-rev	CCGTCTCACCTCGGTTTACAGatttcgACCTCTGTTTGGGAAAATTGGGGACGCCGAGACGA
1-7bp-for	TCGTCTCGGCGTCCCCAATTTTCCCAAACAGAGGTtcgaaatCTGTAAACCGAGGTGAGACGG
1-7bp-rev	CCGTCTCACCTCGGTTTACAGatttcgaACCTCTGTTTGGGAAAATTGGGGACGCCGAGACGA
2-0bp-for	TCGTCTCGGAGGTTTTGGAACCTCTGTTT

	GGGAAAATTGGGGAGTCTGAGACGG
2-0bp-rev	CCGTCTCAGACTCCCCAATTTTCCCAAACAGAGGTTCCAAAACCTCCGAGACGA
2-1bp-for	TCGTCTCGGAGGTTTTGGACACCTCTGTTTGGGAAAATTGGGGAGTCTGAGACGG
2-1bp-rev	CCGTCTCAGACTCCCCAATTTTCCCAAACAGAGGTGTCCAAAACCTCCGAGACGA
2-2bp-for	TCGTCTCGGAGGTTTTGGACTACCTCTGTTTGGGAAAATTGGGGAGTCTGAGACGG
2-2bp-rev	CCGTCTCAGACTCCCCAATTTTCCCAAACAGAGGTAGTCCAAAACCTCCGAGACGA
2-3bp-for	TCGTCTCGGAGGTTTTGGACTTACCTCTGTTTGGGAAAATTGGGGAGTCTGAGACGG
2-3bp-rev	CCGTCTCAGACTCCCCAATTTTCCCAAACAGAGGTAAGTCCAAAACCTCCGAGACGA
2-4bp-for	TCGTCTCGGAGGTTTTGGACTTAACCTCTGTTTGGGAAAATTGGGGAGTCTGAGACGG
2-4bp-rev	CCGTCTCAGACTCCCCAATTTTCCCAAACAGAGGTTAAGTCCAAAACCTCCGAGACGA
2-5bp-for	TCGTCTCGGAGGTTTTGGACTTAGACCTCTGTTTGGGAAAATTGGGGAGTCTGAGACGG
2-5bp-rev	CCGTCTCAGACTCCCCAATTTTCCCAAACAGAGGTCTAAGTCCAAAACCTCCGAGACGA
2-6bp-for	TCGTCTCGGAGGTTTTGGACTTAGCACCTCTGTTTGGGAAAATTGGGGAGTCTGAGACGG
2-6bp-rev	CCGTCTCAGACTCCCCAATTTTCCCAAACAGAGGTGCTAAGTCCAAAACCTCCGAGACGA
2-7bp-for	TCGTCTCGGAGGTTTTGGACTTAGCTACCTCTGTTTGGGAAAATTGGGGAGTCTGAGACGG
2-7bp-rev	CCGTCTCAGACTCCCCAATTTTCCCAAACAGAGGTAGCTAAGTCCAAAACCTCCGAGACGA
4-0bp-for	TCGTCTCTGCACCCCCAATTTTCCCAAACAGAGGTCTGTAAACCGATGAGACGG
4-0bp-rev	CCGTCTCATCGGTTTACAGACCTCTGTTTGGGAAAATTGGGGGTGCAGAGACGA
4-1bp-for	TCGTCTCTGCACCCCCAATTTTCCCAAACAGAGGTtCTGTAAACCGATGAGACGG
4-1bp-rev	CCGTCTCATCGGTTTACAGaACCTCTGTTTGGGAAAATTGGGGGTGCAGAGACGA

4-2bp-for	TCGTCTCTGCACCCCCAATTTTCCCAAACAGAGGTatCTGTAAACCGATGAGACGG
4-2bp-rev	CCGTCTCATCGGTTTACAGatACCTCTGTTTGGGAAAATTGGGGGTGCAGAGACGA
4-3bp-for	TCGTCTCTGCACCCCCAATTTTCCCAAACAGAGGTaatCTGTAAACCGATGAGACGG
4-3bp-rev	CCGTCTCATCGGTTTACAGattACCTCTGTTTGGGAAAATTGGGGGTGCAGAGACGA
4-4bp-for	TCGTCTCTGCACCCCCAATTTTCCCAAACAGAGGTaaatCTGTAAACCGATGAGACGG
4-4bp-rev	CCGTCTCATCGGTTTACAGatttACCTCTGTTTGGGAAAATTGGGGGTGCAGAGACGA
4-5bp-for	TCGTCTCTGCACCCCCAATTTTCCCAAACAGAGGTgaaatCTGTAAACCGATGAGACGG
4-5bp-rev	CCGTCTCATCGGTTTACAGatttcACCTCTGTTTGGGAAAATTGGGGGTGCAGAGACGA
4-6bp-for	TCGTCTCTGCACCCCCAATTTTCCCAAACAGAGGTcgaaatCTGTAAACCGATGAGACGG
4-6bp-rev	CCGTCTCATCGGTTTACAGatttcgACCTCTGTTTGGGAAAATTGGGGGTGCAGAGACGA
4-7bp-for	TCGTCTCTGCACCCCCAATTTTCCCAAACAGAGGTtcgaaatCTGTAAACCGATGAGACGG
4-7bp-rev	CCGTCTCATCGGTTTACAGatttcgaACCTCTGTTTGGGAAAATTGGGGGTGCAGAGACGA
5-0bp-for	TCGTCTCGCCGAGGTTTTGGAACCTCTGTTTGGGAAAATTGGGGCTCGTGAGACGG
5-0bp-rev	CCGTCTCACGAGCCCCAATTTTCCCAAACAGAGGTTCCAAAACCTCGGCGAGACGA
5-1bp-for	TCGTCTCGCCGAGGTTTTGGACACCTCTGTTTGGGAAAATTGGGGCTCGTGAGACGG
5-1bp-rev	CCGTCTCACGAGCCCCAATTTTCCCAAACAGAGGTGTCCAAAACCTCGGCGAGACGA
5-2bp-for	TCGTCTCGCCGAGGTTTTGGACTACCTCTGTTTGGGAAAATTGGGGCTCGTGAGACGG
5-2bp-rev	CCGTCTCACGAGCCCCAATTTTCCCAAACAGAGGTAGTCCAAAACCTCGGCGAGACGA
5-3bp-for	TCGTCTCGCCGAGGTTTTGGACTTACCTCTGTTTGGGAAAATTGGGGCTCGTGAGACGG
5-3bp-rev	CCGTCTCACGAGCCCCAATTTTCCCAAACAGAGGTAAGTCCAAAACCTCGGCGAGAC

	GA
5-4bp-for	TCGTCTCGCCGAGGTTTTGGACTTAACCTCTGTTTGGGAAAATTGGGGCTCGTGAGACGG
5-4bp-rev	CCGTCTCACGAGCCCCAATTTTCCCAAACAGAGGTTAAGTCCAAAACCTCGGCGAGACGA
5-5bp-for	TCGTCTCGCCGAGGTTTTGGACTTAGACCTCTGTTTGGGAAAATTGGGGCTCGTGAGACGG
5-5bp-rev	CCGTCTCACGAGCCCCAATTTTCCCAAACAGAGGTCTAAGTCCAAAACCTCGGCGAGACGA
5-6bp-for	TCGTCTCGCCGAGGTTTTGGACTTAGCACCTCTGTTTGGGAAAATTGGGGCTCGTGAGACGG
5-6bp-rev	CCGTCTCACGAGCCCCAATTTTCCCAAACAGAGGTGCTAAGTCCAAAACCTCGGCGAGACGA
5-7bp-for	TCGTCTCGCCGAGGTTTTGGACTTAGCTACCTCTGTTTGGGAAAATTGGGGCTCGTGAGACGG
5-7bp-rev	CCGTCTCACGAGCCCCAATTTTCCCAAACAGAGGTAGCTAAGTCCAAAACCTCGGCGAGACGA
1-Chr10--54913298-54913376-for	TCGTCTCGGCGTCCCCTCCCATCACAGGCCCTGAGGTTTAAGAGAAAACCTGAGACGG
1-Chr10-54913298-54913376-rev	CCGTCTCAGGTTTTCTCTTAAACCTCAGGGCCTGTGATGGGAGGGGACGCCGAGACGA
2-Chr10--54913298-54913376-for	TCGTCTCGAACCATGGTTTTGTGGGCCAGGCCCATGACCCTTCTCCTCTGGGAGTCTGAGACGG
2-Chr10--54913298-54913376-rev	CCGTCTCAGACTCCCAGAGGAGAAGGGTCATGGGCCTGGCCCACAAAACCATGGTTCGAGACGA
4-Chr10-54913298-54913376-for	TCGTCTCTGCACCCCCTCCCATCACAGGCCCTGAGGTTTAAGAGAAAACCATTGAGACGG
4-Chr10-54913298-54913376-rev	CCGTCTCAATGGTTTTCTCTTAAACCTCAGGGCCTGTGATGGGAGGGGGTGCAGAGACGA
5-Chr10-54913298-54913376-for	TCGTCTCGCCATGGTTTTGTGGGCCAGGCCCATGACCCTTCTCCTCTGGGCTCGTGAGACGG

5-Chr10-54913298-54913376-rev	CCGTCTCACGAGCCCAGAGGAGAAGGGTCATGGGCCTGGCCCACAAAACCATGGCGAGACGA
3-for	ATCCGTCTCCAGTCGAGTCGGATTTGATCTGATCAAGAGACAG
3-rev	AACCGTCTCGGTGCGTTCGGATTTGATCCAGACATGATAAGATAC
Esp3I-insert-for	/Phos/CGCGTTGAGACGCTGCCATCCGTCTCGC
Esp3I-insert-rev	/Phos/TCGAGCGAGACGGATGGCAGCGTCTCAA
Centromere_Chr_1_5_19-1_2*	GTTGTTCGTCTCGGCGTCCTTGTGTTGTGTGTCTTCAACTCACAGAGTTAAACGATGCTTTACACAGAGTAGACTTGAAACACTCTTTTTCTGGAGTCTGAGACGGTTCTGTTTTGGTGTGATTAGTTAT
Centromere_Chr_1_5_19-4_5*	GTTGGTCGTCTCTGCACCCTTGTGTTGTGTGTCTTCAACTCACAGAGTTAAACGATGCTTTACACAGAGTAGACTTGAAACACTCTTTTTCTGGCTCGTGAGACGGTTCTGTTTTGGTGTGATTAGTTAT
Ch5_155183064-155183141-1_2*	GTTGTTCGTCTCGGCGTCCCACCGGCTCATGAGAGGTAGAGCTAAGGTCCAAACCTAGGTTTATCTGAGACCGGAACTCATGTGATTAACTGTGGAGTCTGAGACGGTTCTGTTTTGGTGTGATTAGTTAT
Ch5_155183064-155183141-4_5*	GTTGGTCGTCTCTGCACCCCACCGGCTCATGAGAGGTAGAGCTAAGGTCCAAACCTAGGTTTATCTGAGACCGGAACTCATGTGATTAACTGTGGCTCGTGAGACGGTTCTGTTTTGGTGTGATTAGTTAT
Ch5_169395198-169395274-1_2*	GTTGTTCGTCTCGGCGTCCTTAAGAACATAAATCCCCAGGAATTCACAGAAACCTTGGTTTGAGCTTTGGATTTCCCGCAGGATGTGGGATAGGAGTCTGAGACGGTTCTGTTTTGGTGTGATTAGTTAT
Ch5_169395198-169395274-4_5*	GTTGGTCGTCTCTGCACCCTTAAGAACATAAATCCCCAGGAATTCACAGAAACCTTGGTTTGAGCTTTGGATTTCCCGCAGGATGTGGGATAGGCTCGTGAGACGGTTCTGTTTTGGTGTGATTAGTTAT

Ch12_62418577-62418652-1_2*	GTTGTTCGTCTCGGCGTCCACTCCCTCTC CCCCAAAAAGTAAAGGTAGAAAACCAAGG TTTACAGGCAACAAATAGCACAATGAATG GAATGGAGTCTGAGACGGTTCTGTTTTGG TGTGATTAGTTAT
Ch12_62418577-62418652-4_5*	GTTGGTCGTCTCTGCACCCACTCCCTCTC CCCCAAAAAGTAAAGGTAGAAAACCAAGG TTTACAGGCAACAAATAGCACAATGAATG GAATGGCTCGTGAGACGGTTCTGTTTTGG TGTGATTAGTTAT
chr13_102010574-102010650-1_2*	GTTGTTCGTCTCGGCGTCCTAGGGAAGTG ATCATAGCTGAGTTTCTGGAAAAACCTAG GTTTTAAAGTTGAGGAGACTTAAGTCCAAA ACCTGGAGTCTGAGACGGTTCTGTTTTGG TGTGATTAGTTAT
chr13_102010574-102010650-4_5*	GTTGGTCGTCTCTGCACCCTAGGGAAGTG ATCATAGCTGAGTTTCTGGAAAAACCTAG GTTTTAAAGTTGAGGAGACTTAAGTCCAAA ACCTGGCTCGTGAGACGGTTCTGTTTTGG TGTGATTAGTTAT

Oligonucleotide sequences were annealed to create the fragments shown in Figure 1. The names correspond to the fragment number (1, 2, 4, or 5) and then to the number of base pair spacer nucleotides separating the Cas9 binding site from the gix pseudo site.

* Double stranded gBlocks as described in the methods within the supporting material document.

Table S3. Oligonucleotides for recCas9 construction

Oligonucleotide Name	Sequence
1GGS-link-for_BamHI	TTCATCGGATCCGATAAAAAGTATTCTATTGGTTTAGCTATCGGCAC
5GGS-link-for_BamHI	TTCATCGGATCCGGTGGTTCAGGTGGCAGCGGAG
8GGS-link-for_BamHI	TTCATCGGATCCGGAGGTCCGGAGGTAGTGGCGGCAGCGGTGGTTCAGGTGGCAGCGGAG
Cas9-rev-FLAG-NLS-AgeI	AATAACCGGTTCAGACCTTCCTTTTCTTCTTTGGGGAACCTCCCTTGTCGTCATCATCCTTATAATCGGAGCCACCGTCACCCCCAAGCTGTGACAAATC
1GGS-rev-BamHI	TGATAAGGATCCACCCTTTGGTGGTCTTCCAAACCGCC
2GGS-rev-BamH	TGATAAGGATCCACCGCTACCACCCTTTGGTGGTCTTC
Gin-for_NotI	AGATCCGCGGCCGCTAATAC
Esp3I-for-plasmid	TTGAGTcgtctcTATACTCTTCCTTTTTCAATATTATTGAAGCATTTATCAGGG
Esp3I-rev-plasmid	CTGGAAcgtctcACTGTCAGACCAAGTTTACTCATATATACTTTAGATTG
spec-Esp3I-for	GGTGTGcgtctcTACAGTTATTTGCCGACTACCTTGGTGATCTCGC
spec-Esp3I-rev	ACACCAcgtctcTGTATGAGGGAAGCGGTGATCGCC
cpec assembly-for-plasmid	CATACTCTTCCTTTTTCAATATTATTGAAGCATTTATCAGGG
cpec assembly-rev-plasmid	CTGTCAGACCAAGTTTACTCATATATACTTTAGATTG
cpec assembly-for-spec	CAATCTAAAGTATATATGAGTAAACTTGGTCTGACAGTTTGCCGACTACCTTGGTGATCTCG
cpec assembly-for-spec2	CAATCTAAAGTATATATGAGTAAACTTGGTCTGACAGTTATTTGCCGACTACCTTGGTGATCTCG
cpec assembly-rev-spec	CCCTGATAAATGCTTCAATAATATTGAAAAAGGAAGAGTATG

Table S4. Custom sequencing oligonucleotides.

Oligonucleotide Name	Sequence
Fwd CMV	CGCAAATGGGCGGTAGGCGTG
Cas9coRevE1	CCGTGATGGATTGGTGAATC
Cas9coRevE2	CCCATACGATTTCACCTGTC
Cas9coRevE3	GGGTATTTTCCACAGGATGC
Cas9coRevE4	CTTAGAAAGGCGGGTTTACG
Cas9coRevE5	CTTACTAAGCTGCAATTTGG
Cas9coRevE6	TGTATTCATCGGTTATGACAG
bGH_PArev seq1	CAGGGTCAAGGAAGGCACG
pHU6-gRNA_for	GTTCCGCGCACATTTCC
pHU6-gRNA_rev	GCGGAGCCTATGGAAAAAC
pCALNL-for1	GCCTTCTTCTTTTTCCTACAGC
pCALNL-for2	CGCATCGAGCGAGCAC

Table S5. Genomic PCR primers.

Oligonucleotide Name	Sequence
**FAM19A2-F1**	TCAAGTAGCAAAAGAAGTAGGAGTCAG
**FAM19A2-F2**	TTAGATGCATTCGTGCTTGAAG
**FAM19A2-C1**	TTAATTTCTGCTGCTAGAACTAAATCTGG
**FAM19A2-R1**	GGGAAGAAAACTGGATGGAGAATG
**FAM19A2-R2**	CATAAATGACCTAGTGGAGCTG
**FAM19A2-C2**	TGGTTATTTTGCCCATTAGTTGATGC

Table S6. List of target sites sequences used in reporter assays.

Target site name	Sequence
PAM_NT1-0bp-gix_core-0bp-NT1_PAM	CCCCAATTTTCCCAAACAGAGGTtCTGTAAACCGAGGTTTTGGAACCTCTGTTTGGGAAAATTGGGG
PAM_NT1-1bp-gix_core-1bp-NT1_PAM	CCCCAATTTTCCCAAACAGAGGTtCTGTAAACCGAGGTTTTGGcAACCTCTGTTTGGGAAAATTGGGG
PAM_NT1-2bp-gix_core-2bp-NT1_PAM	CCCCAATTTTCCCAAACAGAGGTatCTGTAAACCGAGGTTTTGGctAACCTCTGTTTGGGAAAATTGGGG
PAM_NT1-3bp-gix_core-3bp-NT1_PAM	CCCCAATTTTCCCAAACAGAGGTaatCTGTAAACCGAGGTTTTGGcttAACCTCTGTTTGGGAAAATTGGGG
PAM_NT1-4bp-gix_core-4bp-NT1_PAM	CCCCAATTTTCCCAAACAGAGGTaaatCTGTAAACCGAGGTTTTGGcttaAACCTCTGTTTGGGAAAATTGGGG
PAM_NT1-5bp-gix_core-5bp-NT1_PAM	CCCCAATTTTCCCAAACAGAGGTgaaatCTGTAAACCGAGGTTTTGGcttagAACCTCTGTTTGGGAAAATTGGGG
PAM_NT1-6bp-gix_core-6bp-NT1_PAM	CCCCAATTTTCCCAAACAGAGGTcgaaatCTGTAAACCGAGGTTTTGGcttagcAACCTCTGTTTGGGAAAATTGGGG
PAM_NT1-7bp-gix_core-7bp-NT1_PAM	CCCCAATTTTCCCAAACAGAGGTtcgaaatCTGTAAACCGAGGTTTTGGcttagctAACCTCTGTTTGGGAAAATTGGGG
PAM_NT1-6bp-gix_core-0bp-NT1_PAM	CCCCAATTTTCCCAAACAGAGGTtcgaaatCTGTAAACCGAGGTTTTGGAACCTCTGTTTGGGAAAATTGGGG
PAM_NT1-6bp-gix_core-1bp-NT1_PAM	CCCCAATTTTCCCAAACAGAGGTtcgaaatCTGTAAACCGAGGTTTTGGcAACCTCTGTTTGGGAAAATTGGGG
PAM_NT1-6bp-gix_core-2bp-NT1_PAM	CCCCAATTTTCCCAAACAGAGGTcgaaatCTGTAAACCGAGGTTTTGGctAACCTCTGTTTGGGAAAATTGGGG
PAM_NT1-6bp-gix_core-4bp-NT1_PAM	CCCCAATTTTCCCAAACAGAGGTcgaaatCTGTAAACCGAGGTTTTGGcttaAACCTCTGTTTGGGAAAATTGGGG
PAM_NT1-6bp-gix_core-5bp-NT1_PAM	CCCCAATTTTCCCAAACAGAGGTcgaaatCTGTAAACCGAGGTTTTGGcttagAACCTCTGTTTGGGAAAATTGGGG
PAM_NT1-0bp-gix_core-6bp-NT1_PAM	CCCCAATTTTCCCAAACAGAGGTCTGTAAACCGAGGTTTTGGcttagcAACCTCTGTTTGGGAAAATTGGGG
PAM_NT1-1bp-gix_core-6bp-NT1_PAM	CCCCAATTTTCCCAAACAGAGGTtCTGTAAACCGAGGTTTTGGcttagcAACCTCTGTTTGGGAAAATTGGGG
PAM_NT1-2bp-gix_core-	CCCCAATTTTCCCAAACAGAGGTatCTGTAAACCG

6bp-NT1_PAM	*AGGTTTTGG*cttagcAACCTCTGTTTGGGAAAATTG**GGG**
PAM_NT1-3bp-gix_core-6bp-NT1_PAM	**CCC**CAATTTTCCCAAACAGAGGTaat*CTGTAAACCGAGGTTTTGG*cttagcAACCTCTGTTTGGGAAAATTG**GGG**
PAM_NT1-4bp-gix_core-6bp-NT1_PAM	**CCC**CAATTTTCCCAAACAGAGGTaaat*CTGTAAACCGAGGTTTTGG*cttagcAACCTCTGTTTGGGAAAATTG**GGG**
PAM_NT1-5bp-gix_core-6bp-NT1_PAM	**CCC**CAATTTTCCCAAACAGAGGTgaaat*CTGTAAACCGAGGTTTTGG*cttagcAACCTCTGTTTGGGAAAATTG**GGG**
Chromosome_10-54913298-54913376*	**CCC**CTCCCATCACAGGCCCTGAGgtttaa*GAGAAAACCATGGTTTTGTG*ggccagGCCCATGACCCTTCTCCTCT**GGG**
Centromere_Chromosomes_1_5_19	**CCT**TGTGTTGTGTGTCTTCAACTcacag*AGTTAAACGATGCTTTACAC*agagtaGACTTGAAACACTCTTTTTC**TGG**
Chromosome_5_155183064-155183141 (site 1)	**CCA**CCGGCTCATGAGAGGTAGAGctaag*GTCCAAACCTAGGTTTATCT*gagaccGGAACTCATGTGATTAACTG**TGG**
Chromosome_5_169395198-169395274 (site 2)	**CCT**TAAGAACATAAATCCCCAGGaattc*ACAGAAACCTTGGTTTGAGC*tttggaTTTCCCGCAGGATGTGGGAT**AGG**
Chromosome_12_62418577-62418652	**CCA**CTCCCTCTCCCCCAAAAAGTaaagg*TAGAAAACCAAGGTTTACAG*gcaacAAATAGCACAATGAATGGAA**TGG**
Chromosome_13_102010574-102010650 (FGF14)	**CCT**AGGGAAGTGATCATAGCTGAgtttct*GGAAAAACCTAGGTTTTAAA*gttgaGGAGACTTAAGTCCAAAACC**TGG**

Protoadjacent spacer motifs (PAMs) are in bold. Base pair spacers are lower case. Gix site or pseudo sites are in italics and dCas9 binding sites are underlined.

* Chromosome_10 reporter contains two overlapping PAM sites and dCas9 binding sites on the 5’ and 3’ ends of the gix sites.

Table S7. DNA sequences encoding GGS linkers.

GGS linkers	DNA sequences for GGS linkers
2XGGS	GGTGGTAGCGGTGGATCC
5XGGS	GGTGGATCCGGTGGTTCAGGTGG CAGCGGAGGGTCAGGAGGCTCT
8XGGS	GGTGGATCCGGAGGGTCCGGAGG TAGTGGCGGCAGCGGTGGTTCAG GTGGCAGCGGAGGGTCAGGAGGC TCT

Table S8. List of gRNA sequences.

gRNA name	gRNA-sequence
on-target_gRNA	ACCTCTGTTTGGGAAAATTG
non-target_gRNA	gCACACTAGTTAGGGATAACA
Chromosome_10-54913298-54913376_gRNA-rev-5	gCCTCAGGGCCTGTGATGGGA
Chromosome_10-54913298-54913376_gRNA-rev-6	gCTCAGGGCCTGTGATGGGAG
Chromosome_10-54913298-54913376_gRNA-for-5	GGCCCATGACCCTTCTCCTC
Chromosome_10-54913298-54913376_gRNA-for-6	GCCCATGACCCTTCTCCTCT
Centromere_Chromosomes_1_5_19-gRNA-for	GACTTGAAACACTCTTTTTC
Centromere_Chromosomes_1_5_19-gRNA-rev	gAGTTGAAGACACACAACACA
Chromosome_5_155183064-155183141_(site 1)_gRNA-for	GGAACTCATGTGATTAACTG
Chromosome_5_155183064-155183141_(site 1)_gRNA-rev	gTCTACCTCTCATGAGCCGGT
Chromosome_5_169395198-169395274_(site 2)_gRNA-for	gTTTCCCGCAGGATGTGGGAT
Chromosome_5_169395198-169395274_(site 2)_gRNA-rev	gCCTGGGGATTTATGTTCTTA
Chromosome_12_62418577-62418652_gRNA-for	gAAATAGCACAATGAATGGAA
Chromosome_12_62418577-62418652_gRNA-rev	gACTTTTTGGGGGAGAGGGAG
Chromosome_13_102010574-102010650_(FGF14)_gRNA-for	GGAGACTTAAGTCCAAAACC
Chromosome_13_102010574-102010650_(FGF14)_gRNA-rev	gTCAGCTATGATCACTTCCCT
**Off target-for (CLTA)**	GCAGATGTAGTGTTTCCACA
**Off target-rev(VEGF)**	GGGTGGGGGGAGTTTGCTCC
**Chrom**osome_12_**62098359-62098434_(FAM19A2)_gRNA-rev**	gATATCCGTTTATCAGTGTCA
Chromosome_12_**62098359-62098434_(FAM19A2)_gRNA-for**	gTTCCTAAGCTTGGGCTGCAG
Chromosome_12_**62112591-62112668_(FAM19A2)_gRNA-rev**	gCCTAAAAGTGACTGGGAGAA
Chromosome_12_**62112591-62112668_(FAM19A2)_gRNA-for**	gCACAGTCCCATATTTCTTGG

Table S9. recCas9 genomic targets identified *in silico*

Chr.	Start	End	Sequence	Pattern ID
chr1	34169027	34169103	CCTTTAGTGAAAAGTAGACAGCTCTGAATATGAAAGGTAGGTTTTCATTTCTGGGAAAGAGACGCCAAGTGATGTGG	2
chr1	51006703	51006780	CCTCCAATAAATATGGGACTATGTGGAAAGACCAAACCTACGTTTGATTGGTGTACCTGAAAGTGACGGGAAGAATGG	1
chr1	89229373	89229450	CCATTCTGCCCGTCACTTTCAGGTACACCAATCAAACGTAGGTTTAGTCTTTTCACATAGTCCCATATTTCTTGGAGG	1
chr1	115638077	115638154	CCATTCTCCCCGTCACTTTCAGGTACAACAATCAAACGTAGGTTTGGTCTTTTCACATAGTCCCATATTTCTTGGAGG	1
chr1	122552402	122552478	CCTTGTAGTGTGTGTATTCAACTCACAGAGTTAAACGATCCTTTACACAGAGCAGACTTGAAACACTCTTGTTGTGG	2
chr1	122609874	122609950	CCTTGTAGTGTGTGTATTCAACTCACAGAGTTAAACGATCCTTTACACAGAGCATACTTGAAACACTCTTTTTGTGG	2
chr1	122668677	122668753	CCTTGTGTTGTGTGTATTCAACTCACAGAGTTAAACGATCCTTTACACAGAGCAGACTTGAAACACTCTTTTTGTGG	2
chr1	123422419	123422495	CCTTGTGTTGTGTTTATTCAACTCACAGAGTTAAACGATCCTTTACACAGAGCAGACTTGAAATACTCTTTTTGTGG	2
chr1	123648614	123648690	CCTTGTAGTGTGTGTATTCAACTCACAGAGTTAAACGATCCTTTACACAGAGCATACTTGAAACACTCTTTTTGTGG	2
chr1	123806335	123806411	CCTTGTATTGTGAGTATTCAACTCACAGAGTTAAACGATCCTTTACACAGAGCAGACTTGAAACACTCTTTTTGTGG	2
chr1	124078228	124078304	CCTTGTGTTGTGTGTCTTCAACTCACAGAGTTAAACGATGCTTTACACAGAGTAGACTTGAAACACTCTTTTTCTGG	2
chr1	124231074	124231150	CCTTGTGTTGTGTGTATTCAACTCACAGAGTTAAACGATCCTTTACACAGAGCAGACTTGTAACACTCTTTTTGTGG	2
chr1	124232435	124232511	CCTTGTGTTGTGTGTATTCAACTCACAGAGTTAAACGATCCTTTACACAGAGCAGACGTGAAACACTCTTTTTGTGG	2
chr1	124344781	124344857	CCTTGTGTTGTGTGTATTCAACTCACAGAGTTAAACGATCCTTTACACAGAGCAGACTTGAAACACTCTTTTTGTGG	2
chr1	124435716	124435792	CCTTGTGTTGTGTGTATTCAACTCACAGAGTTAAACGATCCTTTACACAGAGGAGACTTGTAACACTCTTTTTGTGG	2
chr1	158677186	158677262	CCTGAGGTTTTCCAGGTTTTAAAAGGAAACCTAAAGGTAGGTTTAGCATTAAGTGTCTTGAAGTTTATTTTAAAAGG	2
chr1	167629479	167629554	CCAAAATTCCCACAAAACCGAATGCATCAGTCAAAGCAAGGTTTGAAGAAAAGATTTACCACTTCAGGGAGCTTGG	4
chr1	167783428	167783504	CCTTTTCTGGATATCGTTGATGCTCTGTATGCAAAAGGTAGGTTTTTGGGTTATGTTGTTAAACAGTGATTGAATGG	3
chr1	169409367	169409444	CCTCCAAGAAATATGGAACTATGTGAAAAGACCAAACCTACGTTTGATTGGTGTACCTGAAAGTGACAGAGAGAATGG	1
chr1	174145346	174145423	CCTCCAAGAAATATGGGACTATGTGAGAAGACCAAACCTACGTTTGATTGGTGTACCTGAAAGTGATGGGGAGAATGG	1
chr1	183750168	183750245	CCATTCTCCCCATCGCTTTCAGGTACACCAATCAAACGTAGGTTTGGTCTTTTCACATAGTTCCATATTCTTTGGAGG	1
chr1	200801540	200801617	CCATTCTCCCCATCACTTTCAGGTGTACCGATCAAACGTAGGTTTGGTCTTTTCACATAGTCCCATATTTCTTGGAGG	1
chr1	207589936	207590013	CCTCCAAGAAATATGGGACTATGTGAAAAGACCAAACCTACGTTTGATTGGTGTACCTGAAAGTGACGGGGAGAATGG	1
chr1	209768370	209768445	CCTTCAGGGCAGAAACAGCTCTACTAGCAGAGAAAGCAAGCTTTCAATATTGTGCAATACAAAAACGAGAGCAGGG	4
chr1	218652378	218652455	CCATTCTCCTCATCTCCTTCTGGTACTCCAATCAAACGTAGGTTTGGTCTTTTCTCATAGTCTCATATTTCTTGGAGG	1
chr1	222147250	222147327	CCTCCAAGACATATAGGACTATGTGAAAATACCAAACCTACGTTTGATTGGTGTACCTGAAAGTGACAGGGAGTATGG	1
chr1	245870710	245870785	CCTGCCAGATACCAGTAGTCACTGTGAATTACAAAGCTACGTTTCTTCCATAGGGAAAGTTTGGAGTCCAGCCAGG	4
chr2	2376037	2376114	CCATTCTCCCTGTCACTTTCAGGTACACCAATCAAACGTAGGTTTGGTCTTTTCACATAGTCCCATATTTCTTGGAGG	1

chr2	4119629	4119706	CCATTCTCCCCACCACTTTCAGGTACACCAATCAAACGTAGGTT TGGTCTTTTCACATAGTCCCATATTTCTTGTAGG	1
chr2	4909047	4909124	CCTAACCAGAAACTAACTAATAGATATGGCAGAAAGCATCCTT TCACTTTTGTTCTGGGAGAGGGAAGAAGCAAAGG	1
chr2	28984877	28984953	CCATTTTGGGGAGGCCTTGATGGGAAGCTGGAAAAGGAAGCTTT CCTCCCAGTCCTGCTGAAGGCCTTGCCAGCTGG	2
chr2	31755833	31755910	CCTCCAAGAAACACAGGACTATGTGAAAAGATCAAACCTACGTT TGATTGGTGTTCCTGAAAGTGATGGGGAGAATGG	1
chr2	39829583	39829660	CCATTCTCTTCATGACTTTCAGGTACACCATTGAAACGTAGGTT TGGTCTTTTCACATTGTCCCATATTTCTTGGAGG	1
chr2	60205947	60206024	CCATTCTCCCCATCACTTTCAGGTACACCAATCAAACGTAGGTT TGGTCTTTTCACATAGTCCCGTATTTCTTGGTGG	1
chr2	79082362	79082439	CCATTCTCCCTGTCACTTTCAGGTACACCAATCAAACGTAGGTT TGGTCTTTTCACATAGTCCCATATTTCTTGGGGG	1
chr2	79082362	79082438	CCATTCTCCCTGTCACTTTCAGGTACACCAATCAAACGTAGGTT TGGTCTTTTCACATAGTCCCATATTTCTTGGGG	3
chr2	108430915	108430992	CCTCCAAGAAATATGAGATTATATGAAAAGACCAAACCTACGTT TGATTGGTGTACTTTAAAGTGACGGGGAGAATGG	1
chr2	115893685	115893762	CCATTCTCCCCGTCATTTTCAGGTACACCAATCAAACGTAGGTT TGGTCTTTTCACATAGTCCCAAATTTCTTGGAGG	1
chr2	119620068	119620145	CCCCCAAGAAATGTGGGACTATATGAAAAGACCAAACCTACGTT TGACTGGTGTACCTAAAAGTGATGGGGAGAATGG	1
chr2	119620069	119620145	CCCCAAGAAATGTGGGACTATATGAAAAGACCAAACCTACGTTT GACTGGTGTACCTAAAAGTGATGGGGAGAATGG	2
chr2	128495068	128495144	CCCATTGGTGCTGACCAGATGGTGAAGGAGGCAAAGGTTGCTTT GAATGACTGTGCTCTGGGGTGAGCCAGGCCTGG	2
chr2	133133559	133133634	CCCTTTACAGAGGTGAGCTTTGTTATTAGTAAAAAGGTAGGTTT CCCTGTTTTTCTGAAGAAAAGCTGTGAGTGGG	4
chr2	134174983	134175060	CCACTGCCCATTGACAGAGTGGCGAGGTGGGTGAAACCTTGCTT TCCTCCTGGCCCATGGGCAGGGTGGGGCTGTGGG	1
chr2	134174983	134175059	CCACTGCCCATTGACAGAGTGGCGAGGTGGGTGAAACCTTGCTT TCCTCCTGGCCCATGGGCAGGGTGGGGCTGTGG	3
chr2	138069945	138070022	CCATTCTCCCTGTCACTTTTAGATACACCAATCAAACGTAGGTT TGGTCTTTTCACATAGTCCCATGTTTCTTGGAGG	1
chr2	138797420	138797496	CCTCCAAGAAATATCAACTGTGTGAAAAGACGAAACCTACGTTT GATTAATGTACCTGAAAGTGACAGGGAGAATGG	2
chr2	145212434	145212511	CCATTCTCCCATTAACTTTCAAGTACACCAATCAAAGGTAGGTT TGGTGTTTTCCCATAGTCCCGTATTTCTTGGAGG	1
chr2	147837842	147837919	CCTTTTCATCATGCCCCTTTCACTTTAAGGTGAAAACCTTGCTT TACATGTCAGAGAAAAGAAGAGCCCTCAGCTGGG	1
chr2	147837842	147837918	CCTTTTCATCATGCCCCTTTCACTTTAAGGTGAAAACCTTGCTT TACATGTCAGAGAAAAGAAGAGCCCTCAGCTGG	3
chr2	154152540	154152617	CCATTCACCCCGTCACTTTCAGGTACACCAATCAAACGTAGGTT TGGTCTTTTCACATAGTCCCATATTTCTTGGAGG	1
chr2	157705943	157706019	CCTCCAAGAAATATGGGACTATGTGAAAAGACCAAACCTACGTT TGATGGTGTACCCGAAAGTGACAGGGAGAATGG	3
chr2	158361152	158361229	CCACCAAGAAATATGGGACTATGTGAAAAGACCAAACCTACGTT TGATAGGTATACCTGAAAGTGACAGGGAGAATGG	1
chr2	161461006	161461083	CCATTCTCCCCATCACTTTCAGGTGCACCAATCAAACGTAGGTT TGGTCTTTTCACATAGTCCCATATTTCTTGGAGG	1
chr2	179077376	179077453	CCCTCAAGAAATATGAGACTATGTGAAAAGACCAAACCTACGTT TGACTGGTATACCTGAAAGTGACAGGGAGAATGG	1
chr2	179077377	179077453	CCTCAAGAAATATGAGACTATGTGAAAAGACCAAACCTACGTTT GACTGGTATACCTGAAAGTGACAGGGAGAATGG	2
chr2	181090699	181090776	CCTCCAACAAATATGGGACTATGTGAAAAGACCAAACCTACGTT TGATTGGTGTACCTGAAAGTGACGGGGATAATGG	1
chr2	182331957	182332034	CCATTCTCTCCCTCACTTTCAAGTACACCAATCAAACGTAGGTT TGGTCTTTTCACATAGTCTTATATTTCTTGGCGG	1
chr2	183620562	183620638	CCATTCTCCCTGTCACTGTCAGTACACCAATCAAACGTAGGTTT GGTCTCTTCACATAGTCCCATATTTCTTGGAGG	2
chr2	207345927	207346003	CCTCCAAGAAATATGGGACTATGTGAACAGACCAAACCTACGTT	3

			TGATTGGTGTACCTGAAAGTGATGGCAGAATGG	
chr2	216652047	216652123	CCACCATGCCTGGCCACCACACATTTTTTTCTAAAGCTTGGTTTTGGCCACAGTGAGAGTTTCTTGGGCTGTCAGGG	2
chr2	216652047	216652122	CCACCATGCCTGGCCACCACACATTTTTTTCTAAAGCTTGGTTTTGGCCACAGTGAGAGTTTCTTGGGCTGTCAGG	4
chr2	223780040	223780116	CCCACTAGGTGGCGATATCTGAGGGTCCAATGAAACCATGCTTTTTACTCAGATCTTCCACTAACCACCTCCCCCGG	2
chr2	224486595	224486672	CCTCTAAGAAATATGGGACTATGTGAAAAGACCAAACCTACGTTTGACTGGTGTACCTGAAAGTGACGGGGAGAATGG	1
chr2	230526902	230526979	CCTCCAAGAAATATGGGACTATGTGAAAAGACCAAACCTACGTTTGATTAGTGTACCTGAAAGTGACGGGGAGAATGG	1
chr2	232036127	232036204	CCATTCTCCCTGTCACTTTCAGGTACATCAATCAAACGTAGGTTTGGTCTTTTCACATAGTCCCATATTTCTTGGAGG	1
chr3	4072812	4072889	CCTCCAAGAAATATGGGACTATGTGAAAAGACCAAACCTACGTTTGACTGGTGTACCTGAAAGGGATGGGGAGAATGG	1
chr3	9261677	9261754	CCCCCAAGAAATATGAGACTATGTGAAAAGACCAAACCTACGTTTGATTGGTGTACCTGAAAGTGACAGGGAGAATGG	1
chr3	9261678	9261754	CCCCAAGAAATATGAGACTATGTGAAAAGACCAAACCTACGTTTGATTGGTGTACCTGAAAGTGACAGGGAGAATGG	2
chr3	16732146	16732223	CCTCTAAGAAATATGGGACTATGTGAAAAGACCAAACCTACGTTTGATTGGTGTAACTGAAAGTGACAGGGAGAATGG	1
chr3	17450712	17450789	CCTCCAAGAAATATGCGCCTATGTGAAAAGACCAAACCTACGTTTGATTGGTATACCTGAAAGTGATGGAGAGAATGG	1
chr3	21559769	21559846	CCATTCTCCCTGTCACTTTGAGGTACACCAATCAAACGTAGGTTTGGTCTTTTCACATATTCGCATATTTCTTGGAGG	1
chr3	23416658	23416735	CCATTCTCCCCGTCACTTTCAGGTACACCAACCAAACGTTGGTTTGGTCTTTTCACATAGTCCCATATTTCTTGGAGG	1
chr3	29984019	29984096	CCATTCTCCCTGTCACTTTCCAGTACACCAGTCAAACGTAGGTTTGGTCTTTTCACATACTCCCATATTTCTTGGAGG	1
chr3	38269551	38269627	CCTGGCCTAATTTTTAATTCTTAGTTTGACTTAAACCTTGCTTTTAGTGTGATGGCGACAAAAGCTGAGCTGAAAGG	2
chr3	40515213	40515288	CCAGTGCTTTTTGGTTTTAAAGGCAAGCCTCCAAACCTTCCTTTCTCCTGGATGCTGTGGTGGTTGCCATGCATGG	4
chr3	49233612	49233687	CCCAACTCCTGCGAGAAGTAGCTCACCATGACAAAGCTACCTTTGCTTTTATCGTTTTGCAAAACAAAAAAGGGGG	4
chr3	66292894	66292971	CCATTCTCCCCGTCACTTTGAGGTGTGCCAATCAAACGTAGGTTTGGTCTTTTCACATAGTCCTATATTTCTTGGAGG	1
chr3	67541493	67541570	CCTCCAAAAAAATATGGGACTACGTAAAAAGACCAAACCTACGTTTGATTGGTGTACCTGAAACTGACAGGGAGAATGG	1
chr3	82273011	82273088	CCATTCTCCCCGTCACTTTCAGGTACACCAATCAAACGTAGGTTTGGTCTTTTCACATAGTTCCATATTTCTTGGAGG	1
chr3	98683349	98683426	CCTACAAGATATATGGGACTATGTGAAAAGACCAAACCTACGTTTTACTGGTGTGCCTGAAACTGACGGGGAGAATGG	1
chr3	101923653	101923730	CCATTCTCTCTGTCACTTTCAGGTACACCAATCAAACGTAGGTTTGGTCTTTTCACATAGTCCCATATTTCTTGGAGG	1
chr3	114533467	114533544	CCTCCAAGAAATATGGGACTATGTGAAAAGACCAAACCTACGTTTCATTGGTGTACCTGAAAGTGATAGGGAGAATGG	1
chr3	132607602	132607679	CCTCCAAAAAATATGGGATGATGTGAAAAGACCAAACCTAGGTTTGACTGGTGTACCTGAAAATGATGGGGAGAATGG	1
chr3	137545176	137545253	CCTCCAAGAAATATGAGACTATGTGAAAAGACCAAACCTACGTTTGATTGGTGTACCTGAAAGTGACAGGGAGAATGG	1
chr3	137655679	137655756	CCTCCAAGAAATATGGGACTACGTGAAAAGATCAAACCTACGTTTGATTGTTGTACCTGAAAGTGATGGGGAGAATGG	1
chr3	137662040	137662117	CCTCCAAGAAATATGGGACTATGTGAAAAGACCAAACCTACGTTTGATTGTTGTACCTGAAAGTGATGGGGAGAATGG	1
chr3	142133796	142133873	CCTCAAAAGTGTTCTGGTTTTGTTTTGTTTTTTAAACCATGGTTTTACCTCTGGCTTAGTGGGACTAAAAATAGGAGG	1
chr3	146726949	146727026	CCTCCAAGAAATATGGGACTATGTGAAAAGACCAAACCTACGTTTGACTGGTGTACCTGAAAGTGATGGGGAAAATGG	1
chr3	152421096	152421173	CCTCCAAGAAATATGGGACTGTGTGTAAAGACCAAACCTACGTTTGATTGGTGTACCTCAAAGTGATGGGGAGAATGG	1

chr3	170620247	170620324	CCATTCTCCCCATCACATTCAGGTACACCAATCAAACGTAGGTTTGGTCTTTTCACATAGTCCCATATTTCTTGGAGG	1
chr3	181166873	181166949	CCCCTGGAAAAGTTGGAGCATCACAGGAAAAGCAAACCAACCTTTTTTCTCCCCTAGGTAAACTGGGGAGCCAGGGG	3
chr3	181166874	181166949	CCCTGGAAAAGTTGGAGCATCACAGGAAAAGCAAACCAACCTTTTTTCTCCCCTAGGTAAACTGGGGAGCCAGGGG	4
chr4	6604233	6604309	CCTTCCCCAGTTGCAGCAGACAAGAGTCTCGAAAAGCTTGCTTTGGTTGCTGCAGTGGATGGGTTGGTAGGCACAGG	2
chr4	6626269	6626344	CCCCCACCTCCCAAGCTGCTGGCTTCTCGAATAAAGCTACCTTTCCTTTTACCAAAACTTGTCTCTCGAATGTCGG	4
chr4	8155396	8155472	CCTTGGCCCTGGACAGCTGCTTTTCCTTCCCTAAACCTTGGTTTCCCCCTTTGTGCAGGTGGGTGGGTTTGGGCTGG	2
chr4	10386803	10386880	CCTCTTCTAGTGAACCCATGGGGTTACCAAGGGAAAGCAACCTTTTGATAAATATTCCCATCTTTTTATGTTGTCTGG	1
chr4	20701579	20701656	CCACTTGAAAGGGTTACCAAGGATAAGATTTTTAAAGCTTGCTTTCACAAACAACTCATGCTCCAGGCTTGTCAGTGG	1
chr4	29594286	29594363	CCTTTCTCCCCATCACTTTCAGGTACACCAATCAAACGTAGGTTTGATCTTTTCACATAGTCCCATATTTCTTGGAGG	1
chr4	53668422	53668499	CCATTCTCCCCATCAATTTCAGTTACACCAATGAAACGTAGGTTTGGCCTTTTCACATAGTCCCATATTTCTTAGAGG	1
chr4	74914802	74914879	CCATTCTCCCTGTCACTCTCAGGTACACCAATCAAACGTAGGTTTGGTCTTTTCATATAGTCCCATATTTCTTGGAGG	1
chr4	75332783	75332859	CCTCCAAGAAAATTGGGACTATGTGAAAAAACCAAACCTACGTTTGATTGATGTACCTGAAAGTGACAGGAGAATGG	3
chr4	88123643	88123720	CCTTCAAGAAATATGGGACTATGTGAAAGGACAAAACCTACGTTTTATTGGTGTACCTGAAAGTGACAGGGAGAATGG	1
chr4	89567192	89567269	CCATTCTCCCCATCACTTTCAGGTACGCTAATCAAACGTAGGTTTGATCTTTTCACATAGTCTTATATTTCTTGGAGG	1
chr4	93556577	93556654	CCTCCAAGAAATATGGGACTATGTGAAAAGACCAAACCTACGTTTGACTGGTGTACCTCAATGTGACAGGGAGAATGG	1
chr4	100266379	100266456	CCATTCTCCCTGTCACTTTTAGGTACACCAATCAAACGTACGTTTGGTCTTTTCACATAGACCCATATTTCTTGGAGG	1
chr4	103486234	103486311	CCTTCAAGAAATATGGGACTGTGTGAAAAGACCAAAGCTAGGTTTGATTGGTGTACCTGAAAGTGATGGGGAGAATGG	1
chr4	105923129	105923204	CCTACTATTCACAGAGTAATGCAGTTTGCTGAAAAGGTTGGTTTTTGCTGACCTCTGAGAGCTCACATTACAGTGG	4
chr4	106874711	106874788	CCATTCTCTCTGTCACTTTCTGGTACACCAATCAAACGTAGGTTTGCTCTTTTCACATAATCCCATATTTATTGAAGG	1
chr4	115805791	115805867	CCATAACATGTATTTGCTGGTGCTAGACTCTCCAAAGCTAGGTTTCTTTCTACAACAATGGCTGGAAGTCTTCTTGG	3
chr4	122033277	122033354	CCATTCTCCCCATCACTTTCAGGTACACCAATCAAACGTAGGTTTGGTCTTCTCACACAGTCCCATATTTCTTGGAGG	1
chr4	129125132	129125209	CCATTCTTCCCATTACTTTCAGGTACACCAATCAAACGTAGGTTTGGTCTTTTCACATAGTCCCACATTTCTTGGAGG	1
chr4	135472562	135472639	CCATTCTCCCCCTCACTTTCAGGTACACCAATCAAACGTAGGTTTGGTCTTTTCACATTGTCCCATATTTCTTGGAGG	1
chr4	138507099	138507176	CCATTCTCCCCAGCACTTACAGGTACACCAATCAAACGTAGGTTTGGTCATTTCACATAGTCCCATATTTCTTGGAGG	1
chr4	144249093	144249170	CCATTCTCCCTGTCACTTTCAGGTACAGCAATCAAACGTAGGTTTGGTCTTTTCACATGGTCCCATATTTCTTGGAGG	1
chr4	144436406	144436483	CCTCCAAGAAATATGAGACTATGTGAAAAGACCAAACCTACGTTTGATTGGTGTACCTGAAAGTGACGGGGAAGATGG	1
chr4	154110259	154110336	CCTCCAAGAAATATGAGACTATGTGAAAAGACCAAACCTACGTTTGATTGGTGTACCTGAAAGTGACAGGGAGAATGG	1
chr4	154893438	154893515	CCTCCAAGAGATATGAGACTATGTAAATAGACCAAACCTACCTTTGATTGGTGTACGTGAAAGTGACAGGAAGAATGG	1
chr4	161116854	161116931	CCATTCTCCCCATCACTTTCAGGTACACCAACCAAACGTAGGTTTGGTCTTTTCACATAGTCTCATATTTCTTGGAGG	1
chr4	165140748	165140823	CCTCCATTGACTACTCCTTATCATTGGCTAGAAAACCTACCTTTCAACCAGTTTCTAAGGCCAAGAAACTTGGAGG	4
chr4	181928508	181928585	CCACCAAGAAATATGGGACTACGTGAAAAGACCAAACCTACGTT	1

			TGATGGGTGTGCCTGAAAGTGACGGGAAGAATGG	
chr4	187521958	187522035	CCTCCAAGAAATAAGGGACTATGTGAAAAGACCAAACCTACGTT TGATTGGTGTACCTGAAGGTGACAGGGAGAATGG	1
chr5	12675639	12675715	CCAAAGGGCCTTTGTGATTCTACTTTGTAATATAAAGGATGGTT TCTTACTACGGTTGGTGTCCTTGCAGGAGTGGG	3
chr5	29271804	29271881	CCTCCAAGAAATATGGGACTATGTGAAAAGACCAAACCTACGTT TGATTGGTGTACCTGAAAGTGATGGGGAGAATGG	1
chr5	35352660	35352737	CCATTCTCCCCGTTACTTTCAGGTACACCAATAAAACCTAGGTT TGGTCTTTTCACATAGTCCCATATTTCTTGGAGG	1
chr5	38723235	38723310	CCCATATCTCTGGCAAGGGCAGCTCTCTGGCTAAACCAAGCTTT CCTGTAGAGCTTGAGTTCCAAGGCAGCGTTGG	4
chr5	47358339	47358415	CCTTGTAGTGTGTGTATTCAACTCACAGAGTTAAACGATCCTTT ACACAGAGCAGACTTGAAACACTCTTGTTGTGG	2
chr5	47415811	47415887	CCTTGTAGTGTGTGTATTCAACTCACAGAGTTAAACGATCCTTT ACACAGAGCATACTTGAAACACTCTTTTTGTGG	2
chr5	47474614	47474690	CCTTGTGTTGTGTGTATTCAACTCACAGAGTTAAACGATCCTTT ACACAGAGCAGACTTGAAACACTCTTTTTGTGG	2
chr5	48228356	48228432	CCTTGTGTTGTGTTTATTCAACTCACAGAGTTAAACGATCCTTT ACACAGAGCAGACTTGAAATACTCTTTTTGTGG	2
chr5	48454551	48454627	CCTTGTAGTGTGTGTATTCAACTCACAGAGTTAAACGATCCTTT ACACAGAGCATACTTGAAACACTCTTTTTGTGG	2
chr5	48612272	48612348	CCTTGTATTGTGAGTATTCAACTCACAGAGTTAAACGATCCTTT ACACAGAGCAGACTTGAAACACTCTTTTTGTGG	2
chr5	48884165	48884241	CCTTGTGTTGTGTGTCTTCAACTCACAGAGTTAAACGATGCTTT ACACAGAGTAGACTTGAAACACTCTTTTTCTGG	2
chr5	49037011	49037087	CCTTGTGTTGTGTGTATTCAACTCACAGAGTTAAACGATCCTTT ACACAGAGCAGACTTGTAACACTCTTTTTGTGG	2
chr5	49038372	49038448	CCTTGTGTTGTGTGTATTCAACTCACAGAGTTAAACGATCCTTT ACACAGAGCAGACGTGAAACACTCTTTTTGTGG	2
chr5	49150718	49150794	CCTTGTGTTGTGTGTATTCAACTCACAGAGTTAAACGATCCTTT ACACAGAGCAGACTTGAAACACTCTTTTTGTGG	2
chr5	49241653	49241729	CCTTGTGTTGTGTGTATTCAACTCACAGAGTTAAACGATCCTTT ACACAGAGGAGACTTGTAACACTCTTTTTGTGG	2
chr5	88582714	88582790	CCTTTTCATAAGAAGAAAATCGACTCATCATTGAAACCAAGCTT TGGTACAATTTCATTGATGTTTCCAGAAGCAGG	3
chr5	93497156	93497231	CCCATAGACTATGATAGAAACAAAATAACCCAAAAGCTAGCTTT CTGATTGAGTTTCCATAAATGCAATGTGAAGG	4
chr5	94295029	94295105	CCATTCACTTGTCACTTTCTGGTACACCAATCAAACGTAGGTTT GGTCTTTTCACATAGTCTCATATTTCTTGGAGG	2
chr5	94956746	94956823	CCTCCAAGAAATATGGGACTCTGTAAAGAGACCAAACCTACGTT TGATTGGTGTACCTGAAAGTGAAGGGGAGAATGG	1
chr5	106003488	106003565	CCATTCTCCCCGTCATTTTCAGGTACACCAATCAAACCTAGGTT TGGTCTTTTTACATAGTCCCATATTTCTTGGAGG	1
chr5	118727905	118727982	CCTCCACGAAACATGGGACTATGTGAAAAGACCAAACCTACGTT TGATTGGTGTACCTGAAAGTGACAGGGAGAATGG	1
chr5	132156032	132156109	CCAATTTCCCCCTCACTTTCAGATACACCAATCAAACGTAGGTT TGGTCTTTTCACATAGTTCCATATTTCCTGGAGG	1
chr5	152037951	152038028	CCATTCTCCCCATCACTTTCAGGTACACCAATCAAACGTAGGTT TGGTCTTTTCACATATTCCCATATGTCTTGGAGG	1
chr5	155183064	155183141	CCCACCGGCTCATGAGAGGTAGAGCTAAGGTCCAAACCTAGGTT TATCTGAGACCGGAACTCATGTGATTAACTGTGG	1
chr5	155183065	155183141	CCACCGGCTCATGAGAGGTAGAGCTAAGGTCCAAACCTAGGTTT ATCTGAGACCGGAACTCATGTGATTAACTGTGG	2
chr5	163148211	163148288	CCTTCAAGAAATATGGGACTATGTGAAGAGACCAAACCTACGTT TGATTGGTGTAGCCAAAAGTGATGGGGAAAATGG	1
chr5	165889537	165889614	CCTCAGATTAGATTTACTTGCAAAGAGACATTTAAAGGATCGTT TTGATACTATTTTGAAAGTACTATACAAAGATGG	1
chr5	169395198	169395274	CCTTAAGAACATAAATCCCCAGGAATTCACAGAAACCTTGGTTT GAGCTTTGGATTTCCCGCAGGATGTGGGATAGG	2
chr5	171021380	171021457	CCATTCTCTCTGTCACTTTCAGGTACACCAATCAAACGTAGGTT TGGTCTTTTCTCATAGTCCCATATTTCTTGGAGG	1

chr5	173059898	173059973	CCATTTACCATCATTCTCTGTCATGGCAGGTGAAAGCAAGCTTT TATATAGACAATGTTCTACTTAGTTTACAGGG	4
chr5	174102359	174102435	CCCAAAGTTAATTTTACTCTTTTTCTGAATCAAAAGGAACCTTT CCTCCATGAGAAGAATCCTGCCATATTTCTAGG	2
chr5	180927811	180927888	CCTCCAAGAAATATGGGACTATGTGAAAAGACCAAACCTACGTT TGATTGCTATACATGAAAGTGACGGGGAGAATGG	1
chr6	1752363	1752440	CCTTCAAGAAATATGGGACTATGTGAAAAGACCAAACCTACCTT TGATTGGTGTACCTGAAAGTGATGGGAAGAATGG	1
chr6	20595279	20595356	CCATTCTCCCCATCACTTTCAGGTACACCAATCAAACGTAGGTT TGGTCTTTTCACATAGTCCCATAGTTCTTGGAGG	1
chr6	23431370	23431447	CCATTCTCCCCGTCACTTTCAGGGACAACAATCAAACGTAGGTT TGGCCTTTGCACATAGTCTTATATTTCTTGGAGG	1
chr6	29190624	29190701	CCATTCTCCCCATCACTTTCAGGTACACCAATCAAACGTAGGTT TGGTCTTTTCACATAGTCCCATATTTCTTGGAGG	1
chr6	61533266	61533343	CCTCCAAAAAAATATGGGACTATGTGAGAAGACCAAACCTACGTT TTATTAGTGTACCTCAAAGTGACAGGGAGGATGG	1
chr6	101052764	101052841	CCATTCTCCCCATCACTTTCAGGTACACCAATGAAACGTAGGTT TGGCCTTTTCACATAGTTTCATATTTCTTGGAGG	1
chr6	117176355	117176432	CCTCCAAGAAATATGGGACTATGTGAAAAGACCAAACCTACGTT TGATTGGTGTACCTGAAAGTGATGGGGAGAATGG	1
chr6	117747073	117747149	CCTACAAGAAATATGGAACTTGTAAAAAGACCAAACCTACGTTT GATTGGTGTACCTGAAAGTGACGGGGAGAATGG	2
chr6	118422508	118422585	CCTCCAAGAAATATGGGACAATGTGAAAAGGCCAAAGCTACGTT TGATTGGTGTACCTGAAAGTGACAGGGAGAATGG	1
chr6	122035019	122035096	CCTTTCAAACTTAGAGGTAAACAAAAGTCCTGAAAACCTAGGTT TGACCATAAGTTGGGACCATACGAGCATAGAAGG	1
chr6	134445210	134445287	CCAAAAATAAAAAAAAATTGACTTATAAGTAAGAAAGGTTCGTT TTCTCACATTCAGAAAGAGAACCCACATGTTGGG	1
chr6	134445210	134445286	CCAAAAATAAAAAAAAATTGACTTATAAGTAAGAAAGGTTCGTT TTCTCACATTCAGAAAGAGAACCCACATGTTGG	3
chr6	135154944	135155021	CCATTCTCCCCATCACTTTCAGGTACACCAATCAAACGTAGGTT TGGTCTTTTCACATAGTCCCATATTTCTTGGAGG	1
chr6	137889995	137890072	CCATTCTCCCCGTCACTTTCAGGTACACCAATCAAACGTTGGTT TAGTCTATTCACATAGTCCCATATTTCTTGGAGG	1
chr6	143993904	143993981	CCGAAAAGAATAAGACTATCAGCTGAAGTCTTAAAACGATCCTT TGGCCCCCAGTACTCTATATGCAGGATAGAAAGG	1
chr6	152610473	152610549	CCTACAAAAATAGGGGACTATGTGATAAGACCAAACCTACGTTT GATTGGTGTACCTGAAAGTGATGGGGAGAATGG	2
chr6	160372604	160372681	CCATTCTACCCATCACTTTCAGGTACACCAATCAAACGTAGGTT TGGCCTTTTCATATAGTCTCATATTTCTTGGAGG	1
chr6	169352478	169352555	CCATTCTCCCCATCACTTTCTGGTATACCAATCAAACGTAGGTT TGGTCTTTTCACATAGTCCCATATTTCTTAGAGG	1
chr6_GL000251v2_alt	677196	677273	CCATTCTCCCCATCACTTTCAGGTACACCAATCAAACGTAGGTT TGGTCTTTTCACATAGTCCCATATTTCTTGGAGG	1
chr6_GL000252v2_alt	456242	456319	CCATTCTCCCCATCACTTTCAGGTACACCAATCAAACGTAGGTT TGGTCTTTTCACATAGTCCCATATTTCTTGGAGG	1
chr6_GL000253v2_alt	456202	456279	CCATTCTCCCCATCACTTTCAGGTACACCAATCAAACGTAGGTT TGGTCTTTTCACATAGTCCCATATTTCTTGGAGG	1
chr6_GL000254v2	456371	456448	CCATTCTCCCCATCACTTTCAGGTACACCAATCAAACGTAGGTT TGGTCTTTTCACATAGTCCCATATTTCTTGGAGG	1

_alt				
chr6_GL000255v2_alt	456225	456302	CCATTCTCCCCATCACTTTCAGGTACACCAATCAAACGTAGGTTTGGTCTTTTCACATAGTCCCATATTTCTTGGAGG	1
chr6_GL000256v2_alt	500011	500088	CCATTCTCCCCATCACTTTCAGGTACACCAATCAAACGTAGGTTTGGTCTTTTCACATAGTCCCATATTTCTTGGAGG	1
chr7	5256551	5256627	CCACCACACCCAGCCTTATGGGATGGTTTTCAAAAGCATCCTTTTTTAGAAGTGGATTCTGATATATAATCGGATGG	2
chr7	7392583	7392660	CCATTCTCAATGTCACTTTCAGGTACACCAATCAAACGTAGGTTTGGTCTTTTCACATAGTCCCATATTTCTTGGAGG	1
chr7	8737741	8737818	CCATTCTCTCTGTCACTTTCAGGTACACCAGTCAAAGGTAGGTTTGTTTTATTCACACGTTCACATATTTCTTGGAGG	1
chr7	11352226	11352303	CCATTCGCCCCATCACTTTCAGGTACACTAGTAAAACGTAGGTTTGGTCTTTTCACATAGTTCCATATTTCTTGGAGG	1
chr7	15519145	15519222	CCTCCAAGAAATATGGGACTATGTGAAGAGATCAAACCTAGGTTTGATTGTTGTACCTGAAAGTGATAAGAAGAATGG	1
chr7	19228341	19228418	CCTCCAATAAATATGGGGCTATGTGAAAAGACCAAACCTACGTTTGATTGGTGTACCTGAAAGTGACAGGGAGAATGG	1
chr7	23778445	23778522	CCCTTTTCCCTGTCACTTTCAGGTACACCAGTCAAACGTAGGTTTGGTCTTTTCACATAGTCGAATATTTCTTCAAGG	1
chr7	23778446	23778522	CCTTTTCCCTGTCACTTTCAGGTACACCAGTCAAACGTAGGTTTGGTCTTTTCACATAGTCGAATATTTCTTCAAGG	2
chr7	26769065	26769142	CCATTCTCCCTGTCACTTTCAGGTACACTAATCAAACGTAGGTTTGGTGTATTCACACAGTCCCATATTTCTTGGAGG	1
chr7	42864035	42864112	CCATTCTTCCTGTCACTTTCAGGTATACCAATCAAACGTAGGTTTGGTCTTTTCACATAGTCCCATGTTTCTTGGAGG	1
chr7	46498923	46499000	CCTCCAAGAAATATGAGACTATATGAAAATACCAAACCTACGTTTGATTGGTGTACCTGAAAGAGACAGGGAGAATGG	1
chr7	51535360	51535437	CCATTCTCCCTATCACTTTCAGGTACACCAATCAAACGTAGGTTTGGTCTTTTCATGTAGTCCCATATTTCTTGGAGG	1
chr7	51927106	51927183	CCATTCTGCCCGTCACTTTCAGGTACACCAATCAAACGTAGGTTTGGTCTTTTCACATAGTCCCATATTTCTTGGAGG	1
chr7	56976942	56977018	CCGTCCGATTATATATCAGAATCTACTTCTAAAAAAGGATGCTTTTGAAAACCATCCCATAAGGCTGGGTGTGGTGG	3
chr7	80021598	80021675	CCTACAAGGAATATAGGACTATGTGAAAATACCAAACCTACGTTTCACTGCTGTACCTGAAGGTGACAGGGAGAATGG	1
chr7	89673853	89673930	CCATTCTCCCCATCATTTCCAGGTAAACCAATCAAAGGTAGGTTTGGTCATTTCACATAGTCCCATATTTCTTGGAGG	1
chr7	103404790	103404867	CCATTCTCCCCGTCACTTTCAGGTACACCAGTCAAACGTAGGTTTGGTCTTTTCACACAGTCCCATATTTCCTGGAGG	1
chr7	113053651	113053728	CCATTCTCCCCATCACTTTCAGGTACAGCAATCAAACGTAGGTTTGGTCTTTTCACATAGTCCCATATTTCTTGGAGG	1
chr7	125765204	125765279	CCACTACAGATTCTTGGGTCAAGATGTGTGCAAAAGGATGCTTTAGGGTGATGGATATGAGTGGGATGAAATGAGG	4
chr7	128042158	128042234	CCTGAAAAAAAACCCTGCCAGCCAGCAACTCTGAAAGGATGCTTTGTGTGAGTGAGCAGTGTCTGAGATGGACAGGG	3
chr7	130637332	130637409	CCATTCTCCCCATCACTTTCAGGTACGCCAATCAAACGTAGGTTTGGTCTTTTGACATAGTCCCATATTTCTTGGAGG	1
chr7	136983050	136983127	CCGTTCTCCCCATCACTTTTAGGTACACCAATCAAACGTAGGTTTGGTCTTTTCACATAGTCTCATATTTCTTGGAGG	1
chr7	143579507	143579584	CCATTCTCCTGGTCACTTTCAGGTATACCAATCAAACGTAGGTTTGGTCTTTTCATGTAGTCCCATATTTCTTGGAGG	1
chr7	143749881	143749958	CCTCCAAGAAATATGGGACTACATGAAAAGACCAAACCTACGTTTGATTGGTATACCTGAAAGTGACCAGGAGAATGG	1

chr8	2338364	2338441	CCTCCAAGAACTATGGGACTATGTGAAAAGACCAAACCTACGTT TGATTGGTGTACCTGAAAGTGACGGGGAGAATGG	1
chr8	2383289	2383366	CCATTCTCCCCGTCACTTTCAGGTACACCAATCAAACGTAGGTT TGGTCTTTTCACATAGTCCCATAGTTCTTGGAGG	1
chr8	8414568	8414645	CCATTCTCCCCGTCACTTTCAGGTACACCAATCAAACGTAGGTT TGGTCTTTTCACAGAGTCCCATATTTCTTGGAGG	1
chr8	24163142	24163219	CCATTCTCCCCGTCACTTTCATGTACACCAAGCAAACGTAGGTT TGATCTTTCCACATAGTCCCGTGTTTCTTGGAGG	1
chr8	34299051	34299128	CCTCCAAGAAATATGGGACTATGTGAAAAGACCAAACCTACGTT TGATTGGTGTACTTGAAAGTGACAGGGAGAATGG	1
chr8	40965485	40965562	CCTCCAAGAAATATGGGACTATGTGAAAAGACAAAACCTACGTT TCACTGGTGTACCTGAAAGTGACAGGGAGGATGG	1
chr8	48371659	48371735	CCCCCACCTTTTAAAAACATGCATACATACGGAAACGTTGCTTT CTGCACGATTTCATTTTAATGGAACAGAACAGG	2
chr8	82534960	82535037	CCATTTCCCCTGTCACTTTCAGGTACACCAATCAAACGTAGGTT TGGTCTTTTCACATAGTATCATATTTCTTGGAGG	1
chr8	109217624	109217700	CCATTCTCCCCGTCACTTTCAGGTACACCAATCAAACGTAGGTT TGGTCTTTTCACATAGTCCCATATTTCTGGAGG	3
chr8	134790285	134790361	CCTTTTGTTAAAGTAATAGAATTCTGCTTCTTAAAGGAACCTTT CAGGCAAGATGGTGGTTAGAGCACCTAAATGGG	2
chr8	134790285	134790360	CCTTTTGTTAAAGTAATAGAATTCTGCTTCTTAAAGGAACCTTT CAGGCAAGATGGTGGTTAGAGCACCTAAATGG	4
chr8_KI270821v1_alt	519635	519712	CCTCCAAGAACTATGGGACTATGTGAAAAGACCAAACCTACGTT TGATTGGTGTACCTGAAAGTGACGGGGAGAATGG	1
chr8_KI270821v1_alt	564557	564634	CCATTCTCCCCGTCACTTTCAGGTACACCAATCAAACGTAGGTT TGGCCTTTTCACATAGTCCCATAGTTCTTGGAGG	1
chr9	14951207	14951283	CCTCCAAGAAATATGGGACTGGTGAAAAGACCAAACCTACGTTT GACTGGTGTACCTGAAAGTGACGGGGAGACTGG	2
chr9	23249218	23249295	CCTCCAAGAAACATGGGAATGTGTGAAAAGACCAAACCTACGTT TGATTGGCGTACCTGAAAGTGACGGGGAGTATGG	1
chr9	26278896	26278973	CCTCCAAGAAATATGGGACTGTGTGAAAAGACCAAACCTACGTT TGATTGGTATACCTGAAAGTGACAGAGAGAATGG	1
chr9	27323237	27323314	CCATTCTCCCCTTCACTATCAGGTACACCAATCAAACGTAGGTT TAGTCTTTTCACATAGTCCCATATTTCTTGGAGG	1
chr9	31517993	31518070	CCATTCTCCCCGTCACTTTCAGATACACCAGTCAAACGTAGGTT TGGTCTTTTCACATAGTCCCATATTTCTTGGAGG	1
chr9	39694860	39694937	CCATCTTACTTTGTACTACACTGTTCTTTAGAGAAAGCTTCCTT TTGGAGACCAACCAGGACTCCTTAGAAGCAGAGG	1
chr9	42451132	42451209	CCATCTTACTTTGTACTACACTGTTCTTTAGAGAAAGCTTCCTT TTGGAGACCAACCAGGACTCCTTAGAAGCAGAGG	1
chr9	60776573	60776650	CCTCTGCTTCTAAGGAGTCCTGGTTGGTCTCCAAAAGGAAGCTT TCTCTAAAGAACAGTGTAGTACAAAGTAAGATGG	1
chr9	62647482	62647559	CCTCTGCTTCTAAGGAGTCCTGGTTGGTCTCCAAAAGGAAGCTT TCTCTAAAGAACAGTGTAGTACAAAGTAAGATGG	1
chr9	66682030	66682107	CCTCTGCTTCTAAGGAGTCCTGGTTGGTCTCCAAAAGGAAGCTT TCTCTAAAGAACAGTGTAGTACAAAGTAAGATGG	1
chr9	82264427	82264503	CCACCACTGTGCCTGGCCATTTTCACTATTCTTAAAGGAAGCTT TGGTTTACAAAGGTTTGCTACTGTACTTCCAGG	3
chr9	84042684	84042761	CCATTCTCCCTGTCACTTTCAGGTACACCATTCAAACGTAGGTT TGGTCTTTTCTCATAGTCCCATATTTCTTGGAGG	1
chr9	95256012	95256089	CCTCCAAGAAATTCGGGACTATGTGAAAAGACAAAACCTACGTT TAATTGGTGTGTGGTGTACCTGAAAGTGACAAGG	1
chr9	101816988	101817065	CCTCCAAGAAATATGGGACTATGTGAAAAGACCAAACCTACGTT TGATTGGTGTACCTGAAAGTGACCAGAAGAATGG	1
chr9	135842327	135842403	CCTCCAAGAAATATGGGACTATGTGAAAAGCCCAAACCTACGTT	3

			TGACTGATGTACCTAAAGTGACGGGGAGAATGG	
chr9	136910865	136910940	CCCGCACTGTGAGCTTGGCCGAGTGCTGTCTGAAAGCATCCTTTCCCTTCACCTGGAGACTGGAGCGCCATAGAGG	4
chr10	13710312	13710389	CCTGTCTCCCCCATTCCATGCAAAATAAAACACAAACCAAGCTTTGCTTTAAGTGCTCCCTGATGCAGTTCAGCGTGG	1
chr10	18938129	18938206	CCATTCTTCCCGTCACATTCAGGTACACCAATCAAACGTAGGTTTGGTCTTTTCCCATAGTCCCATATTTCTTAGAGG	1
chr10	22712838	22712914	CCCCCTGCTCAGCTTGGGGAAGAAAAATACAAAAACGATGCTTTTAGGCATTTTAAACAACTTCACTACATTGAGGG	2
chr10	22712838	22712913	CCCCCTGCTCAGCTTGGGGAAGAAAAATACAAAAACGATGCTTTTAGGCATTTTAAACAACTTCACTACATTGAGG	4
chr10	40160932	40161009	CCTTTGTGTTGTGTGTATTCAACTCACAGAGTGAAACCTTCCTTTATTCAGAGCAGTTTTGAAACACTCTTTTTGTGG	1
chr10	40390136	40390213	CCTTTGTGTTGTGTGTATTCAACTCACAGAGTGAAACCTTCCTTTATTCAGAGCAGTTTTGAAAAACACTTTTTGTGG	1
chr10	40409152	40409229	CCTTTGTGTTGTGTGTATTCAACTCACAGAGTGAAACCTTCCTTTATTCAGAGCAGTTTTGAAAAACTCTTTTTGTGG	1
chr10	40433940	40434017	CCTTTGTGTTGTGTGTATTCAACTCACAGAGTGAAACCTTCCTTTATTCAGAGCAGTTTTGAAACACTCTTTTTGTGG	1
chr10	40588155	40588232	CCTTTGTGTTGTGTGTATTCAACTCACAGAGTGAAACCTTCCTTTATTCAGAGCAGTTTTGAAATACTCTTTTTGTGG	1
chr10	41146207	41146284	CCTTTGTGTTGTGTGTATTCAACTCACAGAGTGAAACCTTCCTTTATTCAGAGCAGTTTTGAAACACTCTTTTTGTGG	1
chr10	43835183	43835260	CCATTCTCCCTGTCACTTTCAAGTACACCAATCAAACCTAGGTTTGGTCTTTTCACATAGTTCCATATTTCTTGGAGG	1
chr10	54913222	54913299	CCCCTCCCATCACAGGCCCTGAGGTTTAAGAGAAAACCATGGTTTTGTGGGCCAGGCCCATGACCCTTCTCCTCTGGG	1
chr10	54913222	54913298	CCCCTCCCATCACAGGCCCTGAGGTTTAAGAGAAAACCATGGTTTTGTGGGCCAGGCCCATGACCCTTCTCCTCTGG	3
chr10	54913223	54913299	CCCTCCCATCACAGGCCCTGAGGTTTAAGAGAAAACCATGGTTTTGTGGGCCAGGCCCATGACCCTTCTCCTCTGGG	2
chr10	54913223	54913298	CCCTCCCATCACAGGCCCTGAGGTTTAAGAGAAAACCATGGTTTTGTGGGCCAGGCCCATGACCCTTCTCCTCTGG	4
chr10	58035951	58036028	CCATTCTCCCCATCACTTTCAGGTACACCAATCAAACGTAGGTTTCATCTTTTCACATAGTCCCACGGTTTTTGGAGG	1
chr10	58677525	58677602	CCTCCAAGATATATGGGACTATGTGAAAAGACCAAACCTACGTTTGATTGGTGTACCTGAAATTGATGGGGAGAATGG	1
chr10	84021390	84021467	CCTCCAAGAAATATGGGACTGTGTGAAAAGAACAAACCTACGTTTGATTGGTGTACGTGAAAGTGATGGGGAGAATGG	1
chr10	91442692	91442769	CCATTCCTCCCGTCACTTTCAGATACACCAAAAAAACGTAGGTTTGGTCTCTTCACATAGTCCCACATTTCTTGGAGG	1
chr10	91446848	91446925	CCTCCAAGAAATGTGGGACTATGTGAAGAGACCAAACCTACGTTTTTTTGGTGTATCTGAAAGTGACGGGAGGAATGG	1
chr10	116928784	116928860	CCTCCAAGGGGAATCTGAGTTCTCTGAAGACAAAAAGCATGGTTTCTTTTCTTCTGTATTTCTTATTGTTTCCTAGG	3
chr10	116937771	116937848	CCATTCTCCCTATCACTTTCCAGTACACCAATCAAACGTAGGTTTGGTCTTTTCACATAGTCCCATATTTCTTGGAGG	1
chr11	31182070	31182147	CCTCCAAGAAATATGGGACTATGTGAAAAGACCAAACCTACGTTTGATTGGTATACTTGAAATTGACAAGGAGAATGG	1
chr11	34739273	34739350	CCTCCAAGAAATATGGGACTATGTGGAAAGACCAAACCTACGTTTGACTGGTGTACCTGAAAGTGATGGGGAGAATGG	1
chr11	86646529	86646606	CCTCTAAGAAATATGGGACTATGTGAAGAGATGAAACCTACGTTTGATTGGTGTACCTGAAAGTGACGAGGAGAATGG	1
chr11	90469791	90469867	CCCTCGTATACTACATGCTATAGTCAAAGCAGTAAACCTTCCTTTCCTTAAGCAGACCACACTCTTTCATGCCTGGG	3
chr11	90469792	90469867	CCTCGTATACTACATGCTATAGTCAAAGCAGTAAACCTTCCTTTCCTTAAGCAGACCACACTCTTTCATGCCTGGG	4
chr11	92429985	92430062	CCATTCTCCCCATCACTTTCAGGTATACTAATCAAAGGTAGGTTTGGTCTTTTCACATAGTCCCATATTTCATGGAGG	1
chr1	102818498	102818574	CCATTCCCCCGTCACTTTCAGGTACACCAATCAAACGTAGGTTT	2

1			GGTCTTTTCACATAGTCCCATATTTCTTGGAGG	
chr11	120765065	120765142	CCATTCTCCCCGTCACTTTCAGGTACACCAATCAAACGTAGGTTTTGTCTTTTCTTATAGTCCCATATTTCTTGGAGG	1
chr11	123131901	123131978	CCACTGCACCTGACCAAGATCCTTAATTTTTCTAAACCTACGTTTATCATCTATAAAATGAGCCATCTTTTCACATGG	1
chr11	129468520	129468597	CCTCCGAGAAATATGGGACTATGTGAAAAGACCAAACCTACGTTTGATTGTTGTACCTGAAAGTGACAGGGAGAATGG	1
chr11	131272361	131272438	CCATTCTCCCCATCACTTTTAGGTACACCAATCAAACGTAGGTTTGGTCCTTTTGCATAGACCCATATTTCTTGGAGG	1
chr11	132761415	132761492	CCATTTTCCCCGTCAGTTTCATATACACCTATCAAACGTAGGTTTACTGTTTTCACATAGTCCCTTATTTCTTGGAGG	1
chr12	22367416	22367493	CCTCCAAGAAATATGGGACTATGTGAAAAGACCAAACCTACCTTTGATTGGTGTACCTGAAAGTGACGGGCAGGATGG	1
chr12	33146384	33146461	CCATTCTTCTCGTCATTTTCAAGTACACCAATCAAACGTAGGTTTGGTCTTTTCGCATAGTCCCATATTTCTTGGAGG	1
chr12	33198476	33198553	CCATTCTTCTCGTCACTTTCAAGTACACCAATCAAACGTAGGTTTGGTCTTTTCACATAGTCCCATATTTCTTGGAGG	1
chr12	46038332	46038409	CCTCCAAGAAATATAGGACTATGTGAAAAGACCAAACCTACGTTTGATTGGTGTACTTGAAAGTGACAGGGAGAATGG	1
chr12	60236126	60236203	CCTCCAAGAAATGTGGAACTATGTGAAAAGACCAAACCTACGTTTGATTGGTGTACCTGAAAGTGACAGGGAGAATGG	1
chr12	62098359	62098434	CCCTGACACTGATAAACGGATATGAAGAGAAAAAAGCTAGGTTTTCGCTGGAATTCCTAAGCTTGGGCTGCAGTGG	4
chr12	62112591	62112668	CCCTTCTCCCAGTCACTTTTAGGTACACCAATGAAACGTAGGTTTGGTCTTTTCACACAGTCCCATATTTCTTGGAGG	1
chr12	62112592	62112668	CCTTCTCCCAGTCACTTTTAGGTACACCAATGAAACGTAGGTTTGGTCTTTTCACACAGTCCCATATTTCTTGGAGG	2
chr12	62418577	62418652	CCACTCCCTCTCCCCCAAAAAGTAAAGGTAGAAAACCAAGGTTTACAGGCAACAAATAGCACAATGAATGGAATGG	4
chr12	71732311	71732388	CCAAACCCGCATCGCACACCCTGTGAGGGGGACAAAGGAACCTTTCCGTTCCAACATCAAGGTTGTTTGACCCAAGG	1
chr12	78047816	78047893	CCATTCTTTCTGTCACTTTCAGGTATACCAGTCAAACCTAGGTTTGGTCTTTTCACATAGTCCCATATTTCTTGGAGG	1
chr12	81480016	81480093	CCATTCTCCCCATCACTTTCAGGTACACCAATCAAACGTAGGTTTGGTCTTTTCACATAGTCCCATATTTCTTGGAGG	1
chr12	96840231	96840307	CCACACGGTAGAGGATAAACTAGGTGGATTCTCAAAGCAACCTTTGAAATAATCTATGCAGTTTTTCTGGGTACTGG	3
chr12	99187165	99187242	CCACCAAGAAACATGGGACTATGTGAAAAGACCAAACCTACGTTTGGTTGGTGTACCTGGAAGTGACGGGGAGAGTGG	1
chr12	107860841	107860918	CCTCCAAGAAATATGGGACCATGTGAAAAGACCAAACCTACGTTTGATTGGTGTACCTGAAAGTGACAGGGAGAATGG	1
chr12	110882809	110882885	CCTGTAAAAAGGTCACATGGTCAGGTGTGCCTAAACGATCCTTTTATTTATTTATTTATTTATTTTTAAGAAACAGG	2
chr12	119063321	119063397	CCAGCCCCAAAATGTCAGGGGCTTAGAACAACAAAGGTTCCTTTTCATGTTTATACTACATGTTTGTCATGGGCTGG	2
chr13	35320704	35320781	CCGTTTTCCCCATCACTTTCAGGTACACCAGTCAAACGTAGGTTTGGTCTTTTCACATGGTCCCACATTTCTTGGAGG	1
chr13	53133477	53133554	CCTGGAATAGCTTTCCTGACTGTCTGACTTCAAAAACCTTGGTTTGACCACTTCGTCTATATCATGAGGAAGGACTGG	1
chr13	53184880	53184956	CCCTACTCTGAACCTACCTTGATAAAGCCTAGAAAACCAAGCTTTGACAAGATTTGACAAGAGATGGAATTTGGAGG	3
chr13	53184881	53184956	CCTACTCTGAACCTACCTTGATAAAGCCTAGAAAACCAAGCTTTGACAAGATTTGACAAGAGATGGAATTTGGAGG	4
chr13	57896962	57897038	CCCTTATAAAACTGAAAACTTTAACCTTTTTTAAAGCATGCTTTTGAATAAATTCTTTTATTACAAAAAAGACCAGG	2
chr13	62610100	62610177	CCATTCTCCCTGTCACTTTCAGGTACACCAATCAAACGTAGGTTTGGTCTTTTCACGTAGTCCCATATTTCTTGGAGG	1
chr13	77004382	77004458	CCCTTTATTATCCAAGTGGTTTCCTGCTCTTCAAACCTTCCTTTCAAAATTTTGTCTCCTACTTAAAACAAGTTAGG	2
chr1	81646075	81646151	CCTTCTGTTGAGACCTACTGCTAAGAAAACAAAAAAGGTTCCTT	3

3			TCAAATATTATTGTGAATCAATAATGTACCTGG	
chr13	83755854	83755931	CCTCCAAGAAATATGGGACTATGTGAAAAGACCAAACCTACGTTTCATTGATGGACCTGAAAGTGATGGGGAGAATGG	1
chr13	89719199	89719275	CCATTCTCCCTTCACTTTCAGTTACACCAATCAAACGTAGGTTTGGTCTTTTCACATAGTCCCATATTTCTTGGAGG	2
chr13	102010574	102010650	CCTAGGGAAGTGATCATAGCTGAGTTTCTGGAAAAACCTAGGTTTTAAAGTTGAGGAGACTTAAGTCCAAAACCTGG	3
chr13_KI270841v1_alt	124240	124316	CCATTCTCCCTTCACTTTCAGTTACACCAATCAAACGTAGGTTTGGTCTTTTCACATAGTCCCATATTTCTTGGAGG	2
chr14	25980646	25980723	CCTCCAAGAAATATGGGACTATGTGAAAAGACTAAACCTACGTTTGATTGGTGTACCTGAAAGTGACAGGGAGAATGG	1
chr14	35842786	35842863	CCATTCTCCCTGTCACTTTCAGGTATGCCAGTCAAACGTAGGTTTGGTCTTTTCACATAGTCCCATATTCCTTGGAGG	1
chr14	42646400	42646477	CCTCCAAGAAATATGGGACTATGTAAAAAGACGAAACCTACGTTTGATTGGTGTACTTAAAAGTGACGAGGAGAATGG	1
chr14	49063242	49063319	CCTCCAAGAAATATGGGACTATGTGAAAAGACCAAACCTACGTTTGATTTGTGTACCTGAAAGTGATGGGGAGAATGG	1
chr14	49130379	49130456	CCATTCTCCCCGTCACTTTCAGGCACACCAATCAAACGTAGGTTTAGTCTTTTCACATAGTCCCATATTTCTTAGAGG	1
chr14	51352342	51352418	CCTTAATGCATTCATATTTCATATTTTAAATAAAACCATGGTTTCCCACAGAGTGACTTCTACTCTAAGAAATGGGG	2
chr14	51352342	51352417	CCTTAATGCATTCATATTTCATATTTTAAATAAAACCATGGTTTCCCACAGAGTGACTTCTACTCTAAGAAATGGG	4
chr14	60835842	60835919	CCGTTCTTTCCGTCACTTTCAGGTACACCAGTCAAACGTAGGTTTGGTCTTTTCACATAGTCCCATATTTCTTGGAGG	1
chr14	66529072	66529148	CCATTCTCCCCATCACTTTCATGTACACCAATCAAACGTAGGTTTGGTCTTTGTTAACATAGTCCCATATTTCTTGG	3
chr14	79210873	79210949	CCCTATAAAGCTTAGAGAAACACAGGGCTCTTTAAACGATCCTTTTTCTCTTTTCTGTTTTAAATTTCATCACTTGG	3
chr14	79210874	79210949	CCTATAAAGCTTAGAGAAACACAGGGCTCTTTAAACGATCCTTTTTCTCTTTTCTGTTTTAAATTTCATCACTTGG	4
chr14	85371541	85371618	CCATTCTCCCCATCACTTTCAGGTACACTAATCAAAGGTAGGTTTGGTCTTTTCACATGGTCCTATATTTCTTGGAGG	1
chr14	92918713	92918790	CCCCATAGCACGATCACATGGGACATTCAGGGGAAAGCAACCTTTTCCAGGAAGGAAAACCCAATGCTGGGACCCAGG	1
chr14	92918714	92918790	CCCATAGCACGATCACATGGGACATTCAGGGGAAAGCAACCTTTTCCAGGAAGGAAAACCCAATGCTGGGACCCAGG	2
chr14	103386821	103386897	CCCTTTCAGCGCTCACAGGCTATGGTTTTATAAAAGGAACCTTTGATTTTGTTCATGTGAAACTACAAAATGCCAGG	2
chr14_KI270847v1_alt	33275	33352	CCCCATAGCACGATCACATGGGACATTCAGGGGAAAGCAACCTTTTCCAGGAAGGAAAACCCAATGCTGGGACCCAGG	1
chr14_KI270847v1_alt	33276	33352	CCCATAGCACGATCACATGGGACATTCAGGGGAAAGCAACCTTTTCCAGGAAGGAAAACCCAATGCTGGGACCCAGG	2
chr15	20630566	20630643	CCTCCAAGAAATATTGGAGTATGTGATAAGACCAAACCTTCGTTTGACTGGTGTACCTGAAAGTGATGGGGAGAATGG	1
chr15	21675103	21675180	CCATTCTCCCCGTCACTTTCAGGTACACCAATCAAACGTAGGTTTGGTCTTTTCACATAGTCCCATATTTCTTGGAGG	1
chr15	22117571	22117648	CCATTCTCCCCGTCACTTTCAGGTACACCAATCAAACGTAGGTTTGGTCTTTTCACATAGTCCCATATTTCTTGGAGG	1
chr15	22369744	22369821	CCATTCTCCCCATCACTTTCAGGTACACCAGTCAAACGAAGGTTTGGTCTTATCACATACTCCAATATTTCTTGGAGG	1

chr15	42302832	42302909	CCTCCAAGATATATGGGACTATGTGAAAAGGCCAAACCTACCTTTGATTGATACACCTGAAAATGACAGGGAGAATGG	1
chr15	49967601	49967678	CCTCCAAGAAATATGCGACTATGTGAAAAGACCAAACCTACGTTTCATTGGTGTACCTGAAAGTGATGGGGAGAATGG	1
chr15	83964501	83964577	CCTCCAAGAAATATGGGACTATGTGGAAAGACCAAACCTACGTTTGTTTGGTGTACCTGAAAGTGAGGGGAGAATGG	3
chr15	87261388	87261465	CCATTCTCCTCATCACTTTCAAGTACACCAATCAAACGTAGGTTTGGTCTTTTCACATAGTCTTATATTTCTTGGAGG	1
chr15_KI270727v1_random	409348	409425	CCATTCTCCCCGTCACTTTCAGGTACACCAATCAAACGTAGGTTTGGTCTTTTCACATAGTCCCATATTTCTTGGAGG	1
chr15_KI270851v1_alt	14235	14312	CCATTCTCCCCATCACTTTCAGGTACACCAGTCAAACGAAGGTTTGGTCTTATCACATACTCCAATATTTCTTGGAGG	1
chr15_KI270852v1_alt	440099	440176	CCATTCTCCCCGTCACTTTCAGGTACACCAATCAAACGTAGGTTTGGTCTTTTCACATAGTCCCATATTTCTTGGAGG	1
chr16	22123671	22123748	CCAGCAGAAGAATCTGGGGCACAGTCTGTGAAAAAAGGTACCTTTCTTAAGCAGGGTTCTTATCCTTCATGGGTCTGG	1
chr16	25557623	25557700	CCTCCAAGAAATATGGGACTATGTGAAAAGACCAAACCTACGTTTGATTGTTGTACCTGAAAGTGAGGGGGAGAATGG	1
chr16	36427179	36427255	CCTTGTGTTGTGTGTATTCAACTCACAGAGTTAAACGATCCTTTACACAGAGCAGATTTGAAACACTGTTTTTCTGG	2
chr16	36476450	36476526	CCTTGTGTTGTGTGTATTCAACTCACAGAGTTAAACGATCCTTTACACAGAGCAGATTTGAAACACTGTTTTTCTGG	2
chr16	36512469	36512545	CCTTGTGTTGTGTGTATTCAACTCACAGAGTTAAACGATCCTTTACACAGAGCAGATTTGAAACACTGTTTTTCTGG	2
chr16	36520964	36521040	CCTTGTGTTGTGTGTATTCAACTCACAGAGTTAAACGATCCTTTACACACAGCAGATTTGAAACACTGTTTTTCTGG	2
chr16	36524704	36524780	CCTTGTGTTGTGTGTATTCAACTCACAGAGTTAAACGATCCTTTACACAGAGCAGATTTGAAACACTGTTTTTCTGG	2
chr16	36566812	36566888	CCTTGTGTTGTGTGTATTCAACTCACAGAGTTAAACGATCCTTTACACAGAGCAGATTTGAAACACTGTTTTTCTGG	2
chr16	36573603	36573679	CCTTGTGTTGTGTGTATTCAACTCACAGAGTTAAACGATCCTTTACACAGAGCAGATTTGAAACACTGTTTTTCTGG	2
chr16	36667694	36667770	CCTTGTGTTGTGTGTATTCAACTCACAGAGTTAAACGATCCTTTACACAGAGCAGATTTGAAACACTGTTTTTCTGG	2
chr16	36677320	36677396	CCTTGTGTTGTGTGTATTCAACTCACAGAGTTAAACGATCCTTTACACAGAGCAGATTTGAAACACTGTTTTTCTGG	2
chr16	36683096	36683172	CCTTGTGTTGTGTGTATTCAACTCACAGAGTTAAACGATCCTTTACACAGAGCAGATTTGAAACACTGTTTTTCTGG	2
chr16	36691251	36691327	CCTTGTGTTGTGTGTATTCAACTCACAGAGTTAAACGATCCTTTACACAGAGCAGATTTGAAACACTGTTTTTCTGG	2
chr16	36710951	36711027	CCTTGTGTTGTGTGTATTCAACTCACAGAGTTAAACGATCCTTTACACAGAGCAGATTTGAAACACTGTTTTTCTGG	2
chr16	36750364	36750440	CCTTGTGTTGTGTGTATTCAACTCACAGAGTTAAACGATCCTTTACACAGAGCAGATTTGAAACACTGTTTTTCTGG	2
chr16	36791455	36791531	CCTTGTGTTGTGTGTATTCAACTCACAGAGTTAAACGATCCTTTACACACAGCAGATTTGAAACACTGTTTTTCTGG	2
chr16	36856683	36856759	CCTTGTGTTGTGTGTATTCAACTCACAGAGTTAAACGATCCTTTACACAGAGCAGATTTGAAACACTGTTTTTCTGG	2
chr16	36926655	36926731	CCTTGTGTTGTGTGTATTCAACTCACAGAGTTAAACGATCCTTTACACAGAGCAGATTTGAAACACTGTTTTTCTGG	2

chr16	36931752	36931828	CCTTGTGTTGTGTGTATTCAACTCACAGAGTTAAACGATCCTTTACACAGAGCAGATTTGAAACACTGTTTTTCTGG	2
chr16	36948058	36948134	CCTTGTGTTGTGTGTATTCAACTCACCGAGTTAAACGATCCTTTACACAGAGCAGATTTGAAACACTGTTTTTCTGG	2
chr16	36974541	36974617	CCTTGTGTTGTGTGTATTCAACTCACAGAGTTAAACGATCCTTTACACAGAGCAGATTTGAAACACTGTTTTTCTGG	2
chr16	36981331	36981407	CCTTGTGTTGTGTGTATTCAACTCACAGAGTTAAACGATCCTTTACACAGAGCAGATTTGAAACACTGTTTTTCTGG	2
chr16	36990839	36990915	CCTTGTGTTGTGTGTATTCAACTCACAGAGTTAAACGATCCTTTACACAGAGCAGATTTGAAACACTGTTTTTCTGG	2
chr16	37021075	37021151	CCTTGTGTTGTGTGTATTCAACTCACAGAGTTAAACGATCCTTTACACAGAGCAGATTTGAAACACTGTTTTTCTGG	2
chr16	37042812	37042888	CCTTGTGTTGTGTGTATTCAACTCACAGAGTTAAACGATCCTTTACACAGAGCAGATTTGAAACACTGTTTTTCTGG	2
chr16	37085971	37086047	CCTTGTGTTGTGTGTATTCAACTCACAGAGTTAAACGATCCTTTACACAGAGCAGATTTGAAACACTGTTTTTCTGG	2
chr16	37129462	37129538	CCTTGTGTTGTGTGTATTCAACTCACAGAGTTAAACGATCCTTTACACAGAGCAGATTTGAAACACTGTTTTTCTGG	2
chr16	37146110	37146186	CCTTGTGTTGTGTGTATTCAACTCACAGAGTTAAACGATCCTTTACACACAGCAGATTTGAAACACTGTTTTTCTGG	2
chr16	37157309	37157385	CCTTGTGTTGTGTGTATTCAACTCACAGAGTTAAACGATCCTTTACACAGAGCAGATTTGAAACACTGTTTTTCTGG	2
chr16	37183118	37183194	CCTTGTGTTGTGTGTATTCAACTCACAGAGTTAAACGATCCTTTACACAGAGCAGATTTGAAACACTGTTTTTCTGG	2
chr16	37190924	37191000	CCTTGTGTTGTGTGTATTCAACTCACAGAGTTAAACGATCCTTTACACAGAGCAGATTTGAAACACTGTTTTTCTGG	2
chr16	37221808	37221884	CCTTGTGTTGTGTGTATTCAACTCACAGAGTTAAACGATCCTTTACACAGAGCAGATTTGAAACACTGTTTTTCTGG	2
chr16	37259501	37259577	CCTTGTGTTGTGTGTATTCAACTCACAGAGTTAAACGATCCTTTACACAGAGCAGATTTGAAACACTGTTTTTCTGG	2
chr16	37272409	37272485	CCTTGTGTTGTGTGTATTCAACTCACAGAGTTAAACGATCCTTTACACAGAGCAGATTTGAAACACTGTTTTTCTGG	2
chr16	37281923	37281999	CCTTGTGTTGTGTGTATTCAACTCACAGAGTTAAACGATCCTTTACACAGAGCAGATTTGTAACACTGTTTTTCTGG	2
chr16	37346472	37346548	CCTTGTGTTGTGTGTATTCAACTCACAGAGTTAAACGATCCTTTACACAGAGCAGATTTGAAACACTGTTTTTCTGG	2
chr16	37357000	37357076	CCTTGTGTTGTGTGTATTCAACTCACAGAGTTAAACGATCCTTTACACAGAGCAGATTTGAAACACTGTTTTTCTGG	2
chr16	37373301	37373377	CCTTGTGTTGTGTGTATTCAACTCACAGAGTTAAACGATCCTTTACACAGAGCAGATTTGAAACACTGTTTTTCTGG	2
chr16	37419498	37419574	CCTTGTGTTGTGTGTATTCAACTCACAGAGTTAAACGATCCTTTACACAGAGCAGATTTGAAACACTGTTTTTCTGG	2
chr16	37430714	37430790	CCTTGTGTTGTGTGTATTCAACTCACAGAGTTAAACGATCCTTTACACAGAGCAGATTTGAAACACTGTTTTTCTGG	2
chr16	37455845	37455921	CCTTGTGTTGTGTGTATTCAACTCACAGAGTTAAACGATCCTTTACACAGAGCAGATTTGAAACACTGTTTTTCTGG	2
chr16	37458558	37458634	CCTTGTGTTGTGTGTATTCAACTCACAGAGTTAAACGATCCTTTACACAGAGCAGATTTGAAACACTGTTTTTCTGG	2
chr16	37486127	37486203	CCTTGTGTTGTGTGTATTCAACTCACAGAGTTAAACGATCCTTTACACAGAGCAGATTTGAAACACTGTTTTTCTGG	2
chr16	37525183	37525259	CCTTGTGTTGTGTGTATTCAACTCACAGAGTTAAACGATCCTTTACACAGAGCAGATTTGAAACACTGTTTTTGTGG	2
chr16	37536735	37536811	CCTTGTGTTGTGTGTATTCAACTCACAGAGTTAAACGATCCTTTACACAGAGCAGATTTGAAACACTGTTTTTCTGG	2
chr16	37554730	37554806	CCTTGTGTTGTGTGTATTCAACTCACAGAGTTAAACGATCCTTTACACAGAGCAGATTTGAAACACTGTTTTTCTGG	2
chr16	37575784	37575860	CCTTGTGTTGTGTGTATTCAACTCACAGAGTTAAACGATCCTTTACACAGAGCAGATTTGAAACACTGTTTTTCTGG	2
chr16	37577483	37577559	CCTTGTGTTGTGTGTATTCAACTCACAGAGTTAAACGATCCTTTACACAGAGCAGATTTGAAACACTGTTTTTCTGG	2

chr16	37583598	37583674	CCTTGTGTTGTGTGTATTCAACTCACAGAGTTAAACGATCCTTTACACAGAGCAGATTTGAAACACTGTTTTTCTGG	2
chr16	37696368	37696444	CCTTGTGTTGTGTGTATTCAACTCACAGAGTTAAACGATCCTTTCCACAGAGCAGATTTGAAACACTGTTTTTCTGG	2
chr16	37704524	37704600	CCTTGTGTTGTGTGTATTCAACTCACAGAGTTAAACGATCCTTTACACAGAGCAGATTTGAAACACTGTTTTTCTGG	2
chr16	37706223	37706299	CCTTGTGTTGTGTGTATTCAACTCACAGAGTTAAACGATCCTTTACACAGAGCAGATTTGAAACACTGTTTTTCTGG	2
chr16	37708941	37709017	CCTTGTGTTGTGTGTATTCAACTCACAGAGTTAAACGATCCTTTACACAGAGCAGATTTGAAACACTGTTTTTCTGG	2
chr16	37763622	37763698	CCTTGTGTTGTGTGTATTCAACTCACAGAGTTAAACGATCCTTTACACAGAGCAGATTTGAAACACTGTTTTTCTGG	2
chr16	37772115	37772191	CCTTGTGTTGTGTGTATTCAACTCACAGAGTTAAACGATCCTTTACACAGAGCAGATTTGAAACACTGTTTTTCTGG	2
chr16	37791815	37791891	CCTTGTGTTGTGTGTATTCAACTCACAGAGTTAAACGATCCTTTACACAGAGCAGATTTGAAACACTGTTTTTCTGG	2
chr16	37796229	37796305	CCTTGTGTTGTGTGTATTCAACTCACAGAGTTAAACGATCCTTTACACAGAGCAGATTTGAAACACTGTTTTTCTGG	2
chr16	37797928	37798004	CCTTGTGTTGTGTGTATTCAACTCACAGAGTTAAACGATCCTTTACACAGAGCAGATTTGAAACACTGTTTTTCTGG	2
chr16	37843453	37843529	CCTTGTGTTGTGTGTATTCAACTCACAGAGTTAAACGATCCTTTACACAGAGCAGATTTGAAACACTGTTTTTCTGG	2
chr16	37848548	37848624	CCTTGTGTTGTGTGTATTCAACTCACAGAGTTAAACGATCCTTTACACAGAGCAGATTTGAAACACTGTTTTTCTGG	2
chr16	37864846	37864922	CCTTGTGTTGTGTGTATTCAACTCACCGAGTTAAACGATCCTTTACACAGAGCAGATTTGAAACACTGTTTTTCTGG	2
chr16	37902550	37902626	CCTTGTGTTGTGTGTATTCAACTCACAGAGTTAAACGATCCTTTACACAGAGCAGATTTGAAACACTGTTTTTCTGG	2
chr16	37907307	37907383	CCTTGTGTTGTGTGTATTCAACTCACAGAGTTAAACGATCCTTTACACAGAGCAGATTTGAAACACTGTTTTTCTGG	2
chr16	37928033	37928109	CCTTGTGTTGTGTGTATTCAACTCACAGAGTTAAACGATCCTTTACACAGAGCAGATTTGAAACACTGTTTTTCTGG	2
chr16	37959262	37959338	CCTTGTGTTGTGTGTATTCAACTCACAGAGTTAAACGATCCTTTACACAGAGCAGATTTGAAACACTGTTTTTCTGG	2
chr16	37964355	37964431	CCTTGTGTTGTGTGTATTCAACTCACAGAGTTAAACGATCCTTTACACAGAGCAGATTTGAAACACTGTTTTTCTGG	2
chr16	37974881	37974957	CCTTGTGTTGTGTGTATTCAACTCACAGAGTTAAACGATCCTTTACACAGAGCAGATTTGAAAAACTGTTTTTCTGG	2
chr16	37987789	37987865	CCTTGTGTTGTGTGTATTCAACTCACAGAGTAAAACGATCCTTTACACAGAGCAGATTTGAAACACTGTTTTTCTGG	2
chr16	37994586	37994662	CCTTGTGTTGTGTGTATTCAACTCACAGAGTTAAACGATCCTTTACACAGAGCAGATTTGAAACACTGTTTTTGTGG	2
chr16	38006479	38006555	CCTTGTGTTGTGTGTATTCAACTCACAGAGTTAAACGATCCTTTACACAGAGCAGATTTGAAACACTGTTTTTCTGG	2
chr16	38011567	38011643	CCTTGTGTTGTGTGTATTTAACTCACAGAGTTAAACGATCCTTTACACAGAGCAGATTTGAAACACTGTTTTTCTGG	2
chr16	38040096	38040172	CCTTGTGTTGTGTGTATTCAACTCACAGAGTTAAACGATCCTTTACACAGAGCAGATTTGAAACACTGTTTTTCTGG	2
chr16	38041456	38041532	CCTTGTGTTGTGTGTATTCAACTCACAGAGTTAAACGATCCTTTACACAGAGCAGATTTGAAACACTGTTTTTCTGG	2
chr16	38062179	38062255	CCTTGTGTTGTGTGTATTCAACTCACAGAGTTAAACGATCCTTTACACAGAGCAGATTTGAAACACTGTTTTTCTGG	2
chr16	38102937	38103013	CCTTGTGTTGTGTGTATTCAACTCACAGAGTTAAACGATCCTTTACACAGAGCAGATTTGAAACACTGTTTTTCTGG	2
chr16	38128412	38128488	CCTTGTGTTGTGTGTATTCAACTCACAGAGTTAAACGATCCTTTACACAGAGCAGATTTGAAACACTGTTTTTCTGG	2
chr16	38131809	38131885	CCTTGTGTTGTGTGTATTCAACTCACAGAGTTAAACGATCCTTTACACAGAGCAGATTTGAAACACTGTTTTTCTGG	2
chr16	38144723	38144799	CCTTGTGTTGTGTGTATTCAACTCACAGAGTTAAACGATCCTTTACACAGAGCAGATTTGAAACACTGTTTTTCTGG	2

chr16	38168845	38168921	CCTTGTGTTGTGTGTATTCAACTCACAGAGTTAAACGATCCTTTACACAGAGCAGATTTGAAACACTGTTTTTCTGG	2
chr16	38209287	38209363	CCTTGTGTTGTGTGTATTCAACTCACAGAGTTAAACGATCCTTTACACAGAGCAGATTTGAAACACTGTTTTTCTGG	2
chr16	38210986	38211062	CCTTGTGTTGTGTGTATTCAACTCACAGAGTTAAACGATCCTTTACACAGAGCAGATTTGAAACACTGTTTTTCTGG	2
chr16	38229667	38229743	CCTTGTGTTGTGTGTATTCAACTCACAGAGTTAAACGATCCTTTACACAGAGCAGATTTGAAACACTGTTTTTCTGG	2
chr16	47424037	47424114	CCATTCTCCCTATCACTTTCAGGTACACCAATCAAACGTAGGTTTGGTCTTTTCACATAGTCCCATATTTCTTGGAGG	1
chr16	60730549	60730625	CCTCGTCACTGCCAGATTTTGTGGCTACCAGCAAAGGATCGTTTTAAGCTGCAACTCAGGAAATTGAGAAAATATGG	2
chr16	72545014	72545091	CCTCCAAGAAATATGGGACTATGTGAAAAAACCAAACCTACGTTTGATTGGTGTACCTGAAAGTGACAGGGAGAATGG	1
chr16	81945503	81945579	CCCTGTGTTCTTTTATACTAAAACAAGCCAGCAAACCAACCTTTGAGATGTGTTGCCTTAAACATTACTGAATGGGG	2
chr16	81945503	81945578	CCCTGTGTTCTTTTATACTAAAACAAGCCAGCAAACCAACCTTTGAGATGTGTTGCCTTAAACATTACTGAATGGG	4
chr17	16474024	16474100	CCGAGAAACGGCTTTAGCAACAAATAAATATCAAAAGGATGCTTTCTCTTCAGAATAATCTAAAGTAAGTTGGGAGG	3
chr17	34438512	34438589	CCATGTTACTCCGGATAAGGACAGCAAAGGAGGAAAGGAACCTTTTCTGGGCCACCAGAAGGATGAGCTTGGGCTTGG	1
chr17	43690782	43690859	CCCAGGGATATGCTGGCCACGGGGAGGAGCCGGAAACCAACCTTTGTGTCACTGTGTAGTGACAAGTGCCTTTGGAGG	1
chr17	43690783	43690859	CCAGGGATATGCTGGCCACGGGGAGGAGCCGGAAACCAACCTTTGTGTCACTGTGTAGTGACAAGTGCCTTTGGAGG	2
chr17	69156298	69156375	CCTTAGGGACCCATAATGGCCACAACCAGGAGAAAAGCAAGCTTTGATGCTTAAACACTACTTACAGACATGTACAGG	1
chr17	74595228	74595305	CCTGCCTCTGTTCCTCCTTCCTGATGGTGGCGGAAAGGATGCTTTTGCCAGATCAACAGTCACACACAACACACCAGG	1
chr17	83191644	83191721	CCTGACTCCAGCCCTCCTTGACAAGGTCTCCGTAAAGCATGCTTTCTCTTAGGGACCCTCAGAGGGAGGCTTGGTGGG	1
chr17	83191644	83191720	CCTGACTCCAGCCCTCCTTGACAAGGTCTCCGTAAAGCATGCTTTCTCTTAGGGACCCTCAGAGGGAGGCTTGGTGG	3
chr18	35135224	35135300	CCTTATTTGGAATGTGACAAGACCCATTTGTTTAAACCTTGGTTTTTATGCAGAAAGAAAAGGAAGGCTGCAGTGGG	3
chr18	38918861	38918938	CCATTCTCCCTGTCACTTTCAGGTACACTAATCAAACGTAGGTTTGCTGTTTTTACATAGGCTCATATTTCTTGGAGG	1
chr18	45476589	45476666	CCATTCTCCCCATCACTTTCAGGTACACCAGTCAAACGTAGGTTTGGTCTTTTCACATAGTCCCATATTTCTTGGAGG	1
chr18	48640821	48640896	CCTGTTTGTTATTTTAGCTAATGTCAAAAAGAAAACCTTGCTTTTTCTGAACCCTTTCAGAGGCAGAAAGTGGGGG	4
chr18	71096732	71096808	CCATTTTCCCCACCACTTTCACGTACAGCAATCAAACGTAGGTTTGGTCTTTTCACTAGTCCCATATTTCTTGGAGG	3
chr19	24957844	24957920	CCTTGTAGTGTGTGTATTCAACTCACAGAGTTAAACGATCCTTTACACAGAGCAGACTTGAAACACTCTTGTTGTGG	2
chr19	25015316	25015392	CCTTGTAGTGTGTGTATTCAACTCACAGAGTTAAACGATCCTTTACACAGAGCATACTTGAAACACTCTTTTTGTGG	2
chr19	25074119	25074195	CCTTGTGTTGTGTGTATTCAACTCACAGAGTTAAACGATCCTTTACACAGAGCAGACTTGAAACACTCTTTTTGTGG	2
chr19	25827861	25827937	CCTTGTGTTGTGTTTATTCAACTCACAGAGTTAAACGATCCTTTACACAGAGCAGACTTGAAATACTCTTTTTGTGG	2
chr19	26054056	26054132	CCTTGTAGTGTGTGTATTCAACTCACAGAGTTAAACGATCCTTTACACAGAGCATACTTGAAACACTCTTTTTGTGG	2
chr19	26211777	26211853	CCTTGTATTGTGAGTATTCAACTCACAGAGTTAAACGATCCTTTACACAGAGCAGACTTGAAACACTCTTTTTGTGG	2
chr19	26483670	26483746	CCTTGTGTTGTGTGTCTTCAACTCACAGAGTTAAACGATGCTTTACACAGAGTAGACTTGAAACACTCTTTTTCTGG	2
chr19	26636516	26636592	CCTTGTGTTGTGTGTATTCAACTCACAGAGTTAAACGATCCTTTACACAGAGCAGACTTGTAACACTCTTTTTGTGG	2

chr19	26637877	26637953	CCTTGTGTTGTGTGTATTCAACTCACAGAGTTAAACGATCCTTTACACAGAGCAGACGTGAAACACTCTTTTTGTGG	2
chr19	26750223	26750299	CCTTGTGTTGTGTGTATTCAACTCACAGAGTTAAACGATCCTTTACACAGAGCAGACTTGAAACACTCTTTTTGTGG	2
chr19	26841158	26841234	CCTTGTGTTGTGTGTATTCAACTCACAGAGTTAAACGATCCTTTACACAGAGGAGACTTGTAACACTCTTTTTGTGG	2
chr19	28517220	28517297	CCAGGAAAAAATTTAAACTTTCTTAACTTGATAAAAGGTAGCTTTCAAAACCTACAATAAATAACATACTTAGAGTGG	1
chr19	34566821	34566898	CCATTCTCCTCGTCACTTTCAGGTACACCAAACAAACGTAGGTTTGGTCTTTTTACGTAGTCCCATATTTCTTGGAGG	1
chr19	52261770	52261847	CCCTCTTGAAGTTAGGGAAGTAGCATTTAAGGGAAACGTAGCTTTACTATTAAGAATTTCAAACAGCACTTGTCAGGG	1
chr19	52261770	52261846	CCCTCTTGAAGTTAGGGAAGTAGCATTTAAGGGAAACGTAGCTTTACTATTAAGAATTTCAAACAGCACTTGTCAGG	3
chr19	52261771	52261847	CCTCTTGAAGTTAGGGAAGTAGCATTTAAGGGAAACGTAGCTTTACTATTAAGAATTTCAAACAGCACTTGTCAGGG	2
chr19	52261771	52261846	CCTCTTGAAGTTAGGGAAGTAGCATTTAAGGGAAACGTAGCTTTACTATTAAGAATTTCAAACAGCACTTGTCAGG	4
chr20	11151392	11151469	CCATTCTCCCCGTCACTTTCAGGTACACCAATCAAACGTAGGTTTGGTCTTTTCACATATTCCCATATTTCTTGGAGG	1
chr20	14027067	14027143	CCATTCTCCCTTCACTTTCAGGTACACCAATCAAACGTAGGTTTGGTCTTTTCACATAGTCCCATATTTTTTGGAGG	2
chr20	50615399	50615476	CCTATAGTCTCAGTTACTTGGGAGGCTGAGGTAAAAGGATCGTTTGAGCCCAGGAGGTGGAGGTTGCAGTGAGCCGGG	1
chr20	50615399	50615475	CCTATAGTCTCAGTTACTTGGGAGGCTGAGGTAAAAGGATCGTTTGAGCCCAGGAGGTGGAGGTTGCAGTGAGCCGG	3
chr20	60909414	60909490	CCTTTCCCAACTCTGCTATTGCCCCCACATCCTAAAGGAACCTTTCTTTTTTTATATATTTTATTTTAAGTTCCAGG	3
chr21	16226086	16226163	CCTCCAAGAAATATGGAACTATGTGAAAAGACCAAACCTACGTTTGATTGACGTACCTGAAAGTGACAGGGAGAATGG	1
chr21	17835234	17835309	CCTCTTCTGAAAGCATTGATAATCAACATTTTAAACGTAGCTTTTCCCCATATTGCTAGGAAGGCTCATTCCCGGG	4
chr21	19425636	19425713	CCTCCAAGAAATATGGGACTATGTGAAAAGGCCAAACCTACGTTTGATTGCTGTACCCGAGAGTGACGGGGAGAATGG	1
chr21	32220958	32221035	CCTCCAAGAAATATGGGACTATGTGAAAAGACCAAACCTACGTTTGATTGGTGTACCTGAAAGTGATGGGGAGAATGG	1
chr21	34335877	34335953	CCCGGGGCCTGGGTGCCCAGTGCCAGTGGTCAGAAAGGTTGCTTTGGTGTTTTTCATTGTTAGTGAGACAGAGATGG	3
chr21	34335878	34335953	CCGGGGCCTGGGTGCCCAGTGCCAGTGGTCAGAAAGGTTGCTTTGGTGTTTTTCATTGTTAGTGAGACAGAGATGG	4
chr21	36315276	36315353	CCATTCTCCCCATCATTTTCAGGTACACCAATCAAACGTAGGTTTGATCTTTTCACATAGCCCCATATTTCTTGGAGG	1
chr21	41547952	41548028	CCACCAGCACTTCTGTTAGAAGTTGCAGCAGAGAAAGGATCCTTTAGGCACATCTCCCAGATCCTTGCGAAGAGGGG	3
chr22	18973194	18973271	CCTGTGCCAGGGTCCTTCCACTGGGACTGGCAGAAACGTAGGTTTGCATGGAGTGAGAAGCAGGGGAGAGGTTGAGGG	1
chr22	18973194	18973270	CCTGTGCCAGGGTCCTTCCACTGGGACTGGCAGAAACGTAGGTTTGCATGGAGTGAGAAGCAGGGGAGAGGTTGAGG	3
chr22	20265462	20265539	CCCTCAGCCTCTCCCCTGCTTCTCACTCCATGCAAACCTACGTTTCTGCCAGTCCCAGCAGAAGGACCCTGGCACGGG	1
chr22	20265462	20265538	CCCTCAGCCTCTCCCCTGCTTCTCACTCCATGCAAACCTACGTTTCTGCCAGTCCCAGCAGAAGGACCCTGGCACGG	3
chr22	20265463	20265539	CCTCAGCCTCTCCCCTGCTTCTCACTCCATGCAAACCTACGTTTCTGCCAGTCCCAGCAGAAGGACCCTGGCACGGG	2
chr22	20265463	20265538	CCTCAGCCTCTCCCCTGCTTCTCACTCCATGCAAACCTACGTTTCTGCCAGTCCCAGCAGAAGGACCCTGGCACGG	4
chrX	27300998	27301075	CCTCCAAGAAATATGGGGCTATGTGAAAAGACCAAACCTACCTTTGATTGGTGTATCTGAAAGTGACGGGGAGAATGG	1
chrX	28456666	28456743	CCTCCAAGAAATATGGGACTATGTGAAAAGACCAAACCTACGTTTGATTTGTGTACCTGAAAGTGATGGGGAGAATGG	1

chrX	35634985	35635062	CCATTCTCCCCGTCACTTTCAGGTACACCAATCAAACGTAGGTT TGGTCTTTTCTCATTGTCCCATATTTCTTGGAGG	1
chrX	39460148	39460223	CCCATCAAGAGCGGTTGTGCATGGCAACAGTAAAAGGATGGTTT GTTACACTAGTACAAAAAGAGGTGGCCAGAGG	4
chrX	43926403	43926480	CCATTCTCTCTGTCACTTTCAGGTACACCAATCAAACGTAGGTT TGGTCTTTTCACATAGTCCCATATTTCTTGGAGG	1
chrX	44254600	44254677	CCTCCAAGAAATACGGGACTATGTGAAAAGACCAAACGTACGTT TGATTGGTGTACCTGAAAGTGATAGGGAGAATGG	1
chrX	46088602	46088679	CCTCCAAGAAATATGGGACTATGTGAAAAGACCAAACCTACGTT TGATTGGTGTACCTGAAAGTGACTGGGAGAATGG	1
chrX	50222874	50222951	CCATTCTCCCTGTCACTTTCAGGTACACGAATCAAACGTAGGTT TCATCTTTTCACATAGTCCCATATTTCTTAGAGG	1
chrX	57416835	57416911	CCATTCTCTCTGTCACTTTCTGGTACACCAATCAAACGTAGGTT TGGTCTTTTCACATAGTTTCACATATTTCTTGG	3
chrX	57856466	57856543	CCTCCAAGAAATATGGGACTATGTGAAAAGACCAAACCTACGTT TGATTGGTGTACCTGAAAGTGACAAGGAAAATGG	1
chrX	62702479	62702556	CCTGAAAAACATTGTTTCCAACCTGGTAAATCAAAAGGAAGGTT TAACTTTGTTAGATAAGTCCACATATCACCAAGG	1
chrX	63067129	63067206	CCTCCAAGAAATGTGGGACTATGGGAAAAGACCAAACCTACCTT TGTTTGGTGTACCTGAAAGTGACGGGGAGAAAGG	1
chrX	64936250	64936327	CCTCCAAGAAATATGGGACTATGTGAAAAGACCAAACCTACGTT TCATTGGTGTACCTGAAAGTGATGGGTAGAATGG	1
chrX	66720099	66720176	CCTACAAGAAATATGGGACTATGGGAAAAGACCAAACCTACGTT TGATTGGTACACTGGAAAGTGACAGGGATAATGG	1
chrX	68529086	68529163	CCATTCTCCCTGTCACTTTCTGGTACACCAATCAAAGGTAGGTT TGGTCTTTTCACATAGTCCCATATTTCTTGGAGG	1
chrX	73893994	73894071	CCTCCAAGAAATATGGGACTATGTGAAAAGACCAAACCTACGTT TGATTGGTGTACCTGAAAGTGATGGGGAGAATGG	1
chrX	75723201	75723278	CCATTCTCTTTGTCACTTTCAGGTATACCAATCAAACGTTGGTT TGGTCTTTTTGCATAGTCCCATATTTTGTGGAGG	1
chrX	75815659	75815736	CCTCCAAGAAATATGAGACTATGTGAAAAGACCAAACCTACGTT TGATTAGTGTACCTGAAAATGATGGGGAGAATGG	1
chrX	80967103	80967180	CCATTCTTTCTGTCACTTTCAGGTACACCAATCAAACGTAGGTT TGGTCTTTTCACATAGTCCCATATTTCTTGGAGG	1
chrX	89936425	89936502	CCATTCTCCCTGTCACTTTCAGGTACACCAATCAAACGTAGGTT TGTTCTTTTCACATAGTCCCATATTTCTTGGAGG	1
chrX	91038768	91038845	CCATTATCCCCATCACTTTCAGGTACACCAATCAAACGTAGGTT TGGTTTTTTCACATAGTTCAATATTTCTTTGAGG	1
chrX	91471271	91471348	CCTCCAAGAAATATGGGACTATCTGAAAAGATCAAACCTACGTT TGATTGGTGTACCTGAAAGTGACAGGGAGAATGG	1
chrX	96428180	96428257	CCTTTCTCCCCATCACTTTCAGGTACACCAATCAAACGTAGGTT TGGTCTTTTCATATAGTCCCATATTTCTTGGAGG	1
chrX	100268291	100268368	CCTCCAAGAAATATGGGACTATGTGCAAAGATCAAACCTACGTT TGATTGCTGTACCTGAAAGTGATGGGGAGAATGG	1
chrX	105811046	105811123	CCATTCTCCCCATCACTTTCAGGTACACCAGTCAAACGTAGGTT TGGTCTTTTCACATAATCCCATATTTCTTGGAGG	1
chrX	115673065	115673141	CCTCCAAGAAGTATGGGACCATGGAAAAGATCAAACCTACGTTT GACTGGTGTACCTGAAAGTGACTGGGAGAATGG	2
chrX	117269846	117269923	CCTCCAAGAAATATGGGACTATGTGAAAAGACCAAACCTACGTT TGATTGGAGTACTTGAAAATGACAGGGATAATGG	1
chrX	139191369	139191445	CCTTTAAAGACATGCTCTTTGTGCCAGAAATTCAAAGGTTGCTT TTATGTCCAGTGGGGTGGAGGGAGGAAGCTCGG	3
chrX	147988614	147988691	CCATTCTCCCCGTCACTTTCAGGGACCTCAATCAAACGTAGGTT TTGTCTTTTCACATAGTCCCATATTTCTTGGAGG	1
chrX	155321041	155321118	CCTCCAAGAAATATAGGACTATGTGAAAAGACCAAACCTACGTT TGACTGGTGTACCTGAAAGTGACAGGGAGAATGG	1
chrY	15109391	15109468	CCATTCTCCCCATCACTTTCAGGTACACCAATCAAAGGTAGGTT TGGTCTTTTCACATAGTCCGATATTTCCTGCAGG	1

Chromosomal sites were identified by searching for $CCX_{(30\text{-}31)}$-AAASSWWSSTTT-$X_{(30\text{-}31)}$-GG where W is T or A and S is G or C. Pattern 1 is $CCX_{(31)}$-AAASSWWSSTTT-$X_{(31)}$-

GG 2 is CC$X_{(30)}$-AAASSWWSSTTT-$X_{(31)}$-GG, 3 is CC$X_{(31)}$-AAASSWWSSTTT-$X_{(30)}$-GG and 4 is CC$X_{(30)}$-AAASSWWSSTTT-$X_{(30)}$-GG. Only the + strand is shown and the start and end corresponds to the first and last base pair in the chromosome (GRCh38) or alternate assembly when applicable. Source code is described in Note 3.

Supplementary Table 8 is available for download at <https://goo.gl/Bisq30>.

For access while article is in review, please login using Username: LiuLabPrograms, Password: recCas9 upon reaching the webpage.

Methods for supporting material

Episomal DNA extraction and DNA sequencing

Transfections of HEK293T cells were performed under the conditions described in the methods of the main text. Episomal DNA was extracted using a modified HIRT extraction involving alkaline lysis and spin column purification essentially as described (*2, 3*). After harvesting, HEK293T cells were washed in 500 μL of ice cold PBS and resuspended in 250 μl GTE Buffer (50 mM glucose, 25 mM Tris-HCl, 10 mM EDTA and pH 8.0) incubated at room temperature for 5 minutes and lysed on ice for 5 minutes with 200 μl lysis buffer (200 mM NaOH, 1% sodium dodecyl sulfate). Lysis was neutralized with 150 μl of a potassium acetate solution (5M acetate, 3 M potassium, pH 6.7). Cell debris were pelleted by centrifugation at 21,130 x g for 15 minutes and lysate was applied to Econospin Spin columns (Epoch Life Science, Missouri City, TX), washed twice with 750 μl wash buffer (Omega Bio-tek, Norcross, GA), and eluted in TE 45 μl (pH 8.0).

Isolated episomal DNA was digested for 2 hours at 37 °C with RecBCD (10 U) per manufactures instructions and purified into 10 μL EB with a MinElute Reaction Cleanup Kit (Qiagen, Valencia, CA). Mach1-T1 chemically competent cells were transformed with 5 μL of episomal extractions and plated on agarose plates selecting for carbenicillin resistance. Individual colonies were sequenced with pCALNL-for1 to determine the rate of recombination. Sequencing reads revealed either the 'left' intact non-recombined recCas9 site, the expected recombined product, rare instances of 'left' non-recombined site with small indels, or one instance of a large deletion product.

Reporter construction

A five-piece Golden Gate assembly was used to construct reporters described in the text. Fragments 1-5 were flanked by Esp3I sites; Esp3I digestion created complementary 5' overhangs specifying the order of fragment assembly (Supplementary Figure 1). Fragments 1, 2, 4, and 5 were created by annealing forward and reverse complementary oligonucleotides listed in Table 5. Fragments were annealed by mixing 10 μl of each oligonucleotide (100 μM) in 20 μl of molecular grade water, incubating at 95°C for 3 minutes and reducing the temperature to 16 °C at a rate of -0.1 °C/sec. Fragment 3 was created by PCR amplifying the region containing kanR and a PolyA stop codon with primers 3-for and 3-rev. These primers also appended Esp3I on the 5' and 3' ends of this sequence.

Annealed fragments 1, 2, 4 and 5 were diluted 12,000 fold and 0.625 μl of each fragment were added to a mixture containing the following.

1) 40-50 ng fragment 3
2) 100 ng pCALNL EGFP-Esp3I
3) 1 μL Tango Buffer (10X)

4) 1 μL DTT (10 mM)
5) 1 μL ATP (10 mM)
6) 0.25 uL T7 ligase (3,000 U/μL)
7) 0.75 uL Esp3I (10 U/μL)
8) H20 up to 10 μL

Reactions were incubated in thermal cycler programmed for 20 cycles [37°C for 5 min, 20°C].

After completion of the Golden Gate reactions, 7 μL of each reaction was mixed with 1 μL of ATP (10 mM), 1 μL of 10X Plasmid Safe ATP-dependent DNAse buffer (10X), and 1 μL of Plasmid Safe ATP-dependent DNAse (10 U/μL) (Epicentre, Madison, WI) to remove linear DNA and reduce background. DNAse digestions were incubated at 37°C for 30 min and heat killed at 70°C for 30 min. Half (5 μL) of each reaction was transformed into Mach1-T1 cells. Colonies were analyzed by colony PCR and sequenced.

The protocol was modified for reporters used in Figure 4. Two gBlocks, encoding target sites to the 5' or 3' of the PolyA terminator were used instead of fragments 1, 2, 4 and 5. These gBlocks (10 ng) were added to the MMX, which was cycled 10 times [37°C for 5 min, 20°C] and carried forward as described above.

Note S1: DNA sequence of target sites flanking a KanR and PolyA stop sequences.

ACGCGT**CCC**CAATTTTCCCAAACAGAGGT*CTGTAAACCGAGGTTTTGGA*A
CCTCTGTTTGGGAAAATTG**GGG**AGTCGAGTCGGATTTGATCTGATCAAGA
GACAGGATGAGGATCGTTTCGCATGATTGAACAAGATGGATTGCACGCAG
GTTCTCCGGCCGCTTGGGTGGAGAGGCTATTCGGCTATGACTGGGCACA
ACAGACAATCGGCTGCTCTGATGCCGCCGTGTTCCGGCTGTCAG/TCGCA
GGGGCGCCCGGTTCTTTTTGTCAAGACCGACCTGTCCGGTGCCCTGAATG
AACTGCAGGACGAGGCAGCGCGGCTATCGTGGCTGGCCACGACGGGCG
TTCCTTGCGCAGCTGTGCTCGACGTTGTCACTGAAGCGGGAAGGGACTG
GCTGCTATTGGGCGAAGTGCCGGGGCAGGATCTCCTGTCATCTCACCTTG
CTCCTGCCGAGAAAGTATCCATCATGGCTGATGCAATGCGGCGGCTGCAT
ACGCTTGATCCGGCTACCTGCCCATTCGACCACCAAGCGAAACATCGCAT
CGAGCGAGCACGTACTCGGATGGAAGCCGGTCTTGTCGATCAGGATGAT
CTGGACGAAGAGCATCAGGGGCTCGCGCCAGCCGAACTGTTCGCCAGGC
TCAAGGCGCGCATGCCCGACGGCGAGGATCTCGTCGTGACCCATGGCGA
TGCCTGCTTGCCGAATATCATGGTGGAAAATGGCCGCTTTTCTGGATTCAT
CGACTGTGGCCGGCTGGGTGTGGCGGACCGCTATCAGGACATAGCGTTG
GCTACCCGTGATATTGCTGAAGAGCTTGGCGGCGAATGGGCTGACCGCTT
CCTCGTGCTTTACGGTATCGCCGCTCCCGATTCGCAGCGCATCGCCTTCT
ATCGCCTTCTTGACGAGTTCTTCTGAGCGGGACTCTGGGGTTCGAAATGA
CCGACCAAGCGACGCCCAACCTGCCATCACGAGATTTCGATTCCACCGCC
GCCTTCTATGAAAGGTTGGGCTTCGGAATCGTTTTCCGGGACGCCGGCTG
GATGATCCTCCAGCGCGGGGATCTCATGCTGGAGTTCTTCGCCCACCCCA
TCGATAACTTGTTTATTGCAGCTTATAATGGTTACAAATAAAGCAATAGCAT
CACAAATTTCACAAATAAAGCATTTTTTTCACTGCATTCTAGTTGTGGTTTG
TCCAAACTCATCAATGTATCTTATCATGTCTGGATCAAATCCGAACGCAC**C**
CCCAATTTTCCCAAACAGAGGT*CTGTAAACCGAGGTTTTGGA*ACCTCTGTT
TGGGAAAATTG**GGG**CTCGAG

A representative sequence containing a kan resistance marker (highlighted in green) and PolyA terminator (highlighted in red) flanked by two identical target sites. The target sites shown are both PAM_NT1-0bp-gix_core-0bp-NT1_PAM (see Table 1). Protoadjacent spacer motifs (PAMs) are in bold. Base pair spacers are lower case. Gix site or pseudo sites are in italics and dCas9 binding sites are underlined. For the genomic reporter plasmids used in the assays of Figure 4, a G to T transversion was observed in the kanamycin resistance marker, denoted by a G/T in the sequence above. This was present in all the reporters used in this figure, and we do not anticipate affecting the results as it is far remove from the PolyA terminator and recCas9 target sites.

Note S2. DNA and amino acid sequence of Ginβ-(GGS)$_8$-dCas9-GGS-FLAG-GGS-NLS.

DNA sequence of GinB-8GGS-dCas9-GGS-FLAG-GGS-NLS. Sequence encoding Ginβ is shown colored in green; those encoding GGS linkers are shown colored orange; those encoding dCas9 linkers are black; those encoding the FLAG tag and NLS are in blue and cyan respectively.

ATGCTCATTGGCTACGTGCGCGTCTCAACTAACGACCAGAATACCGATCTTCA
GAGGAACGCACTGGTTTGTGCAGGCTGCGAACAGATTTTCGAGGACAAACTCA
GCGGGACACGGACGGACAGACCTGGCCTCAAGCGAGCACTCAAGAGGCTGC
AGAAAGGAGACACTCTGGTGGTCTGGAAATTGGACCGCCTGGGTCGAAGCAT
GAAGCATCTCATTTCTCTGGTTGGCGAACTGCGAGAAAGGGGGATCAACTTTC
GAAGTCTGACGGATTCCATAGATACAAGCAGCCCCATGGCCGGTTCTTCTTC
TACGTGATGGGTGCACTGGCTGAAATGGAAAGAGAACTCATTATAGAGCGAAC
CATGGCAGGGCTTGCGGCTGCCAGGAATAAAGGCAGGCGGTTTGGAAGACCA
CCAAAGGGTGGATCCGGAGGGTCCGGAGGTAGTGGCGGCAGCGGTGGTTCA
GGTGGCAGCGGAGGGTCAGGAGGCTCTGATAAAAAGTATTCTATTGGTTTAGC
TATCGGCACTAATTCCGTTGGATGGGCTGTCATAACCGATGAATACAAAGTAC
CTTCAAAGAAATTTAAGGTGTTGGGGAACACAGACCGTCATTCGATTAAAAAGA
ATCTTATCGGTGCCCTCCTATTCGATAGTGGCGAAACGGCAGAGGCGACTCG
CCTGAAACGAACCGCTCGGAGAAGGTATACACGTCGCAAGAACCGAATATGTT
ACTTACAAGAAATTTTTAGCAATGAGATGGCCAAAGTTGACGATTCTTTCTTTCA
CCGTTTGGAAGAGTCCTTCCTTGTCGAAGAGGACAAGAAACATGAACGGCACC
CCATCTTTGGAAACATAGTAGATGAGGTGGCATATCATGAAAAGTACCCAACG
ATTTATCACCTCAGAAAAAAGCTAGTTGACTCAACTGATAAAGCGGACCTGAG
GTTAATCTACTTGGCTCTTGCCCATATGATAAAGTTCCGTGGGCACTTTCTCAT
TGAGGGTGATCTAAATCCGGACAACTCGGATGTCGACAAACTGTTCATCCAGT
TAGTACAAACCTATAATCAGTTGTTTGAAGAGAACCCTATAAATGCAAGTGGCG
TGGATGCGAAGGCTATTCTTAGCGCCCGCCTCTCTAAATCCCGACGGCTAGAA
AACCTGATCGCACAATTACCCGGAGAGAAGAAAAATGGGTTGTTCGGTAACCT
TATAGCGCTCTCACTAGGCCTGACACCAAATTTTAAGTCGAACTTCGACTTAGC
TGAAGATGCCAAATTGCAGCTTAGTAAGGACACGTACGATGACGATCTCGACA
ATCTACTGGCACAAATTGGAGATCAGTATGCGGACTTATTTTTGGCTGCCAAAA
ACCTTAGCGATGCAATCCTCCTATCTGACATACTGAGAGTTAATACTGAGATTA
CCAAGGCGCCGTTATCCGCTTCAATGATCAAAAGGTACGATGAACATCACCAA
GACTTGACACTTCTCAAGGCCCTAGTCCGTCAGCAACTGCCTGAGAAATATAA
GGAAATATTCTTTGATCAGTCGAAAAACGGGTACGCAGGTTATATTGACGGCG
GAGCGAGTCAAGAGGAATTCTACAAGTTTATCAAACCCATATTAGAGAAGATG
GATGGGACGGAAGAGTTGCTTGTAAAACTCAATCGCGAAGATCTACTGCGAAA
GCAGCGGACTTTCGACAACGGTAGCATTCCACATCAAATCCACTTAGGCGAAT
TGCATGCTATACTTAGAAGGCAGGAGGATTTTTATCCGTTCCTCAAAGACAATC
GTGAAAAGATTGAGAAAATCCTAACCTTTCGCATACCTTACTATGTGGGACCCC
TGGCCCGAGGGAACTCTCGGTTCGCATGGATGACAAGAAAGTCCGAAGAAAC
GATTACTCCATGGAATTTTGAGGAAGTTGTCGATAAAGGTGCGTCAGCTCAAT
CGTTCATCGAGAGGATGACCAACTTTGACAAGAATTTACCGAACGAAAAAGTAT

TGCCTAAGCACAGTTTACTTTACGAGTATTTCACAGTGTACAATGAACTCACGA
AAGTTAAGTATGTCACTGAGGGCATGCGTAAACCCGCCTTTCTAAGCGGAGAA
CAGAAGAAAGCAATAGTAGATCTGTTATTCAAGACCAACCGCAAAGTGACAGT
TAAGCAATTGAAAGAGGACTACTTTAAGAAAATTGAATGCTTCGATTCTGTCGA
GATCTCCGGGGTAGAAGATCGATTTAATGCGTCACTTGGTACGTATCATGACC
TCCTAAAGATAATTAAAGATAAGGACTTCCTGGATAACGAAGAGAATGAAGATA
TCTTAGAAGATATAGTGTTGACTCTTACCCTCTTTGAAGATCGGGAAATGATTG
AGGAAAGACTAAAAACATACGCTCACCTGTTCGACGATAAGGTTATGAAACAG
TTAAAGAGGCGTCGCTATACGGGCTGGGGACGATTGTCGCGGAAACTTATCAA
CGGGATAAGAGACAAGCAAAGTGGTAAAACTATTCTCGATTTTCTAAAGAGCG
ACGGCTTCGCCAATAGGAACTTTATGCAGCTGATCCATGATGACTCTTTAACCT
TCAAAGAGGATATACAAAAGGCACAGGTTTCCGGACAAGGGGACTCATTGCAC
GAACATATTGCGAATCTTGCTGGTTCGCCAGCCATCAAAAAGGGCATACTCCA
GACAGTCAAAGTAGTGGATGAGCTAGTTAAGGTCATGGGACGTCACAAACCG
GAAAACATTGTAATCGAGATGGCACGCGAAAATCAAACGACTCAGAAGGGGCA
AAAAAACAGTCGAGAGCGGATGAAGAGAATAGAAGAGGGTATTAAAGAACTGG
GCAGCCAGATCTTAAAGGAGCATCCTGTGGAAAATACCCAATTGCAGAACGAG
AAACTTTACCTCTATTACCTACAAAATGGAAGGGACATGTATGTTGATCAGGAA
CTGGACATAAACCGTTTATCTGATTACGACGTCGATGCCATTGTACCCCAATCC
TTTTTGAAGGACGATTCAATCGACAATAAAGTGCTTACACGCTCGGATAAGAAC
CGAGGGAAAAGTGACAATGTTCCAAGCGAGGAAGTCGTAAAGAAAATGAAGAA
CTATTGGCGGCAGCTCCTAAATGCGAAACTGATAACGCAAAGAAAGTTCGATA
ACTTAACTAAAGCTGAGAGGGGTGGCTTGTCTGAACTTGACAAGGCCGGATTT
ATTAAACGTCAGCTCGTGGAAACCCGCCAAATCACAAAGCATGTTGCACAGAT
ACTAGATTCCCGAATGAATACGAAATACGACGAGAACGATAAGCTGATTCGGG
AAGTCAAAGTAATCACTTTAAAGTCAAAATTGGTGTCGGACTTCAGAAAGGATT
TTCAATTCTATAAAGTTAGGGAGATAAATAACTACCACCATGCGCACGACGCTT
ATCTTAATGCCGTCGTAGGGACCGCACTCATTAAGAAATACCCGAAGCTAGAA
AGTGAGTTTGTGTATGGTGATTACAAAGTTTATGACGTCCGTAAGATGATCGCG
AAAAGCGAACAGGAGATAGGCAAGGCTACAGCCAAATACTTCTTTTATTCTAAC
ATTATGAATTTCTTTAAGACGGAAATCACTCTGGCAAACGGAGAGATACGCAAA
CGACCTTTAATTGAAACCAATGGGGAGACAGGTGAAATCGTATGGGATAAGGG
CCGGGACTTCGCGACGGTGAGAAAAGTTTTGTCCATGCCCCAAGTCAACATAG
TAAAGAAAACTGAGGTGCAGACCGGAGGGTTTTCAAAGGAATCGATTCTTCCA
AAAAGGAATAGTGATAAGCTCATCGCTCGTAAAAAGGACTGGGACCCGAAAAA
GTACGGTGGCTTCGATAGCCCTACAGTTGCCTATTCTGTCCTAGTAGTGGCAA
AAGTTGAGAAGGGAAAATCCAAGAAACTGAAGTCAGTCAAAGAATTATTGGGG
ATAACGATTATGGAGCGCTCGTCTTTTGAAAAGAACCCCATCGACTTCCTTGAG
GCGAAAGGTTACAAGGAAGTAAAAAAGGATCTCATAATTAAACTACCAAAGTAT
AGTCTGTTTGAGTTAGAAAATGGCCGAAAACGGATGTTGGCTAGCGCCGGAGA
GCTTCAAAAGGGGAACGAACTCGCACTACCGTCTAAATACGTGAATTTCCTGT
ATTTAGCGTCCCATTACGAGAAGTTGAAAGGTTCACCTGAAGATAACGAACAG
AAGCAACTTTTTGTTGAGCAGCACAAACATTATCTCGACGAAATCATAGAGCAA
ATTTCGGAATTCAGTAAGAGAGTCATCCTAGCTGATGCCAATCTGGACAAAGTA
TTAAGCGCATACAACAAGCACAGGGATAAACCCATACGTGAGCAGGCGGAAAA
TATTATCCATTTGTTTACTCTTACCAACCTCGGCGCTCCAGCCGCATTCAAGTA

TTTTGACACAACGATAGATCGCAAACGATACACTTCTACCAAGGAGGTGCTAG
ACGCGACACTGATTCACCAATCCATCACGGGATTATATGAAACTCGGATAGATT
TGTCACAGCTTGGGGGTGACGGTGGCTCCGATTATAAGGATGATGACGACAA
GGGAGGTTCCCCAAAGAAGAAAAGGAAGGTCTGA

Amino acid sequence of the GinB-8GGS-dCas9-GGS-FLAG-GGS-NLS. The color scheme is the same as the DNA sequence.

MLIGYVRVSTNDQNTDLQRNALVCAGCEQIFEDKLSGTRTDRPGLKRALKRLQKG
DTLVVWKLDRLGRSMKHLISLVGELRERGINFRSLTDSIDTSSPMGRFFFYVMGAL
AEMERELIIERTMAGLAAARNKGRRFGRPPKGGSGGSGGSGGSGGSGGSGGSG
GSDKKYSIGLAIGTNSVGWAVITDEYKVPSKKFKVLGNTDRHSIKKNLIGALLFDSG
ETAEATRLKRTARRRYTRRKNRICYLQEIFSNEMAKVDDSFFHRLEESFLVEEDKK
HERHPIFGNIVDEVAYHEKYPTIYHLRKKLVDSTDKADLRLIYLALAHMIKFRGHFLI
EGDLNPDNSDVDKLFIQLVQTYNQLFEENPINASGVDAKAILSARLSKSRRLENLIA
QLPGEKKNGLFGNLIALSLGLTPNFKSNFDLAEDAKLQLSKDTYDDDLDNLLAQIG
DQYADLFLAAKNLSDAILLSDILRVNTEITKAPLSASMIKRYDEHHQDLTLLKALVRQ
QLPEKYKEIFFDQSKNGYAGYIDGGASQEEFYKFIKPILEKMDGTEELLVKLNREDL
LRKQRTFDNGSIPHQIHLGELHAILRRQEDFYPFLKDNREKIEKILTFRIPYYVGPLA
RGNSRFAWMTRKSEETITPWNFEEVVDKGASAQSFIERMTNFDKNLPNEKVLPKH
SLLYEYFTVYNELTKVKYVTEGMRKPAFLSGEQKKAIVDLLFKTNRKVTVKQLKED
YFKKIECFDSVEISGVEDRFNASLGTYHDLLKIIKDKDFLDNEENEDILEDIVLTLTLF
EDREMIEERLKTYAHLFDDKVMKQLKRRRYTGWGRLSRKLINGIRDKQSGKTILDF
LKSDGFANRNFMQLIHDDSLTFKEDIQKAQVSGQGDSLHEHIANLAGSPAIKKGILQ
TVKVVDELVKVMGRHKPENIVIEMARENQTTQKGQKNSRERMKRIEEGIKELGSQI
LKEHPVENTQLQNEKLYLYYLQNGRDMYVDQELDINRLSDYDVDAIVPQSFLKDD
SIDNKVLTRSDKNRGKSDNVPSEEVVKKMKNYWRQLLNAKLITQRKFDNLTKAER
GGLSELDKAGFIKRQLVETRQITKHVAQILDSRMNTKYDENDKLIREVKVITLKSKLV
SDFRKDFQFYKVREINNYHHAHDAYLNAVVGTALIKKYPKLESEFVYGDYKVYDVR
KMIAKSEQEIGKATAKYFFYSNIMNFFKTEITLANGEIRKRPLIETNGETGEIVWDKG
RDFATVRKVLSMPQVNIVKKTEVQTGGFSKESILPKRNSDKLIARKKDWDPKKYG
GFDSPTVAYSVLVVAKVEKGKSKKLKSVKELLGITIMERSSFEKNPIDFLEAKGYKE
VKKDLIIKLPKYSLFELENGRKRMLASAGELQKGNELALPSKYVNFLYLASHYEKLK
GSPEDNEQKQLFVEQHKHYLDEIIEQISEFSKRVILADANLDKVLSAYNKHRDKPIR
EQAENIIHLFTLTNLGAPAAFKYFDTTIDRKRYTSTKEVLDATLIHQSITGLYETRIDL
SQLGGDGGSDYKDDDDKGGSPKKKRKVStop

Note S3. Source code for recCas9 genomic target identification

All software scribed in this manuscript is available for download at <https://goo.gl/Bisq30>. For access while this manuscript is in review, please login using Username = LiuLabPrograms and Password = recCas9

Note S4. Limiting dilution quantification of recCas9 genomic deletion

As described in the Methods, we can infer the number of genomes present in a given amount of template DNA, and thus estimate a minimum deletion efficiency for recCas9 at the *FAM19A2* locus. For example, take the case of a two-fold dilution series, beginning with 20 ng genomic DNA template. After nested PCR, only the well seeded with 20 ng yielded the correct PCR product. At 3.6 pg per genome, that PCR contained approximately 5500 genomes, and since at least one recombined genome must have been present, the minimum deletion efficiency is 1 in 5500 or 0.018%.

We chose to quantify the levels of genomic DNA using a limiting dilution of genomic template because using quantitative PCR (qPCR) to determine the absolute level of genome editing would require a set of PCR conditions that unambiguously and specifically amplify only from post-recombined genomic DNA. As shown in Figure 5B, primary PCR using genomic DNA as template results in a roughly 2.5 kb off-target band as the dominant species; a second round of PCR using nested primers is required to reveal guide RNA- and recCas9-dependent genome editing.

References for supporting material

1. N. E. Sanjana *et al.*, A transcription activator-like effector toolbox for genome engineering. *Nature protocols* **7**, 171-192 (2012).
2. K. Motmans, S. Thirion, J. Raus, C. Vandevyver, Isolation and quantification of episomal expression vectors in human T cells. *Biotechniques* **23**, 1044-1046 (1997).
3. B. Hirt, Selective extraction of polyoma DNA from infected mouse cell cultures. *J Mol Biol* **26**, 365-369 (1967).
